# Four-million-year Marinoan snowball shows multiple routes to deglaciation

**DOI:** 10.1073/pnas.2418281122

**Published:** 2025-04-21

**Authors:** Adrian R. Tasistro-Hart, Francis A. Macdonald, James L. Crowley, Mark D. Schmitz

**Affiliations:** ^a^Department of Earth and Planetary Science, McCone Hall, University of California, Berkeley, CA 94720; ^b^Department of Geosciences, Boise State University, Boise, ID 83725

**Keywords:** geochronology, paleoclimate, Cryogenian, stratigraphy

## Abstract

Twice during the Neoproterozoic Era, Earth experienced runaway ice-albedo catastrophes that resulted in multimillion year, low-latitude glaciations: the Sturtian and Marinoan snowball Earths. In the snowball climate state, CO_2_ consumption through silicate weathering collapses, and atmospheric CO_2_ accumulates via volcanic outgassing until a sufficiently strong greenhouse causes deglaciation. The duration and extent of ice cover are critical for planetary habitability, both on exoplanets and on Earth where animals emerged between the two glaciations. Radioisotopic ages have defined the duration of the Sturtian glaciation to 56 Myr, but the duration of the Marinoan glaciation (4 to 15 Myr) currently has 11 Myr of uncertainty. Here, we show that the Marinoan glaciation in Namibia lasted ca. 4 Myr with less than 10 m of vertical ice grounding line motion through glacial advance-retreat cycles. The stability of a low-latitude ice grounding line is consistent with the strong hysteresis of a hard snowball state. The disparity in durations demonstrates different routes to deglaciation, through slower CO_2_ accumulation for the longer Sturtian and radiative perturbation for the Marinoan. The short duration of the Marinoan glaciation may have been essential for the survival and evolution of animals and illustrates an additional path toward habitability on exoplanets.

## Significance

Twice during the Neoproterozoic Era, ice covered most of the Earth in so-called “snowball” glaciations. The first glaciation, the Sturtian, extended to the equator and lasted 56 My. However, the severity and duration of the second, the Marinoan, are unknown, with previous studies suggesting Quaternary-like orbitally forced ice cyclicity. Drone imagery anchored with field observations of Marinoan glacial deposits in Namibia demonstrates a stable, long-lived ice grounding line that was insensitive to orbital forcing, consistent with the strong hysteresis of the snowball state. Radioisotopic dates on these deposits constrain the duration of the Marinoan glaciation to 4 My. The dramatic difference in duration between the Neoproterozoic snowballs indicates that there are different pathways to exit the snowball hysteresis.

Energy balance and general circulation models show that planets are susceptible to runaway ice-albedo catastrophes, referred to as snowball climate states (1–4). These catastrophes are possible within the conventionally defined habitable zone (5), imposing important constraints on long-term evolutionary pathways. At least twice during the Cryogenian Period (ca. 717–635 Mya) of the Neoproterozoic Era, ice extended to tropical latitudes for millions of years in presumed snowball paleoclimatic events (6–8). Geochronology has demonstrated that the Sturtian glaciation occurred between 717–661 Ma and the Marinoan started sometime after 651 Ma, before 639 Ma, and ended at 635 Ma, lasting 4 to 16 Myr (*SI Appendix*, Fig. S1 and citations therein). These age constraints have provided a first-order test of the snowball Earth hypothesis for Earth’s paleoclimate by demonstrating a multimillion year duration and globally synchronous terminations (9, 10), but large uncertainty remains in the onset age of the Marinoan, which is important for reconstructing the climate state and testing mechanisms for deglaciation.

In a snowball Earth, CO_2_ consumption through silicate weathering is diminished, and atmospheric CO_2_ builds up from sustained volcanic outgassing until a critical threshold is reached for deglaciation. The deglaciation threshold is strongly dependent on planetary albedo (4). If the Marinoan was closer to the maximum 16 Myr, then the difference in duration with the ca. 56 Myr Sturtian is more easily reconciled with modest changes in CO_2_ outgassing or silicate weathering between the two events (11). Moreover, this would imply a short duration between the two events and that the Earth may have rapidly returned to a snowball state due to the background balance of CO_2_ sources and sinks (12). In contrast, a Marinoan duration closer to 4 Myr may be explained by a stochastic perturbation to albedo allowing for deglaciation at lower CO_2_ level (4, 13, 14), possibly in combination with a hard snowball lacking ocean-atmosphere equilibration and the associated seafloor weathering sink for CO_2_(11).

**Fig. 1.**
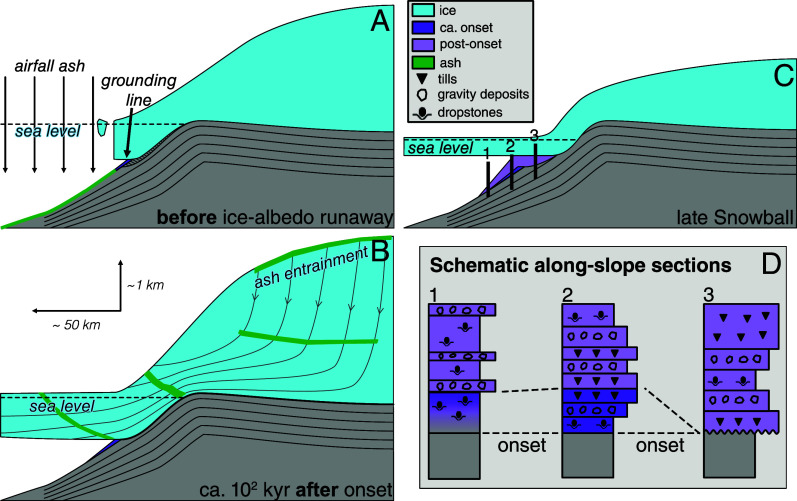
Idealized ice growth and stratigraphy at onset of snowball Earth. Note vertical exaggeration. (*A*) Marine-terminating terrestrial ice sheet with calving ice front prior to ice-albedo runaway into snowball glaciation. The green band shows accumulation of airfall tuff. (*B*) Schematic of a terrestrial ice sheet at its furthest advance flowing into the snowball sea glacier shortly after the ice-albedo runaway onset of snowball glaciation. The earliest glaciomarine sediments (dark purple) accumulate beyond the furthest advance of the ice down the foreslope. Also shown is entrainment of volcanic ash from the accumulation zone of the ice sheet to the base of the sea glacier. (*C*) Later in the snowball, ice has thinned due to gradual global warming. In this scenario, local sea-level fall from glacioisostatic rebound and loss of ice gravity has balanced ice thinning and tectonic subsidence, maintaining a fixed grounding line. Sediments accumulating since panel (*B*) are shown in light purple. (*D*) Idealized stratigraphies at three sections in panel (*C*). In 1, the lowest stratigraphic dropstones only provide a minimum constraint on onset. In 2, a conformable and unambiguous record of initial ice advance is preserved. In 3, the initial ice advance has introduced hiatus via erosion and nondeposition, precluding a direct sedimentary record of onset.

Direct dating of glacial onset is challenging due to the scarcity of sections that preserve a conformable record of initial ice advance. For clarity, “snowball onset” refers to the runaway growth of high-albedo sea ice to low latitudes. In an idealized snowball onset at low-latitude, a large sea-level fall precedes ice advance on the continental slope, due to the growth of high latitude terrestrial ice (15, 16). At some location along the slope, grounded ice must reach its furthest advance, shortly after snowball onset when temperatures are coldest and ice is thickest (Fig. 1*B*) (10, 17). The stratigraphy at such a location would ideally show a sequence recording sea-level fall and this initial advance. In this idealized model, the first glacial sediments are dropstones melting from a floating or calving ice shelf, coarsening into the distal redeposited sediments of the advancing grounding zone wedge, and finally into lodgment till deposited directly beneath grounded ice (Fig. 1 *B* and *C*).

## Ghaub Formation Stratigraphy and Geochronology

**Fig. 2.**
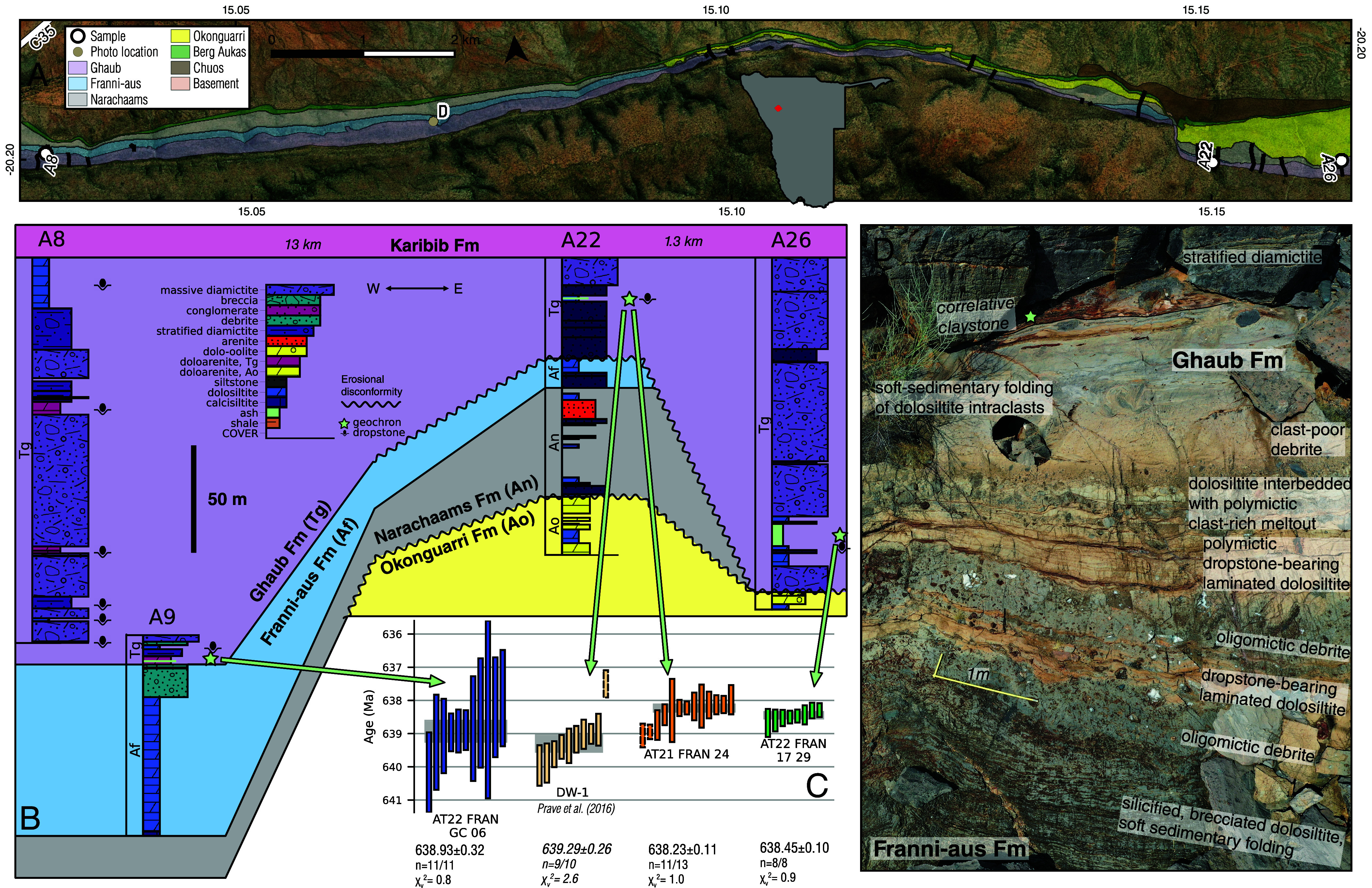
Representative stratigraphy and geochronology at Fransfontein Ridge. (*A*) Geologic map of Cryogenian formations at Fransfontein Ridge, with sections and sampling locations. Sections plotted below are labeled. Measured sections and drone imagery documenting contact relationships are available in *SI Appendix*. (*B*) Sections with dated horizons along the ridge. AT21 FRAN 24 is a resampling of DW-1 reported by ref. 20. The lowest stratigraphic evidence for grounded ice in *SI Appendix*, section A9 is massive diamictite 9.7 m above sample AT22 FRAN GC 06, and thus the date provides an age on the transition between the Franni-aus and the Ghaub formations. (*C*) Rank-age plot of ^206^Pb/^238^U dates; all analyses were concordant (*SI Appendix*, section S3). Weighted mean ages are shown as gray rectangles. Bars with dashed outlines were not included in the weighted mean. (*D*) Drone image of contact from Franni-aus Fm to stratified basal Ghaub Fm, showing interbedding of oligomictic gravity flows with dropstone-bearing laminated dolosiltites that mark the first glacigenic sedimentation. See panel (*A*) for location. Claystone correlative with AT22 FRAN GC 06 is labeled at the *Top* of the exposure, cf. *SI Appendix*, Fig. S21. The lowest stratigraphic evidence for grounded ice occurs 10.4 m above the claystone horizon at the *Top* of the image.

The Marinoan Ghaub Formation (Fm) along Fransfontein Ridge in Namibia preserves an oblique transect of a grounding zone wedge on the foreslope of the southern paleomargin of the Congo paleocontinent (Fig. 2*A*) (18, 19), offering a rare record of snowball onset. A tuffaceous siltstone within the Ghaub Fm provides the existing minimum age constraint for ca. 639 Ma Marinoan onset (20) and confirms the presence of dateable ashes within the stratigraphy. These features, in addition to excellent outcrop of well-documented grounding line motion (19), make Fransfontein Ridge a promising location for dating Marinoan onset and constraining timescales of glaciomarine sedimentary dynamics.

In the eastern study area, Ghaub Fm rests disconformably on Narachaams and Okonguarri Fms, whereas to the west it rests on Franni-aus Fm (Fig. 2*B*), a down-slope thickening wedge of coarsening-upward, redeposited oligomictic carbonate rhythmites and debrites lacking any evidence for glacigenic sedimentation. The Franni-aus Fm has been previously interpreted as a falling-stand wedge deposit associated with glacioeustatic sea-level fall during the Marinoan onset (18). The Franni-aus Fm hosts the Trezona negative carbon isotopic anomaly, recognized also in Canada and Australia. On all three continents, the Trezona anomaly immediately underlies Marinoan glacial diamictites, and in the Trezona Fm dropstones are present at the top (21, 22). Carbon isotopes in the Franni-aus Fm in the west preserve nearly 100 m of recovery to 0‰ following the Trezona anomaly (*SI Appendix*, Fig. S6), attesting to an expanded record of immediately preglacial carbonate deposition, especially compared to eastern exposures where the recovery is absent due to erosional truncation up-slope. Above the Franni-aus Fm, the basal Ghaub Fm consists of polymictic dropstone-bearing laminated dolosiltites and stratified diamictites recording progrounding line accumulation from suspension settling, traction currents, gravity flows, and meltout of englacial debris (*SI Appendix*, section S2.A).

Further lines of evidence suggest that the contact between the basal Ghaub and Franni-aus formations in the western study area is conformable. Characteristic oligomictic debrites (Franni-aus Fm) and polymicitic dropstone bearing dolosiltites (Ghaub Fm) are locally interbedded (Fig. 2*D* and *SI Appendix*, Figs. S12 and S13). Both the Franni-aus and basal Ghaub Fms preserve soft-sedimentary deformation that we and ref. 19 interpret as glacigenic in response to ice loading and motion; we interpret this ice advance to be the initial one. These features attest to the nonlithified nature of Franni-aus Fm during Ghaub Fm time, providing further evidence against a significant hiatus between the two formations (*SI Appendix*, Figs. S14, S15, and S17–S19). For these reasons, we interpret the basal Ghaub Fm in the western study area to provide a sedimentary record of the initial ice advance at Fransfontein Ridge in response to the onset of the Marinoan snowball glaciation.

Three tuffaceous samples were collected from Ghaub Fm and dated via high-precision chemical abrasion isotope dilution thermal ionization mass spectrometry. Weighted mean dates (Fig. 2*C*) span ca. 639–638 Ma. Two samples come from the eastern, up-slope exposures (*SI Appendix*, Fig. S3 and section S2.B), and another sample comes from a western, down-slope outcrop (*SI Appendix*, Fig. S4 and section S2.A). All samples exhibit unimodal geochemical and age populations (*SI Appendix*, Figs. S20 and S24), and the weighed mean dates are interpreted to record eruptive ages within uncertainty.

The ash yielding the oldest weighted mean date of 638.93 ± 0.32 Ma comes from the western study area, 3.8 m above a gradational contact with the Franni-aus Fm and below the first evidence for locally grounded ice. We interpret this ash to have deposited prior to the initial advance of locally grounded ice, and it therefore constrains the down-slope expansion of low latitude terrestrial ice at the onset of the Marinoan snowball (Fig. 1*D*-2).

**Fig. 3.**
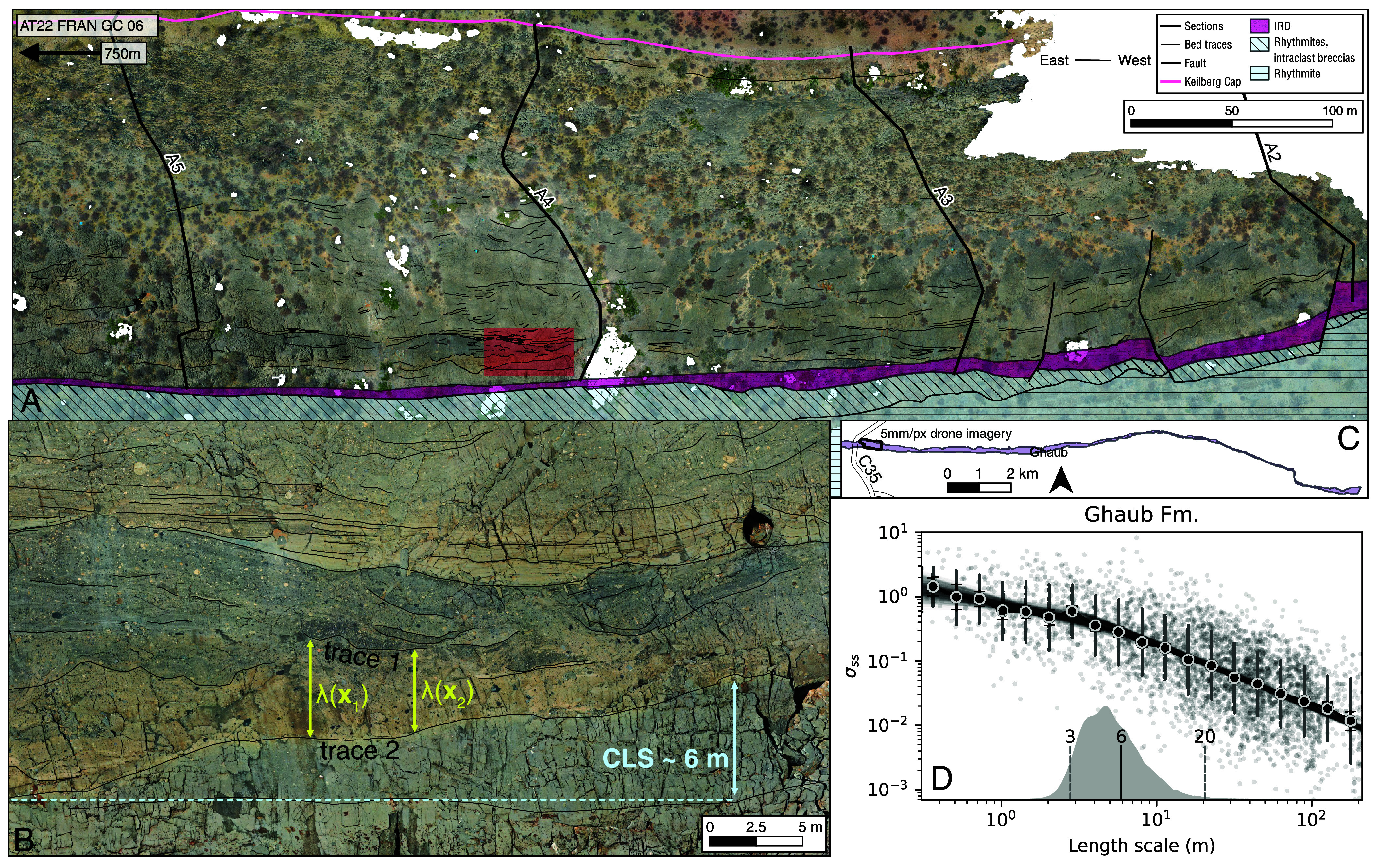
(*A*) Over 400 bedding contacts traced onto 5 mm/px drone imagery of Ghaub Fm. (*B*) Zoomed-in view of red area in panel (*A*). Two traces are labeled in yellow along with separations $\lambda (x_{1})$ and $\lambda (x_{2})$, illustrating how these distances contribute to computation of *σ*_*ss*_ (Eq. **1**). In blue, the median value of 6 m for the CLS is annotated where it approximately corresponds to erosional relief along a contact between buff matrix massive diamictite above and gray matrix massive diamictite below. (*C*) Overview map of study area showing extent of 5 mm/px imagery on Ghaub Fm exposure. (*D*) Spatial variability in sedimentation, *σ*_*ss*_, from bed traces of Ghaub Fm. Each dot corresponds to *σ*_*ss*_ evaluated between a single pair of traces. Black dots show geometric means within bins of length scale. Gray bars show single geometric SD; black bars show geometric SE. Translucent black lines show Markov chain Monte Carlo fits to binned data. Gray kernel density estimate shows posterior distribution for CLS; 3 to 20 m spans the 95% credible interval.

The other two samples, both younger, come from the eastern study area, where Ghaub Fm sits on an erosional disconformity with Narachaams and Okonguarri Fms (Fig. 2 and *SI Appendix*, Figs. S2 and S3). In this area, glacial erosion occurred prior to the deposition of basal Ghaub Fm (*SI Appendix*, section S2.B), so the ashes postdate the initial snowball ice advance. The dates therefore provide minimum onset constraints (Fig. 1*D*-3). The easternmost sample yields a date of 638.45 ± 0.10 Ma (*SI Appendix*, section S3.B.3), and a reconnaissance sample from the same horizon yields a statistically indistinguishable weighted mean date (*SI Appendix*, section S3.B.4).

We also resampled and reanalyzed the horizon previously dated by ref. 20. Our reanalysis yields a ca. 1 Myr younger date of 638.23 ± 0.11 Ma (Fig. 2*C* and *SI Appendix*, section S3.B.2). This weighted mean excludes the two oldest analyses, which overlap with the youngest reported by ref. 20. We note that the large MSWD of 2.6 for their weighted mean indicates excessive dispersion for a single-age population. In conjunction with the overlap in the tails of the age populations between the two sets of analyses, we interpret geologic age dispersion in the sample, likely resulting from protracted crystallization of zircon. The MSWD of 1.0 that we obtain over our 11 youngest grains is more consistent with these grains constituting a shortly pre-eruption magmatic population.

Much of the underlying stratigraphy was previously interpreted to be Ghaub Fm (18, 20), but this interval dominated by mass flow deposits is reassigned to the Narachaams Fm, reducing the thickness of Ghaub Fm in this section (Fig. 2*B* and *SI Appendix*, section S2.B). With our reanalysis of the ash originally reported by ref. 20, we find that both ashes in the eastern study area are younger than the western sample, consistent with hiatus of a few hundred thousand years at a sub-Ghaub Fm disconformity on the upper foreslope.

### Ash Delivery and Age Interpretation.

The most likely source for the ashes are the volcanic arcs that developed on the Coastal Terrane of the western Kaoko zone and correlative Punte del Este terrane in South America (23). Detrital zircon distributions in the southern Kaoko zone show large, consistent Cryogenian peaks with youngest grains at ca. 640 to 650 Ma (24) in sections with distal Marinoan glacial sediments. These young zircon grains have been argued to be sourced from an impinging arc from the west.

Airborne ashes from volcanic arcs on the Coastal Terrane could have been deposited either through direct airfall through open water or entrainment into the ice (Fig. 1). Entrainment of airfall ash on the accumulation zone of the terrestrial snowball ice sheet provides a transport mechanism for the syn-snowball tuffs (*SI Appendix*, section S5). For entrainment, the eruptive age constrains the timing of ash deposition on an ice sheet. Entrained ash beds transit ice sheets as coherent bands (25, 26), which travel according to the flow of the ice. Ash layers have been reported as cm-scale bands from ice cores of the Ross Ice Shelf (27) and have been modeled to have transitted hundreds of kilometers through the ice shelf, potentially having accumulated on the terrestrial ice sheet (28). Particle paths of ice flow models for Antarctic ice sheets flowing into ice shelves (28–30) are schematically reproduced in Fig. 1*B*, with the only major difference being that Antarctic ice shelves are zones of accumulation, rather than ablation as Fig. 1*B* depicts. After transiting the ice sheet, the ash bands would be released via basal ice melting and deposit in front of the grounding line (Fig. 1*B*). The intersection of ash bands with the basally melting portion of the floating ice would likely occur diachronously due to variation in ice flow, resulting in spatially discontinuous accumulations and potentially stratigraphic repetition of the same ash. With the entrainment model, the dates, which we interpret as eruptive ages, would correspond to the age of deposition on the accumulation zone of a marine-terminating terrestrial ice sheet. In West Antarctica and Greenland, most ice, especially near the continental margins, is 10 to 100 kyr old (31, 32); thus sedimentary ages would likely only lag eruptive ages by 10 to 100 kyr.

The eastern, syn-snowball samples are most consistent with the entrainment mechanism due to the predicted presence of thick floating ice that would obstruct airfall (17). The stratigraphic expression of the ashes is also consistent with the entrainment model. The deposit reported by ref. 20 occurs as patchy, pod-like accumulations of green claystone, which occur at several distinct intervals within the same ca. 2 m thick package of stratified glacial sediment. These discontinuous deposits are nevertheless traceable over at least half a kilometer along strike (*SI Appendix*, Fig. S3) and are consistent with the diachronous intersection and melting out of ash bands at the base of the floating ice.

The western sample, which we interpret to precede the initial ice advance and which yields the oldest date, is more continuous than the eastern ashes. For this sample, airfall deposition is permissible because the ash occurs within an interval containing only ice meltout debris, which may have been rafted by icebergs rather than a floating ice shelf. This model requires the existence of a marine terminating, terrestrial ice sheet with a calving ice front at Fransfontein Ridge before the global ocean ice-albedo collapse into a snowball (33) (Fig. 1*A*). Under this airfall interpretation, the dated horizon would strictly predate snowball onset. Duration would be 3.7 Myr, assuming that the Keilberg cap dolostone at Fransfontein Ridge deposited synchronously with cap dolostones dated to ca. 635.2 Ma elsewhere (34) (*SI Appendix*, section S1).

Alternatively, this ash may have also deposited via entrainment through a marine-terminating terrestrial ice sheet shortly after snowball onset. Refs. 33 and 35 both demonstrate that tropical ice sheets grow quickly, achieving most of their volume within 200 to 300 kyr after snowball onset. In this case, the ash would postdate snowball onset by up to 200 to 300 kyr, which is small relative to snowball duration and comparable to the uncertainty on the date. We conservatively interpret a Marinoan duration of ca. 4 Myr, accounting for the potential lag of ice sheet growth in the entrainment model, which we cannot distinguish from the equally permissible airfall model.

## Timescales of Grounding Line Cycles

A ca. 4 Myr duration of the Marinoan glaciation is an order of magnitude shorter than the ca. 56 Myr of the preceding Sturtian. This result could be interpreted to imply that the Marinoan glaciation was not a snowball, especially since Ghaub Fm exhibits cyclic deposition that in Svalbard and Oman has been argued to be similar to Cenozoic-style orbitally forced ice mass variability (36, 37). This stratigraphic variability presents a challenge for the snowball hypothesis, which requires a weak hydrologic cycle (17, 35). Ref. 37 modeled the precession-like forcing of ice sheets in a hard snowball state, which at high atmospheric CO_2_ demonstrated significant ice mass variability in apparent reconciliation of an active hydrological cycle and the snowball Earth hypothesis. Ref. 37 suggest that cyclic snowball records primarily record deglaciation. Sensitivity to orbital forcing, however, implies a high-frequency responsiveness of global climate whereby orbital forcing could trigger deglaciation (38). This high-frequency sensitivity is difficult to reconcile with the long-lived Sturtian snowball.

The precise chronology presented here constrains the timescale of Marinoan glacial processes and climate dynamics at Fransfontein Ridge. In particular, these data show that the ca. 10 advance-retreat cycles preserved within the Ghaub Fm (*SI Appendix*, Fig. S5) span ca. 4 Myr. Assuming constant sedimentation, the number of cycles observed could be consistent with an origin in long eccentricity (405 kyr), but not obliquity or precession. Alternatively, the cycles might be obliquity or precession-forced, but only record a small fraction of the total snowball, explaining the small number preserved. Because of the difficulty in directly testing orbital timescales, an alternative method for evaluating the orbital hypothesis was developed. This method utilizes the compensation length scale (CLS), a statistical quantity that measures the maximum topographic relief that arises through the process of sedimentation (39). For example, in fluvial systems, the depth of the deepest channel is interpreted to set the CLS (39, 40).

In glaciomarine settings, the locus of sedimentation is the grounding line (Fig. 1*A*). Lateral retreat of the grounding line creates vertical accommodation, which can be filled by progradation of the grounding zone wedge (Fig. 1*B* and *SI Appendix*, Fig. S32). Lateral advance of the grounding line introduces the possibility of erosion beneath grounded ice. Thus, the CLS in this setting—analogous to channel depth in a fluvial system—is the maximum vertical amplitude of grounding line motion as it advances and retreats.

If ice sheets are sensitive to orbital forcing, the grounding line will advance and retreat dramatically, and the CLS should be on the order of dozens to hundreds of meters. Bathymetric profiles show precisely this scale of relief associated with grounding zone retreat since the Last Glacial Maximum in both the eastern Ross Sea (41) and the Gulf of St. Lawrence (42). The Gulf of St. Lawrence provides an end-member corresponding to total deglaciation, but in West Antarctica, where the ice sheet persists, the vertical relief is at least 60 m over the surveyable ice-free seafloor (41). Given much larger volumes of terrestrial snowball ice (15), the sensitivity of the snowball state to orbital forcing should result in a similar amplitude grounding line motion, yielding a CLS on the order of dozens to hundreds of meters.

Because Fransfontein Ridge provides an approximately margin-parallel exposure (18), the stratigraphic cut is well situated for resolving along-strike sedimentary relief resulting from the dynamics of glaciomarine sedimentation. The excellent outcrop and textural preservation of Ghaub Fm in the western part of our study area affords a rare opportunity to estimate CLS from bed traces on drone imagery (Fig. 3) (40). Our data define a 95% credible interval of 3 to 20 m for the CLS, with a median value of 6 m (Fig. 3*D*). Although this analysis has limitations–traced contacts cover only 750 m of outcrop at a single location–there is no evidence elsewhere along the ridge for deep, multidozen meter glacial erosion associated with any grounding line cycle within Ghaub Fm. Where traces are mapped, they resolve the geometries of relief on erosional surfaces, which dictate the CLS (Fig. 3*B*).

The median CLS value of 6 m is significantly smaller than the predicted large amplitude vertical motion of the grounding line in response to Cenozoic-style orbitally forced oscillations in ice sheet volume. This result implies that the record of grounding line motion preserved in Ghaub Fm is inconsistent with sensitivity to orbital forcing. Instead, these cycles may be autogenic, corresponding to minor shifts in the grounding line in response to the complex interplay over ca. 4 Myr of tectonic subsidence, sediment supply, ice thinning, ice gravity, and glacioisostatic rebound.

These data suggest that the grounding line was fixed over the entire Marinoan snowball at Fransfontein Ridge, further supported by the up-slope tapering of glacial sediments to zero thickness just 27 km to the east (*SI Appendix*, Fig. S2) (18). This apparent stability suggests that sediment supply and local relative sea-level fall—processes that cause grounding line advance—effectively balanced the effects of tectonic subsidence and ice thinning, which cause grounding line retreat. Local relative sea-level fall would result from the loss of ice gravity and glacioisostatic rebound under the assumption that nearby terrestrial ice mass decreased with gradual planetary warming over the course of the snowball. It is not obvious that such equilibrium in grounding location should persist for 4 Myr, given the varying timescales and potentially time-varying magnitudes of each process’s effect on grounding line position. Future studies should investigate how grounding line trajectories can evolve by exploring coupled models of solid Earth deformation, ice sheet evolution, tectonic subsidence, and snowball climate.

The stability of the grounding line at Fransfontein Ridge is consistent with a hard snowball climate state lacking Cenozoic-style ice ages. We argue that it is also inconsistent with a waterbelt snowball state in which thin sea ice or ice-free conditions exist in a narrow swatch of low latitudes (43–45). Although Fransfontein Ridge lacks reliable Cryogenian paleomagnetic constraints (*SI Appendix*, section S8), it hosts thick carbonate platform successions immediately before and after the Marinoan snowball, requiring subtropical to tropical latitudes. While a higher paleolatitude could permit a waterbelt interpretation, recent work demonstrates the instability of waterbelt solutions with more realistic ocean and sea glacier models (46, 47).

## Routes to Deglaciation

**Fig. 4.**
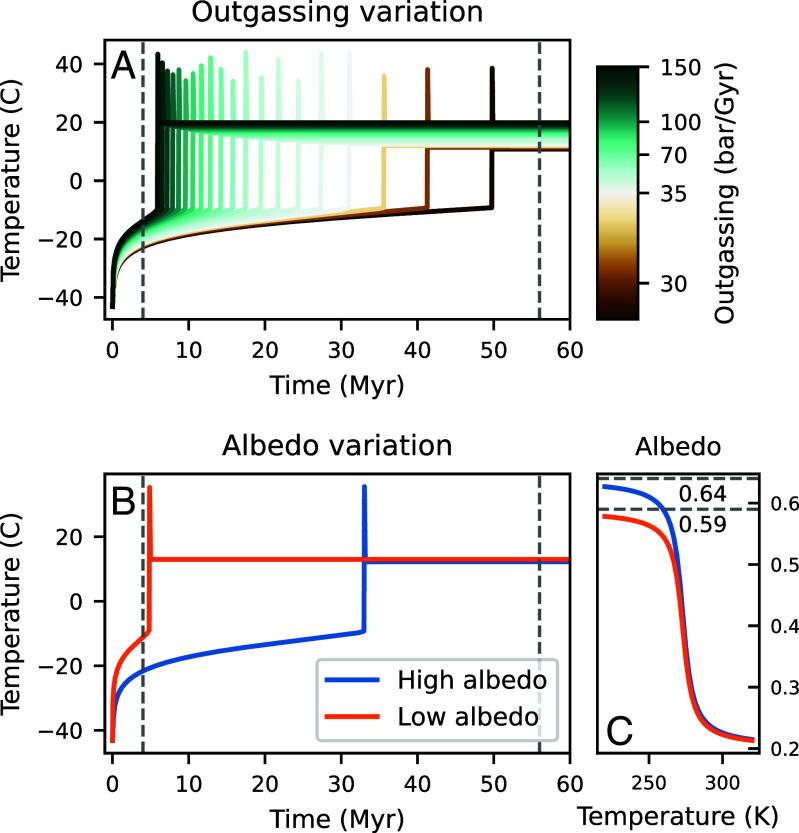
0D modeling of snowball climate; snowball states initiated at 230° K with 0.0003 bar atmospheric CO_2_. In (*A* and *B*), temperature evolution is plotted, and deglaciations are observed as the abrupt increases in temperature. (*A*) Note logarithmic scaling for outgassing rates. Small relative decreases in outgassing are capable of significantly lengthening a snowball, whereas outgassing must increase by several factors to shorten snowball duration. Gray dashed lines show Marinoan and Sturtian durations. (*B*) snowball duration decreases by order of magnitude for minor (0.05) decrease in planetary albedo. (*C*) High (blue) and low (orange) models of planetary albedo as a function of temperature, which differ by 0.05.

The extreme difference in duration between Sturtian and Marinoan snowballs was likely due to differences in the radiative balance and carbon cycle components of global paleoclimate. Assuming constant outgassing through time, the logarithmic increase of global temperature means that small radiative perturbations that change the atmospheric CO_2_ threshold for deglaciation can dramatically shorten snowball duration (4) (Fig. 4*B*). Radiative perturbations are most easily driven by minor changes in planetary albedo, for instance in response to the dustiness of the snowball (48), which, in addition to lowering albedo, can warm surface temperatures via absorption of reflected shortwave radiation (49). For example, a 20° band of latitude around the equator of dusty ice with albedo 0.3, as modeled by ref. 49, decreases planetary albedo from 0.64 to 0.59 and snowball duration by an order of magnitude (Fig. 4). Large eruptions have been invoked as triggers for deglaciation via dust dispersal (14).

Minor perturbations to the carbon cycle, on the other hand, are capable of dramatically extending snowball duration. Small decreases (5 to 10 bar Gyr^−1^) in outgassing can extend duration by tens of millions of years, whereas outgassing must increase by several factors to significantly reduce snowball duration (Fig. 4*A*). Slower outgassing resulting from decreased oceanic spreading has been invoked to explain the long duration of the Sturtian (50). While ref. 50 only quantify mid-ocean ridge outgassing, their modeled decrease of ca. 23 to ca. 7 Mt C yr^−1^ from the Tonian to the Sturtian corresponds to a decrease of 7.5 bar Gyr^−1^. Carbon outgassing remains poorly constrained even in the modern, where present uncertainties on the various contributions to outgassing yield an envelope that includes the full range of values explored in Fig. 4*A* (51). Alternatively, the presence of a syn-snowball sink for carbon has a similar effect as a decrease in outgassing. In the absence of chemical weathering of the continents and burial of organic carbon, the main carbon sink is weathering of seafloor basalt (11). This sink requires air–sea exchange of carbon through areas of open water (11), and is pH-dependent, strengthening as the ocean acidifies with higher levels of atmospheric CO_2_. A ca. 4 Myr Marinoan results in a 22 Myr interval between the snowball glaciations, allowing for more time to accommodate global tectonic reconfigurations that may have increased outgassing rates during the shorter Marinoan.

The shorter path to Marinoan deglaciation would have limited snowball stress on the biosphere to ca. 4 Myr, which is critical to the survival and diversification of nascent animals from the Cryogenian to Early Ediacaran. The 56 Myr Sturtian is associated with anoxia and the return of iron formations in the geological record (10), suggesting oxygen depletion that would have imposed stress on life. A shorter duration Marinoan would not have depleted oxygen as completely—consistent with a lack of Marinoan iron formations—which may have been critical for the survival of animals with higher metabolic demands for oxygenation. Finally, a shorter route to deglaciation provides a new path toward habitability on planets beyond Earth.

## Materials and Methods

### Field Work and Geochronology.

We measured 26 sections along 18 km of the foreslope exposures of Fransfontein Ridge (*SI Appendix*, Fig. S8), which augment dozens of sections summarized by ref. 18 along over 60 km (*SI Appendix*, Fig. S2). We collected 2 cm/px drone imagery along 16 km of the study area, which assisted in detailed mapping of units and surfaces. Higher-resolution 5 mm/px imagery was collected over the best exposures of Ghaub Fm grounding line cycles (Fig. 3). Full details of drone imagery collection are in *SI Appendix*, section S6. Mapping and sections were supplemented by carbon isotope chemostratigraphy (*SI Appendix*, Fig. S6) along 11 sections to aid in unit correlation.

We collected 11 samples of siltstone and arenite within Ghaub and underlying Franni-aus and Narachaams Fms for U-Pb zircon geochronology (*SI Appendix*, section S3). Rock samples underwent conventional mineral separation methods to extract zircon crystals. Initial analysis via laser ablation split stream inductively coupled plasma mass spectrometry (LASS-ICPMS) geochronology allowed us to identify three samples from Ghaub Fm with dominant young zircon populations (Fig. 2 and *SI Appendix*, Fig. S24), which also exhibited unimodal trace element geochemistry (*SI Appendix*, Fig. S20). We analyzed a subset of zircon grains for each sample via CA-ID-TIMS to establish more precise ages. Full details of the geochronology are in *SI Appendix*, section S3.A.

### Compensation Length Scale.

The CLS is a quantity relevant to sedimentation in any depositional setting, and it can be directly estimated from the stacking geometry of sediments exposed in outcrop. Quantification of the CLS has thus far only been performed for fluviodeltaic stratigraphies, where it is typically interpreted to reflect the depth of the deepest channel in the depositional system (39, 40, 52–54). We propose that the CLS is set by the vertical amplitude of grounding line motion in the glaciomarine realm, which we constrain for the Ghaub Fm. Ref. 39 defined the SD of sedimentation *σ*_*ss*_, which is reformulated by ref. 40 as

[1] $$\sigma_{\mathrm{ss}}\left(L\right)={\left[\int_{A}{\left(\frac{\lambda (x,L)}{L}-1\right)}^{2}dA\right]}^{\frac{1}{2}}\propto L^{-\kappa },$$

where *λ* is the thickness of stratigraphy between a pair of bedding contacts as a function of location **x** and *L* is the average thickness between the bedding contacts. Integrating over an outcrop area *A*, $\sigma_{\mathrm{ss}}\left(L\right)$ quantifies how stratigraphic thicknesses vary as a function of stratigraphic (i.e., vertical) length scale *L*. This quantity follows a power law with exponent less than one below the CLS and one above it (39, 40). Bed traces of sufficiently small spacing (smaller than the CLS) that vertically span at least three times the CLS can be used to estimate $\sigma_{\mathrm{ss}}\left(L\right)$ and infer the transition in power law behavior, i.e., the CLS (40, 53).

The 5 mm/px imagery sufficiently resolves the often subtle and complex bedding contacts exhibited by the glaciomarine stratigraphy. We projected this imagery onto a plane perpendicular to bedding (parallel to the younging direction), ensuring that thicknesses on the resulting orthomosaic corresponded to true stratigraphic thicknesses. and manually traced 404 contacts on the resulting mosaic. Heights were corrected for sediment compaction. These contacts were used to evaluate *σ*_*ss*_ for every pair of traces. The scattered *σ*_*ss*_ values were averaged within bins. The transition between compensational and subcompensational behavior of Eq. **1** was inferred via Bayesian probabilistic modeling to generate an estimate of the CLS. Full details of the CLS estimation are in *SI Appendix*, section S7.

### 0D Climate Modeling.

We implement the coupled radiative balance and silicate weathering model formulated by ref. 55. This model captures the fundamental snowball hysteresis, so we use it to explore the sensitivity of snowball duration to various parameters, such as outgassing and albedo. All parameters are as specified by ref. 55 except that we set *V* (volcanic outgassing) and *W*_*w*_ (the limit of chemical weathering in the warm state of the planet) both 35 bar Gyr^−1^. This choice of *W*_*w*_ permitted snowball durations of 60 Myr for suppressed outgassing rates (Fig. 4*A*).

We initiated the model in a snowball state with 230° K with 0.0003 bar atmospheric CO_2_, and then we followed the time evolution of temperature and atmospheric CO_2_ in the dynamical system. We modeled the effect of variations in outgassing rate, which were always assumed to be constant over the duration of each model (Fig. 4*A*). Variation in outgassing rate can be used to model a temperature-independent carbon sink, such as seafloor weathering (11). Such a sink is mathematically equivalent to a negative outgassing term. Seafloor weathering has been argued to be pH-dependent, with pH decreasing over the course of a snowball as the ocean acidifies in response to ever-growing CO_2_ levels in the atmosphere (11). In this case, the carbon sink term would grow in time; our assumption of a constant sink term in the form of outgassing perturbations thus provides a lower bound on the effect of seafloor weathering on extending snowball duration. We also modeled the effect of different albedos on snowball duration (Fig. 4*B*).

## Supplementary Material

Appendix 01 (PDF)

Dataset S01 (XLSX)

Dataset S02 (XLSX)

Dataset S03 (XLSX)

Dataset S04 (XLSX)

## Data Availability

Data and analyses are provided by ref. 56.
