# Supplementary material for: Four-million-year Marinoan snowball shows multiple routes to deglaciation
Source: Proc Natl Acad Sci U S A. 2025 Apr 21;122(18):e2418281122. doi: 10.1073/pnas.2418281122 (PMC12067226; doi:10.1073/pnas.2418281122)
Supplement: Supplementary file 1 — Appendix 01 (PDF) [file pnas.2418281122.sapp.pdf]

Supporting Information for

Four Million Year Marinoan Snowball Shows Multiple Routes to Deglaciation

Adrian R. Tasistro-Hart, Francis A. Macdonald, James L. Crowley, and Mark D. Schmitz

Adrian R. Tasistro-Hart
E-mail: adrian@tasistro-hart.com

This PDF file includes:

- Supporting text
- Figs. S1 to S38
- Tables S1 to S2
- Legends for Dataset S1 to S4
- SI References

Other supporting materials for this manuscript include the following:

- Datasets S1 to S4

Supporting Information Text

S1. Cryogenian Geochronology

Existing Cryogenian geochronology based on radioisotopic geochronometers is summarized in Figure S1. The references noted in the figure are repeated here: (1–36).

The ages are plotted as reported in the published manuscripts. One exception is the ca. 645 Ma age from ref. (13) from 8 m from the top of the Fiq Formation in the Lahan-1 sediment core. In the manuscript, the authors do not firmly offer an age and uncertainty for what they interpret to be a maximum depositional age constraint. We plot the weighted mean age of the youngest two concordant grains, 645.0±0.34 Ma, as the maximum depositional age constraint. Given the dispersion in ages of young zircon grains in this sample, as well as the small number of young grains, it is unlikely that this age is syn-depositional. Its location high in the Marinoan stratigraphy also renders it largely irrelevant for constraining Snowball onset.

Other constraints relevant to the onset of the Marinoan glaciation are discussed below.

The CA-ID-TIMS U-Pb zircon age of 651.7±0.6 Ma reported by ref. (15) originates from a detrital sample in pre-Marinoan Thorndike submember of the Kingston Peak Formation (Fm). While proximal to diamictite of the overlying Marinoan Wildrose submember, the sample is also below an erosional disconformity. This sample is therefore a maximum depositional age for a maximum age constraint. The Thorndike submember also lacks the Trezona carbonate carbon isotope anomaly—observed in Namibia, Australia, and Canada—that immediately precedes onset of the Marinoan glaciation (37–39). It is therefore difficult to link the dated horizon to Marinoan snowball onset; several million years could be missing.

A Marinoan onset age of 650 Ma (not plotted in Figure S1) reported by ref. (40) is modeled from cyclostratigraphically constrained sedimentation rates for Datangpo Fm, which the authors argue is conformable and gradational into the overlying Marinoan Nantuo Fm. This cyclostratigraphy, however, lacks any radioisotopic constraint.

S2. Fransfontein Stratigraphy

Fransfontein Ridge in Namibia provides a several dozen kilometer long oblique platform-to-slope transect that exposes Cryogenian to Ediacaran stratigraphy (41–44). The entire Cryogenian is represented along Fransfontein Ridge, with outcrops of both Sturtian and Marinoan glacial deposits as well as intervening Middle Cryogenian strata. The Sturtian glaciation is recorded by the Chuos Fm, which outcrops discontinuously in localized, fault-bounded mini-basins along the ridge. Along the ridge, Chuos Fm sits everywhere on basement. The stratigraphy recording the Middle Cryogenian exhibits significant thickness variability along the ridge, related to the development of a carbonate platform (north-northeast) as well as syn-sedimentary extensional faulting. Stratigraphic nomenclature varies for the slope and platform stratigraphy. The Marinoan glaciation is recorded by the Ghaub Fm, which is thickest in a well-developed grounding zone wedge on the paleo-foreslope (42).

Our field work focused on the slope sections along approximately 17 km of Fransfontein Ridge (Figures S4, S3, S2), where the Ghaub Fm is thickest. We measured several dozen stratigraphic sections, augmenting the dozens measured by ref. (41), and through several sections we sampled carbonates for stable isotope geochemistry (Table S1, Figure S6). The 2 cm px^{-1} drone imagery aided in detailed mapping of Ghaub Fm along the study area.

Along this panel, the Middle Cryogenian stratigraphy comprises Berg Aukas, Okonguarri, Narachaams, and Franni-aus Fms (41). The Berg Aukas Fm, the Sturtian cap carbonate, is composed of dark grey to black colored, finely-laminated and frequently brecciated dolosiltite and microbialite. The thickness of Berg Aukas Fm is consistently 15–30 m across the study area. Okonguarri Fm is characterized by light grey, graded beds of dolosiltite and doloarenite, including oolites, with intraclast breccia and conglomerate. Okonguarri Fm exhibits thickness variability on the order of hundreds of meters (Figure S2). Narachaams Fm is a siliciclastic dominated interval comprised of green siltstone and fine-grained sandstone, with subordinate arkosic arenite, dolostone conglomerate, and dolosiltite. Finally, Franni-aus Fm comprises cm-dm scale dolosiltite (with rare basal calcisiltite intervals), which form increasingly abundant tabular intraclast breccia up-section. Silicification also increases up-section, including clasts of oolites, which are commonly silicified. Franni-aus Fm preserves the Trezona carbonate carbon isotope anomaly (41), which has been associated with glacial onset elsewhere (38, 39). Ref. (41) interpret Franni-aus Fm to record a falling stand wedge accumulating during global sea level fall associated with buildup of terrestrial ice sheets immediately prior to the onset of the Marinoan snowball glaciation.

Except for Berg Aukas Fm, the units corresponding to the Middle Cryogenian exhibit substantial thickness variation along the study area. Okonguarri Fm thins from a several hundred-meter thick package in the east to zero towards the west, and vice-versa for Narachaams and Franni-aus Fms. This thickness variability indicates either lateral facies change and/or the presence of disconformities vis-à-vis Narachaams to Okonguarri Fm. Drone-aided mapping and stratigraphy, along with chemostratigraphy, suggests the presence of a previously undescribed disconformable surface with up to dozens of meters of relief between Okonguarri and Narachaams Fms (Figure S7). This disconformity is important for interpreting the stratigraphy in the eastern portion of the study area (Figure S3, Section S2.B), where much of what we interpret as Narachaams Fm has been interpreted as Ghaub Fm on the basis of this disconformable surface, which there is attributed to glacial erosion (33, 41). The DW-1 sample from ref. (33) originates from this panel, as does our resampling of DW-1 and two additional samples (AT22 FRAN 17 29 & MN1502 13). Interpretation of the relationship between these ages and Marinoan onset depends on understanding the complex underlying stratigraphy.

Our dated samples come from the western and eastern ends of study area: three were sampled from the eastern panel as mentioned above, and one (AT22 FRAN GC 06) was sampled from the western exposure 1.5 km east of the C-35 road. In the depositional model of ref. (41), the western exposures reflect lower foreslope deposition, whereas the eastern exposures move

progressively up the slope towards the platform, just beyond our study area. Below, we discuss the stratigraphy at the two sampling locations to provide context for interpreting the ages.

A. West Fransfontein Stratigraphy. The panel sampled in the western portion of the study area (Figure S4) exposes readily interpretable units that we unambiguously assign to Berk Aukas, Narachaams, Franni-aus, and Ghaub Fms, in agreement with ref. (41) (Figure S2). Here, Berg Aukas Fm rests directly on basement. Okonguarri Fm is absent in this panel, and the supra-Okonguarri channelized disconformity surface described above implies the presence of a corresponding but hitherto unidentified surface within this panel. Narachaams Fm consists of poorly exposed siltstone with medium- and coarse-grained arenites at its base. Franni-aus Fm has a sharp contact on Narachaams Fm and consists of 1–10 cm graded dolosiltite beds, which exhibit soft sedimentary folding and form intraclast breccias (Figures S11G). Both folding and brecciation increase in frequency up-section, as does silicification of the dolosiltites. Sedimentary breccias, interpreted as debris flows (debrites), increase in abundance towards the top of Franni-aus Fm and incorporate clasts of oolite, many of which are silicified (Figure S11G, H).

At map scale, the contact between Franni-aus and Ghaub Fms is clear and unambiguous (Figure S4), and in section it proves to be a conformable (Figure S12B, main manuscript Figure 2D, Figures S12A, S13) contact marked by the appearance of laminated dolosiltites containing dropstones, which are interbedded with oligomictic debrites characteristic of Franni-aus Fm. In Figure S12B, the clast protruding from an oligomictic debrite provides a clear example of conformity. It could be a dropstone, in which case the underlying Franni-aus Fm debrite would have clearly been unlithified in Ghaub Fm time due to the penetration depth of the clast. Alternatively, it deposited as part of the debrite, forming significant positive relief at the top of the deposit. This relief is then onlapped by the basal Ghaub Fm dolosiltite, demonstrating the absence of a preceding ice advance that would have almost certainly toppled the protruding clast.

Basal Ghaub Fm is almost always marked by the appearance of dropstones (Figure S11E & F), indicating pro-grounding line deposition (main manuscript Figure 2D, Figure S12). Ghaub Fm then coarsens with increasingly frequent debrites interbedded with meltout detritus (Figure S11E), channel forms (Figure S11D), and aggradational doloarenites (Figure S11A), consistent with local advance of the grounding zone wedge (e.g., Section A8 in Figure S21). The first sedimentary evidence for locally grounded ice—massive matrix supported diamictites locally overlying erosionally truncated soft sedimentary folds in stratified diamictites (Figure S11C)—always occurs above a sequence of pro-grounding line sediments, providing a consistent record of an initial ice advance.

This sequence characterizes Ghaub Fm over 10 km of the western exposures of the study area, from section A1 furthest west to east of section 16 from ref. (41) (main manuscript Figure 2D, Figures S12, S13). Furthermore, basal Ghaub Fm in these western sections always exhibits negative Franni-aus-like carbon isotope values (Sections A1 & A9, Figure S6), indicating that the material being redeposited is sourced from the immediately pre-glacial Ombaatjie Fm further up-slope. This pattern is in contrast to basal Ghaub Fm further east, which (barring the olistolith of Franni-aus Fm in Section A24) exhibits highly variable carbon isotopic values, including positive values, indicating deposition of material eroded deeper from underlying formations higher on the foreslope or platform (Figure S6). The gradational contact with Franni-aus Fm, the consistently distal-to-proximal character of glacial sediments in basal Ghaub Fm, and the lack of carbon isotopic evidence for significant glacial erosion all support the interpretation that basal Ghaub Fm in the western portion of the study area provides a conformable record of the initial Marinoan snowball ice advance at Fransfontein Ridge.

At one location along the western exposures of Ghaub Fm, ref. (42) report a "grooved" morphology on the top surface of Franni-aus Fm (their Figure 12G) (Figure S16). They reference this surface when interpreting a supra-Franni-aus Fm erosional disconformity—mapped along strike from the putative grooves—implying that it originated in direct contact with grounded ice. Just one meter east of the grooved surface, however, there is a lobe of Franni-aus Fm that does not have a ridged or grooved morphology (Figure S19). This lobe demonstrates the lack of continuity in the groove morphology and attests to an irregular surface of the underlying Franni-aus Fm debrite.

Furthermore, Franni-aus Fm is strongly folded in this panel (Figure S17B). The folding appears to be associated with early faulting (Figures S16, S17, S18). This faulting must have been early, because the faults do not offset beds higher in Ghaub Fm, which instead seals the faults. Basal Ghaub Fm in this panel is stratified with dropstones, and this basal interval is strongly disrupted and brecciated adjacent to the faults (Figure S18). While the folding and faulting could very well be glaciotectonic in origin, resulting from a local ice advance, we do not observe evidence for grounded ice prior to the basal stratified interval, which is always offset and disrupted by faults where they occur. Instead, the faulting, and possibly much of the folding, postdate the accumulation of the pro-grounding line sediments that constitute basal Ghaub Fm.

Given the total absence of grooves on the top of Franni-aus Fm elsewhere along the ridge, as well as the close association of this single groove occurrence with abundant soft sedimentary deformation of upper Franni-aus Fm, we conclude that the groove is likely also soft sedimentary deformation associated with motion of grounded ice above the stratified interval of basal Ghaub Fm. As ref. (42) also point out, the apparently glacially-induced soft sedimentary deformation observed in Franni-aus Fm implies that it was not strongly lithified prior to local ice advance, in turn suggesting that local ice advance occurred shortly after Franni-aus Fm time. The observation that this deformation is shared with the earliest Ghaub Fm sediments also implies that they accumulated shortly after Franni-aus deposition, in alignment with our interpretation of onset for AT22 FRAN GC 06.

B. East Fransfontein Stratigraphy. The stratigraphy in the eastern panel of the study area is expanded with respect to the western panel. It exhibits multiple erosional surfaces and abundant slope instability deposits—which are difficult to distinguish from glacigenic sediments—that preclude confident assignment of the basal Ghaub Fm contact (Figure S3). In this panel,

Chuos Fm sits on basement in a small fault bounded half-graben, and is dominated by massive brown diamictites with primarily granitoid clasts, although some stratified intervals occur throughout. Berg Aukas Fm blankets Chuos Fm and basement and consists of sub-decimeter beds of dolosiltite and fine-grained doloarenite.

A fold and fault dissects the eastern panel and separates a panel of readily interpretable units in the west from ambiguous outcrop in the east (Figure S3B). In the west, Okonguarri grainstone and conglomerate have a clear contact with arkosic arenite and siltstone of Narachaams Fm, which also contains monomict carbonate cobble conglomerate. Franni-aus Fm forms a westward tapering wedge of dolosiltite rhythmite and intraclast breccia with upward-increasing silicification. The uppermost Franni-aus Fm is composed of debrites and graded beds, and the basal Ghaub Fm contact is difficult to identify. Ghaub Fm is clearly identified from stable isotopes in Section A19 (Figure S6), however, which exhibit a stepwise increase in variability and the appearance of positive values indicating redeposition of Okonguarri/Narachaams Fms.

Franni-aus Fm thickens towards the fold and fault, while Okonguarri and Narachaams Fms thin down to just a few meters above a 100 m scale erratic of granite (Figure S3). This apparently conformable contact, along with dramatic thinning of units, may reflect paleotopography of the erratic. East of the erratic, however, breccias separate Narachaams Fm, which strikes into Chuos Fm and overturned Okonguarri Fm grainstone and conglomerate (Figure S3B). We interpret as do ref. (33) a fault relationship, unlike ref. (41), who interpret only a fold. In addition to the fault, a tight anticline folds Berg Aukas, Okonguarri, Ghaub, and Karibib Fms, which are all overturned along the strike of our inferred fault trace. It is difficult to distinguish Ghaub from Okonguarri Fm in this exposure, where Narachaams Fm also thins towards the inferred fault. Franni-aus Fm also effectively disappears across the structure from west to east. These abrupt transitions in unit thickness, as well as the observation that upper Ghaub Fm and Keilberg Mb seal the structure, imply that the faulting was active during Middle Cryogenian time.

East of the structure, we reinterpret much of the stratigraphy interpreted by ref. (33) and ref. (41) as Ghaub Fm to instead be Narachaams Fm. We map a surface with dozens of meters of relief on grainstones, oolites, and conglomerates of Okonguarri Fm, which ref. (41) attribute to erosion from channelized glacial flow. As we have described elsewhere across the ridge (Section S2), however, supra-Okonguarri Fm channelized surface(s) are demonstrably pre-Marinoan. Above the surface, we observe an interval dominated by monomict sedimentary breccia and conglomerate of dolostone (Figure S10C, E), which are isotopically heavy, consistent with sourcing from Okonguarri and Ombaatjie Fms (Figure S6). These channelized surfaces and redeposited facies are typical of deposition in a slope setting and not diagnostically glacial in origin. In addition to breccia and conglomerate, we observe an interval with green siltstone with arkosic and lithic (dolostone grain) arenite (Figure S10F). The conglomerate, siltstone, and arenite are indistinguishable from those in Narachaams Fm to the west of the structure, and the lithic arenite is particularly unique within the stratigraphy.

Nevertheless, we highlight a few horizons that contain outsized lonestones that might be interpreted as glacial dropstones (Figure S9). These few horizons (except Figure S9A) always occur within intervals of laminated dolosiltite interbedded with pebble-cobble dolostone conglomerate (Figure S10A, B, D). These conglomerates are lenticular and patchy over 10 m scales, consistent with deposition as small, locally sourced debris flows. The small scale and lateral discontinuity of these flows suggests that many of the lonestones are the edges of flows. For instance, Figure S9C shows a large cobble clast, but within a diffuse, unstratified horizon with several other granule–pebble clasts, which indicate simultaneous deposition. None of the lonestones show clear penetration of underlying laminations, although there is clear soft sedimentary deformation under the horizon in Figure S9C, which is also consistent with emplacement as a debris flow. These features are in contrast with the dropstone observed in Figure S10G, which shows unambiguous bed penetration and does not occur within a horizon containing diffuse stratification or other closely adjacent clasts.

While we cannot confidently ascribe a glacial origin for the lonestones described above, if we were to assume that they are in fact glacigenic, then we would interpret this interval as Ghaub Fm, as do refs (41) and (33). The channel mapped in Figure S3, which ref. (41) interpret as glacially carved, nevertheless remains ambiguous due to the presence elsewhere along Fransfontein Ridge of decameter-scale, pre-Marinoan channels on Okonguarri Fm (Figure S7). Under this interpretation, the only evidence for glaciation would be several dozen meters of rare ice-rafted debris prior to the first massive diamictites directly evidencing locally grounded ice. This sequence is potentially consistent with a calving ice front prior to Snowball onset. Grounded ice further up the slope could produce the gravity flow redeposition of Narachaams and Okonguarri Fms to explain the abundance of siltstones, arenites, and coarse dolostone conglomerates and sedimentary breccias in this interval.

Nevertheless, we cannot confidently report a traceable, unambiguous contact for basal Ghaub Fm in the central portion of this panel, where poorly sorted debrites and sedimentary breccias interfinger laterally and eventually begin to incorporate polymictic massive diamictites (Figure S10H) and stratified intervals with unambiguous dropstones (Figure S10G, Figure S22). In sections where we collected samples for stable isotope measurements, Ghaub Fm appears as a stepwise increase in the variability of δ^{13}C values (Sections A19, A20, A22 in Figure S6), which we interpret as mixing of eroded carbonates. This chemostratigraphic indicator coincides with the loss of siltstones and arenites and a transition to entirely carbonate (re)deposition. The contact that we map is our best estimate of this transition to glacial redeposition of platform and upper slope carbonates, based on the chemostratigraphy, the lowest confident dropstone-bearing intervals, and the continuity of outcrops of ambiguous redeposited carbonates.

The DW-1 tuff reported by ref. (33) occurs within the first unambiguously glacial stratified interval with dropstones, which itself sits above approximately 30 m of entirely carbonate clastic gravity flow deposits. We interpret this 30 m carbonate gravity flow interval as Ghaub Fm based on the abrupt transition in carbon isotopes from negative to positive (Section A22 in Figure S6). Below this 30 m interval, we interpret Franni-aus Fm on the basis of negative carbon isotopes within aclastic,

laminated dolosiltite immediately underlying the gravity flows. While this exposure of Franni-aus Fm is approximately along strike from the large olistolith of Franni-aus Fm discussed below, we do not observe any deformation. Instead, Franni-aus Fm appears to truncate against a plow structure associated with the olistolith to the east. The interval with ambiguous lonestones, mapped as brown and blue in Figure S3, underlies what we interpret as Franni-aus Fm. If this interval is in fact Ghaub Fm, then what we interpret as Franni-aus Fm is either a cryptic olistolith, or simply eroded and redeposited Franni-aus (due to its negative δ^{13}C). In either case, this sedimentary sequence would then beg the question of why glacial erosion would first erode and redeposit Okonguarri and Narachaams Fms, evidenced by siltstones, siliciclastics, and carbonates with positive δ^{13}C, before eroding and redepositing any of the stratigraphically overlying Franni-aus Fm.

Further east, the siltstone-bearing interval tapers along-strike into an approximately 570 m wide olistolith of Franni-aus Fm (Figure S3A). The base of the olistolith has cm-scale beds of rhythmically bedded dolosiltite, which coarsens into grainstone with varying degrees of silicification towards the top of the olistolith. The rhythmite and grainstone have negative to zero δ^{13}C values, which are only observed in Franni-aus Fm along the ridge (except basal Berg Aukas Fm). At both the eastern and western extents of the olistolith, adjacent stratigraphy is recumbently folded, including the same stratified interval that contains the DW-1 tuff. The dolosiltite rhythmites at the edges of the olistolith are pervasively deformed with soft-sedimentary folds (Figure S15). An interval of massive diamictite seals the olistolith and deformation of the underlying stratigraphy in its western exposure, where we define a thin Ghaub Fm. To the east, the olistolith is onlapped by stratified diamictite. Olistolith emplacement was syn-Marinoan, and we interpret the surrounding deformation to reflect plowing of the olistolith through the underlying stratigraphy. The occurrence of folds on either side of the olistolith indicates that its primary sense of displacement was along the dip direction, which is consistent with down-slope motion. The base of the olistolith has a sharp contact with green siltstones, which are also folded at the margins of the olistolith.

Finally, at the furthest east exposures, where we sampled AT22 FRAN 17 29 and MN1502 13, the interval of siltstone, arenite, and abundant carbonate breccia and conglomerate tapers out, and Ghaub Fm rests on unambiguous Okonguarri Fm. The contact with Okonguarri Fm is erosional, with brecciation of the uppermost Okonguarri Fm. Basal Ghaub Fm consists of massive diamictite directly above a sharp contact, which we interpret as a lodgement till deposited under grounded ice. This sequence is distinct from the conformable contact between Franni-aus and Ghaub Fms further west (Section S2.A), where basal Ghaub Fm consists of pro-grounding line facies. We interpret that the initial snowball ice advance introduced hiatus at this location, although the ages from AT22 FRAN 17 29 and MN1502 13 indicate that this hiatus was only on the order of a few hundred thousand years, at least with respect to glaciomarine accumulation along western exposures.

S3. Geochronology

Table S2 shows all samples collected for geochronology, which are described individually below. Tuffaceous samples discussed in the manuscript are described first (Section S3.B), followed by the detrital samples (Section S3.C)

A. Methods. Rock samples were sledged and shatter boxed, after which a 500 µm size grain fraction was isolated via sieving. These separates were manually rinsed and panned, and individual zircon crystals were picked from the densest separates remaining after aggressive panning. We did not utilize magnetic or heavy liquid separation methods. All samples except AT22 FRAN GC 06 were annealed at 900°C for 60 hours in a muffle furnace prior to analysis via laser ablation split stream (LASS) inductively coupled plasma mass spectrometry ICPMS; all analyses (including from AT22 FRAN GC 06) were annealed prior to analysis via chemical abrasion isotope dilution thermal ionization mass spectrometry (CA-ID-TIMS).

We analyzed zircon geochemistry first via LASS-ICPMS, followed by CA-ID-TIMS for promising grains identified in the first step.

A.1. LASS-ICPMS. Zircon crystals were mounted in rows on sticky tape on a glass mount, and epoxy was poured to make a mount with zircon grains embedded. Mounts were polished to expose the zircon crystal interiors. All polished mounts were imaged via a cathodoluminescence (CL) detector on a FEI Quanta 400f field-emission scanning electron microscope at UCSB. The CL imagery was used to place spots for laser ablation; in general, we targeted the rims of grains to ascertain the youngest age. The LASS-ICPMS procedure was performed as described by ref. (45). The laser instrument was a Photon Machines Analyte 193 with an ATLEX-SI 193 nm ArF excimer laser. Spot sizes on the zircon crystals were either 20 or 25 µm. Measurements of U and Pb were collected either on a Nu Instruments Plasma 3D or HR-ES multi-collector instrument, while trace elements were measured on an Agilent 7700x quadrupole instrument. Full isotopic data is provided in the supplementary material.

A.2. CA-ID-TIMS. Potential volcanic ash samples with unimodal, young zircon crystal populations were identified via LASS-ICPMS. We plucked individual zircon crystals from the epoxy mounts, targeting grains without visible inclusions or inclusions identified as spikes in mass 204, P, or Ti from the LASS-ICPMS data. Additional criteria included targeting the largest, most euhedral grains possible.

Zircon grains were rinsed in HNO_3 and then loaded into 300 µl Teflon PFA microcapsules with 29 M HF for chemical abrasion (CA). Zircon grains underwent CA at either 180 or 190°C for 12 hours; the temperature for each analysis is shown in Table S1. After CA, zircon grains were rinsed in H_2O, sonicated in 3.5 M HNO_3 for 30 minutes, and finally fluxed on a hot plate at 80°C for one to two hours. The HNO_3 was removed and zircon was rinsed twice in ultrapure H_2O before being reloaded into the 300 µl Teflon PFA microcapsules (rinsed and fluxed in 6 M HCl during sonication and washing of the zircon) and spiked with the EARTHTIME mixed ^{233}U-^{235}U-^{202}Pb-^{205}Pb tracer solution (ET2535). Zircon was dissolved in Parr vessels in 120 µl of 29 M HF at 220°C for 48 hours, dried to fluorides, and re-dissolved in 6 M HCl at 180°C overnight. U and Pb were

separated from the zircon matrix using an HCl-based anion-exchange chromatographic procedure (46), eluted together and dried with 2 µl of 0.05 N H_3PO_4.

Pb and U were loaded on a single outgassed Re filament in 5 µl of a silica-gel/phosphoric acid mixture (47), and U and Pb isotopic measurements made on a GV Isoprobe-T multicollector thermal ionization mass spectrometer equipped with an ion-counting Daly detector. Pb isotopes were measured by peak-jumping all isotopes on the Daly detector for 220 cycles. Analyses were corrected for mass fractionation using the known ^{202}Pb/^{205}Pb ratio of the tracer solution. Transitory isobaric interferences due to high-molecular weight organics, particularly on ^{204}Pb and ^{207}Pb, disappeared within approximately 60 cycles, while ionization efficiency averaged 104 cps/pg of each Pb isotope. Linearity (to $\geq 1.4 \times 106$ cps) and the associated deadtime correction of the Daly detector were monitored by repeated analyses of NBS982, and have been constant since installation. Uranium was analyzed as UO_2^+ ions in static Faraday mode on 1012 ohm resistors for 300 cycles, and corrected for isobaric interference of ^{233}U^{18}O^{16}O on ^{235}U^{16}O^{16}O with an ^{18}O/^{16}O of 0.00206. U mass fractionation was corrected using the known ^{233}U/^{235}U ratio of the tracer solution.

U-Pb dates and uncertainties were calculated using the algorithms of ref. (48), calibration of ET2535 tracer solution (49) of ^{235}U/^{205}Pb = 100.233, ^{233}U/^{235}U = 0.99506, ^{205}Pb/^{204}Pb = 8474, and ^{202}Pb/^{205}Pb = 0.99924, U decay constants recommended by ref. (50), and ^{238}U/^{235}U of 137.818 (51). ^{206}Pb/^{238}U ratios and dates were corrected for initial ^{230}Th disequilibrium using DTh/U = 0.20 ± 0.05 (1σ) and the algorithms of ref. (52), resulting in an increase in the ^{206}Pb/^{238}U dates of ≈0.09 Ma. All common Pb in analyses was attributed to laboratory blank and subtracted based on the measured laboratory Pb isotopic composition and associated uncertainty. U blanks are estimated at 0.013 pg.

Weighted mean ^{206}Pb/^{238}U dates are calculated from equivalent dates (probability of fit >0.05) using Isoplot 3.0 (53) with error at the 95% confidence interval. Error is computed as the internal standard deviation multiplied by the Student's t-distribution multiplier for a two-tailed 95% critical interval and n-1 degrees of freedom when the reduced chi-squared statistic, mean squared weighted deviation (MSWD) (54), takes a value less than its expectation value plus its standard deviation at the same confidence interval, which is when MSWD is $< 1 + 2\sqrt{2/(n-1)}$. This error is expanded via multiplication by the $\sqrt{MSWD}$ when the MSWD is $\geq 1 + 2\sqrt{2/(n-1)}$ to accommodate unknown sources of over dispersion. Errors on the weighted mean dates are given as ± x / y / z, where x is the internal error based on analytical uncertainties only, including counting statistics, subtraction of tracer solution, and blank and initial common Pb subtraction, y includes the tracer calibration uncertainty propagated in quadrature, and z includes the ^{238}U decay constant uncertainty propagated in quadrature. Internal errors should be considered when comparing our dates with ^{206}Pb/^{238}U dates from other laboratories that used the same tracer solution or a tracer solution that was cross-calibrated using EARTHTIME gravimetric standards. Errors including the uncertainty in the tracer calibration should be considered when comparing our dates with those derived from other geochronological methods using the U-Pb decay scheme (e.g., laser ablation ICPMS). Errors including uncertainties in the tracer calibration and ^{238}U decay constant (50) should be considered when comparing our dates with those derived from other decay schemes (e.g., ^{40}Ar/^{39}Ar, ^{187}Re-^{187}Os). Errors on dates from individual analyses are 2σ.

B. Tuffaceous Samples.

B.1. AT22 FRAN GC 06. AT22 FRAN GC 06 was sampled from basal Ghaub Fm 3.8 m above a conformable contact (Figure S13B) with Franni-aus Fm (Figure S21). The sample is an orange-weathering green siltstone traceable over 50 m locally before encountering cover, but it might be present over 4 km kilometers further east (main manuscript Figure 2D). It occurs within a well stratified interval of cm-scale dolosiltite and doloarenite with abundant dropstones. Above the sampled horizon, the stratigraphy coarsens as clast density increases and debrites increase in frequency, eventually culminating in massive diamictite with a sharp basal contact and soft sedimentary deformation of underlying stratified diamictite. We interpret this sequence to record the initial advance of the grounded Marinoan ice sheet.

Sixty zircon grains were separated from this sample and dated via LASS-ICPMS (Figure S24A). A handful of Paleoproterozoic ages are consistent with reworking of zircon from the immediately adjacent Kamanjab Inlier (Section S3.C). Of the young zircon, eleven of the largest and most euhedral grains lacking inclusions were selected for CA-ID-TIMS geochronology. The resulting ^{206}Pb/^{238}U ages form a single population of 638.93±0.32 Ma with an MSWD of 0.8. We interpret the weighted mean age to be a reasonable estimate of the eruptive age of the sample, which we interpret to be a reworked volcanic ash. We conclude that this age is likely within 10-100 ky of the depositional age as per the discussion in the main manuscript.

B.2. AT21 FRAN 24. AT21 FRAN 24 is a resampling of DW-1 reported by ref. (33) (Figure S22). We are confident that we identified the exact sampling locality given that there was still evidence of disturbed outcrop when we sampled. We also received in person confirmation at the sampling locality directly from the lead author. The sample is a green siltstone that outcrops in discontinuous pods, at most 20 cm thick, that are nevertheless traceable over 500 m (Figure S3). Like AT22 FRAN GC 06, this sample occurs within a stratified interval of Ghaub Fm consisting of interbedded dropstone-bearing laminated dolosiltite and 0.5-1 m cobble to sand dolostone graded beds. Multiple siltstone horizons with similar outcropping style occur within the stratified dropstone-bearing interval. The stratified interval is truncated to the east by a kilometer-scale olistolith of Franni-aus Fm. The stratified interval is folded and overturned against the folded western margin of the olistolith (Figure S3), which we interpret as a plow structure associated with syn-Marinoan olistolith emplacement.

The relationship of this sample with respect to the basal Ghaub Fm contact is ambiguous (Section S2.B), but we interpret a significantly thinner Ghaub Fm than ref. (33) and ref. (41). We do not observe a clear stratigraphic record of an initial ice advance, as we do further west. Given the absent Franni-aus Fm and multiple erosional surfaces throughout the panel, as well

as the clearly erosional basal Ghaub Fm surface a kilometer east, we conclude that it is unlikely that the basal Ghaub Fm at this locality records Marinoan onset.

We separated 91 zircon grains from this sample to date via LASS-ICPMS. Like with AT22 FRAN GC 06, the few Paleoproterozoic zircon likely reflect incorporation of local basement material in the siltstone. Thirteen euhedral grains were analyzed via CA-ID-TIMS (Figure S22). Of these, the $^{206}Pb/^{238}U$ages of two analyses are too old to belong to the young population constituted by the other 11 analyses, which yield a weighted mean age of 638.23±0.11 Ma with an MSWD of 1.0. As discussed in the manuscript, the weighted mean age of the younger 11 grains is ca 1 My younger than the age reported by ref. (33). The DW-1 age has an MSWD of 2.6, indicating dispersion beyond analytical uncertainty. Furthermore, ref. (33) reported one grain with an age of 637.5±0.41 Ma, which they do not include in their weighted mean age. They interpret this grain to have experienced early lead loss, but it nearly overlaps with our weighted mean age.

Our analyses also exhibit dispersion, with the oldest two grains overlapping with the DW-1 weighted mean age. We conclude that this sample exhibits geologic dispersion in zircon ages that ref. (33) were unable to resolve with their sample set, with the further confounding factor that the analyses were performed in different labs. Nevertheless, we have identified a population of 11 grains that confidently establishes an age ca. 700 kyr younger than GC06. We have identified a young magmatic population represented by our weighted mean age, but we acknowledge the possibility that the sample could be younger. Regardless, the dated horizon is likely younger by at least 1 Myr than the date reported by ref. (33).

The CA-ID-TIMS analyses of ref. (33) yielded more radiogenic lead than ours (Table S1), indicating that they analyzed a larger size fraction of zircon. It is possible that grain size bias could explain the dispersion we observe between the sample sets, whereby the smaller grains that we dated were younger. Finally, we note that the zircon trace element geochemistry of AT21 FRAN 24, barring a single outlier, is unimodal and distinct from AT22 FRAN GC 06 but overlapping with AT22 FRAN 17 29 and MN1502 13.

B.3. AT22 FRAN 17 29. This sample is a yellow-green siltstone (Figure S23) originating from a subcropping, up to 10 m thick package within Ghaub Fm in the easternmost panel of our study area (Figure S3). We sampled near the base of this unit, at 40 m in composite section A26 (29 m in AT22 FRAN 17). The siltstone exhibits a weak bedding parallel foliation and lacks grain size variability and diagnostic bedforms. Ghaub Fm in this location rests on an erosional contact with oolite and conglomerate of Okonguarri Fm. Basal Ghaub Fm is a massive diamictite, followed by interbedded laminated dolosiltite and cobble conglomerate, with dropstones present in the dolosiltite in the upper 2 m of the unit (Figure S23). The 10 m siltstone follows the stratified dropstone-bearing interval. We observed two other cm-scale siltstone horizons within the dropstone-bearing interval.

From the sequence stratigraphy, we propose that the initial Marinoan ice advance surpassed this location, eroded Okonguarri Fm and deposited the massive diamictite as a lodgement till. The subsequent stratified interval records grounding line retreat and pro-grounding line accumulation, which is where we observe the thick siltstone that we sampled. This sample postdates onset.

We separated 201 zircon grains from this sample to date via LASS-ICPMS. Nearly every zircon grain yielded a ca. 640 Ma Neoproterozoic date, with a handful of Paleoproterozoic grains likely recording reworking, as discussed for the previous samples (Figure S24D). Eight euhedral grains all yielded concordant analyses via CA-ID-TIMS (Figure S23), and the resulting $^{206}Pb/^{238}U$ages form a single age population with a weighted mean age of 638.45±0.10 Ma and an MSWD of 0.9. We interpret the weighted mean age to be a reasonable estimate for the eruptive age of the sample.

The zircon geochemistry of AT22 FRAN 17 29 forms a unimodal, clustered population that overlaps with MN1502 13 and AT21 FRAN 24. The unimodal behavior is consistent with sourcing from a single magmatic environment and supports our interpretation of a volcanic origin for the sample.

B.4. MN1502 13. This sample was interpreted in the field to come from the same unit as AT22 FRAN 17 29, which is approximately 200 m to the east. Direct tracing of of the unit was not possible, however, due to poor outcrop. MN1502 13 is a subcropping green, massive claystone (Figure S23). The depositional context for this sample is similar to AT22 FRAN 17 29, except that a westward expanding wedge of carbonate conglomerate and sedimentary breccia complicates precise assignment of the basal Ghaub Fm contact (Figure S3).

25 zircon grains from this sample had previously been analyzed via LASS-ICPMS, and all grains returned ages ca. 640 Ma. We analyzed four grains via CA-ID-TIMS yielding a weighted mean age of 638.66±0.40 Ma with an MSWD of 1.1 (Figure S23), which overlaps with the weighted mean age for AT22 FRAN 17 29. The zircon trace element geochemistry of MN1502 13 also closely overlaps with that of AT22 FRAN 17 29, further supporting the interpretation that they are the same unit.

C. Detrital Zircon Geochronology. In addition to the tuffaceous siltstones reported in the main manuscript, we sampled several other horizons within the Ghaub and Franni-aus Fms for detrital zircon geochronology. These samples are briefly discussed below. Other than incorporation of Damaran zircon via sampling contamination, these detrital samples contain no young zircon that would provide useful maximum depositional age constraints. All samples were dated via LA-ICPMS U-Pb geochronology on zircon (Table S2), and for some samples, young grains identified via LA-ICPMS were analyzed via CA-ID-TIMS. In all cases, concordant young analyses were most consistent with sampling contamination of young Damaran age zircon.

All isotopic data are provided in the accompanying supplemental spreadsheet files, which also include sample and analysis metadata. Table S2 summarizes our geochronology samples with coordinates and stratigraphic context.

C.1. AT21 FRAN 26. This sample comes from 16.2 m in section AT21 FRAN 14 (upper half of composite section A14). It occurs within a stratified interval containing IRD and several tuffaceous siltstone beds previously reported by ref. (41). The sample is one of these siltstone beds, which we interpret to be within 2 m of the base of the Ghaub Fm (Figure S25).

We analyzed 223 zircon grains via LASS-ICPMS, and the resulting zircon ages were predominantly Paleoproterozoic, with a minor Mesoproterozoic range and a handful of younger Neoproterozoic to Cambrian grains. The concordant 550–500 Ma grains likely indicate sampling contamination, as they correspond to ages syn- and post-tectonic Damaran granites that are abundant further south (55).

Five zircon grains in the 600–650 Ma range were analyzed via CA-ID-TIMS. One young grain yielded an age of 648.79±0.25 (Table S1), but three other grains were concordantly Ediacaran, which is too young. These grains may have been introduced via sampling contamination, and we do not interpret them further with respect to the age of deposition of the sample.

C.2. AT22 FRAN 16 1.3. This sample is a poorly outcropping fissile green siltstone at the base of Narachaams Fm (Figure S26). The approximately 2 m package of siltstone rests above a conglomerate of Okonguarri dolomite grainstone and coarsens into a medium sandstone. This unit marks the onset of alternation between siltstone, arenite, and dolomite conglomerate. Ref. (41) interpret this horizon as basal Ghaub Fm, whereas we prefer to interpret it as basal Narachaams Fm.

The dominant age peaks are 1.9 and 2.0 Ga, with fewer grains also in the interval 1.2–1.6 Ga. A single Archaean grain is present, and there is also a single young concordant zircon that could potentially provide a maximum depositional age constraint. The LA-ICPMS $^{206}Pb/^{238}U$ age for this grain is 655±13.4 Ma. This grain has not yet been analyzed via CA-ID-TIMS.

C.3. AT22 FRAN 16 62. This sample is a pod of green siltstone, up to 30 cm thick, laterally truncated by overlying dolomite conglomerate. We interpret the unit to be Franni-aus Fm, which is supported by relatively low variability carbon isotopes around 0‰ (Figure S6). The siltstone is similar in appearance to the underlying Narachaams Fm siltstone.

We separated 51 zircon grains from the sample for analysis via LASS-ICPMS. Most ages were Paleoproterozoic, with a few Archaean and Mesoproterozoic analyses.

C.4. AT22 FRAN 15 7.7. AT22 FRAN 15 7.7 is a green siltstone above 8 m of Franni-aus Fm dolosiltite. The siltstone and dolosiltite are laterally truncated by massive diamictite of Ghaub Fm, which we interpret as erosion resulting from the initial ice advance. The siltstone is similar in outcrop character to the underlying Narachaams Fm siltstone.

We analyzed 85 zircon grains via LASS-ICPMS, and most analyses were Paleoproterozoic with a handful of Mesoproterozoic grains. One grain was of Damaran age, which we attribute to sampling contamination.

C.5. AT22 FRAN 19 7.7. This sample is a thin (<5 cm)recessive shaley siltstone parting between two tabular intraclast breccia beds within Franni-aus Fm dominated by dolosiltite rhythmite. We analyzed 235 zircon grains via LASS-ICPMS. The vast majority of analyses were Paleoproterozoic, and we also obtained excessively young Damaran grains that we attribute to sampling contamination due to the difficulty of sampling the fissile material.

A handful of Neoproterozoic grains seemed promising enough to analyze via CA-ID-TIMS, but they all yielded concordant Ediacaran and Cambrian ages (Table S1), which we attribute to sampling contamination.

C.6. AT22 FRAN 21 90.5. AT22 FRAN 21 90.5 is a green arkosic arenite (Figure S29) that rests on a surface above Okonguarri Fm (Figure S3), which ref. (41) attributes to channelized glacial erosion. Given the preceding discussion about previously undescribed and demonstrably pre-Marinoan channelized surfaces on Okonguarri Fm (Section S2), as well as the paucity of unambiguously glacial sediments in the subsequent stratigraphy (Section S2.B), we instead reinterpret much of the stratigraphy above the contact as Narachaams Fm, including the basal arenite.

We analyzed 208 zircons via LASS-ICPMS, obtaining two main age peaks in the detrital age spectrum: a narrower Paleoproterozoic peak and wider Mesoproterozoic peak. Several Archaean ages are also present in the dataset. A single Neoproterozoic analysis yields an age of 730±16 Ma.

C.7. AT21 FRAN 20. This sample is a coarse granule-cobble conglomerate in pervasively silicified uppermost Franni-aus Fm (Figure S30) in the westernmost panel of our study area (Figure S4). The conglomerate clasts consist of partially silicified oolite and dolosiltite. We analyzed 119 zircon grains via LASS-ICPMS, resulting in primarily Paleoproterozoic ages. We interpret a few Ediacaran ages as sampling contamination.

C.8. Detrital Provenance. The detrital age spectra for all samples are dominated by Paleoproterozoic peaks at 1.8 and 1.9 Ga, which most likely represent zircon sourced by the immediately underlying Orosirian basement (56). The smaller and diffuse peak of ages from 1.1–1.5 Ga is readily explained by longlived 1.1–1.6 Ga magmatism associated with the Kunene Complex in northern Namibia and southern Angola (57, 58).

Variability in the presence of 1.0–1.2 Ga vs 1.3–1.5 Ga zircon between samples, cf. AT21 FRAN 26 and AT22 FRAN 21 90.5, suggests that the specific magmatic sources for the 1.0–1.2 Ga grains are eroded independently of the older sources that characterize the bulk of the Kunene Complex. This observation is consistent with the speculation from ref. (57) that late Mesoproterozoic magmatism extended south of the Kunene Complex into southern Kaokoland, thereby potentially providing erosional sources independent of the Kunene Complex. Rarer Archaean grains are consistent with erosion from the Congo craton in the north (59).

S4. Subsidence Modeling

As pointed out by ref. (41, 60), the average 380 m thickness of the single transgressive-regressive sequence recorded in the post-Marinoan Maieberg Fm reflects rapid sedimentation into accommodation that must have generated during a several million-year glaciation. The required tectonic subsidence is approximately 90 m on the Otavi Platform (60), where little (on average 30 m) glacial erosion occurred (61). Ref. (60) estimate a duration of ca. 9 Myr for the Marinoan based on subsidence modeling given uncertainty chronology and assumptions on the cessation of rifting. Ref. (39) also conducted subsidence modeling and constrained a range of reasonable crustal stretching factors from 1.15–1.25.

We use the thermal subsidence model of ref. (62) to estimate thermal subsidence over the duration of the Marinoan Snowball glaciation for two models: one adhering to our interpretation of onset ca. 639 Ma (4 Myr duration) (Figure S31A) and one for a 15 Myr duration with onset ca. 650 Ma (Figure S31B). The first model nicely yields the required 90 m of tectonic subsidence for $\beta \leq 1.2$ with rift cessation ca. 660–655 Ma, which also matches the preferred rift-to-drift transition ca. 657–655 Ma of ref. (41). A 15 My Marinoan snowball generates at least 300 m of tectonic subsidence for the considered parameters, which is too much. Thus, our chronology fits neatly with the subsidence required to account for the thickness of the cap dolostone sequence recorded in the Maieberg Fm.

S5. Ash Delivery Models

Several potential mechanisms exist for depositing ashes in the sub-ice setting (Figure S34), each making distinct predictions for the distributions of zircon ages and geochemistry within and between sampled horizons.

The first mechanism we consider is entrainment of ash on the accummulation zone of a terrestrial Snowball ice sheet (Figure S34A). Ash would be quickly covered in snow, minimizing the likelihood of mixing with other volcanic or detrital soures. The ash would then flow through the ice sheet, traversing it as a coherent band (63, 64). At the downstream reaches of the ice sheet, some part of the ash horizon might intersect the base of the ice sheet in the glaciomarine setting, where it undergoes basal melting. The ash would release from the ice sheet to settle through the water column. The geometry of the ash horizon's intersection with the underside of the ice shelf would depend on its flow through the ice sheet, meaning that it might melt out of the ice sheet diachronously along strike, potentially limiting its lateral traceability as a bed. The ash would also be subjected to reworking by currents, as would a typical airfall ash. This model predicts that a single tuffaceous deposit would incorporate zircon crystals from a single eruption, meaning that the crystals should constitute a single age and geochemical population.

Another mechanism for depositing syn-Snowball volcanic ash is sub-marine volcanism beneath the Snowball sea glacier (Figure S34B). Unlike explosive sub-aerial eruptions, the primary volcanic ash deposits of a explosive sub-marine eruption are not far traveled, typically reaching only a few dozen kilometers from the source (65–69). Ref. (67) suggest that eruption-fed density currents carrying volcanic ash can potential travel on the order of a hundred kilometers, based on the modeling and observation of far traveled fine-grained turbidites (70). Sub-marine volcanism can also generate pumice rafts that can travel hundreds to thousands of kilometers (71, 72) and cover hundreds of thousands of square kilometers (72). These pumice rafts generate sedimentary ash deposits by abbrading against each other during buoyant transport; these deposits are predicted to contain outsized pumice clasts that sink after incorporating sufficient water (72). It is not clear how pumice rafting would operate underneath a sea glacier, which might hinder dispersal in comparison with an ice-free marine surface. Both styles of submarine volcanic deposition discussed above would predict single age and geochemical populations for zircon crystals within the deposits.

A third mechanism, proposed by ref. (73), is the flushing of cryoconite and dust from the surface of the sea glacier at low latitudes (Figure S34C). The sea glacier at low latitudes is a place of net ablation, where ice flows up from below to replace what is lost at the surface (74). This conveyor belt moves dust entrained in the meteoric part of the sea glacier at high latitudes to low latitudes (75), where it accumulates on the surface. The dark dust absorbs incoming solar radiation, melting the ice to form meltwater ponds on the relatively flat surface of the sea glacier. In the model of ref. (73), these pools provide refugia for photosynthetic organisms, which contribute organic matter to the mix. Eventually, the pools flush via moulins through the sea glacier, delivering the mixture of dust and organic matter to the glaciomarine setting. This model predicts heterogeneous zircon age and geochemical populations within a single sample, which would mix a variety of detrital sources during potentially long-lived residence in the sea glacier accumulation zone.

Finally, we consider the mechanism of direct airfall deposition and settling through the water column (Figure S34D). Ref. (76) model the growth of marine-terminating, low-latitude glaciers before the ice-albedo runaway into the Snowball; however, their modeling assumed nearby mountains, and this result is inconsistently reproducible. For instance, ref. (77) do model sub-tropical marine-terminating ice prior to Snowball collapse, but again in association with proximal mountains. Assuming that a marine-terminating glacier did exist on the Otavi Platform prior to Snowball runaway, then airfall deposition is a viable mechanism for ashes observed within the earliest pro-grounding line glacial sediments.

Alternatively, it may be possible for open water to exist during a Snowball in certain paleogeographic configurations. Several modelers have explored the possibility of thin ice in narrow seaways, such as the modern Red Sea (74, 78, 79). Constrictions cause the sea glacier to thin as it flows in to fill them; sufficiently narrow seaways are then capable of totally exhausting the sea glacier. Given the potential proximity of the Swakop Terrane to southern Congo in Marinoan time (80), it is possible that the hypothetical Outjo Sea separating them was quite narrow. In conjunction with a potentially proximal arc impinging from the west (81, 82), the marine setting around southwestern Congo may have been constricted, shielding it from the thick sea glacier and permitting early airfall ash accumulation.

Within each sample we analyzed, we observe unimodal age and geochemical populations (Figures S20 & S24), indicating that each horizon captures a single eruption. This observation precludes prolonged exposure on the ablation zone of the ice surface, which would mix volcanic and detrital zircon sources, and we therefore reject the cryoconite flushing mechanism (Figure S34C). There are no known Cryogenian volcanic sources along southern Congo, and we dismiss a proximal local source for volcanic material. Additionally, we have not observed any pumice clasts within the ash horizons, nor is it clear that pumice rafting would be an effective transport mechanism during a Snowball glaciation. Finally, sample GC06 exhibits a mantle zircon trace element geochemistry distinct from the other samples (Figure S20), potentially indicating a distinct magmatic source. This result is also challenging to reconcile with a proximal submarine source for all of the ashes, although it is conceivable that multiple delivery mechanisms are operating simultaneously. We dismiss a submarine volcanic source for the ashes we observe.

The unimodal age and geochmical populations for the ashes are consistent with the entrainment and airfall models, which we discuss in the main manuscript.

S6. Drone Workflow

Droning proceeded in two stages: the first pass collected 1.5–2 cm/px imagery with which to generate a high resolution elevation model, followed by a second pass over specific targets to collect 5 mm/px imagery. All imagery was captured by a DJI Mavic Pro 2 quadcopter drone. The first passes collected 9729 images across a 16 km along-strike exposure of the Ghaub Formation along Fransfontein Ridge (Figure S36). An approximately 700 m by 280 m panel of well-exposed outcrop immediately east of the C35 road was targeted for a second pass of high-resolution imaging, which gathered 6134 images at 5 mm/px resolution.

First pass flights were planned in the UgCS software by SPH Engineering, which permits offline drone mission planning. The UgCS Photogrammetry area flight planning tool was used to automatically generate missions to gather imagery. We defined polygons bounding an area of interest and subjectively defined a flight direction to be parallel to the smoothest direction of topography (i.e., parallel with ridges). Flight lines were parallel to this direction, and the software automatically arranged them to achieve a horizontal overlap of 50% between images from adjacent flight lines. Images within a flight line were captured with 60% vertical overlap. The UgCS software was able to cache and use 1 arc-second (30 m) elevation data from the shuttle radar tomography mission (SRTM) to ensure approximately consistent flight elevation above the surface and thereby maintain a target ground sampling distance (GSD) of 1.5–2 cm/px, corresponding to above ground elevations of 60–70 m. The drone speed was set at 5 m/s, which is the fastest speed for which the target overlap of 60% could be achieved while maintaining time between images to at least 2 seconds (the DJI Mavic 2 Pro cannot capture images more quickly than one every two seconds). Three approximately 20–25 minute flights were undertaken between 11:00am–2:00pm on any given day, which ensured minimal shadows and consistent illumination between flight days.

Prior to flying a day's missions, ground control points (GCPs) were constructed with a typical spacing of 150–300 m. We arranged cobble-sized rocks into approximately 1–1.5 m squares, with a high-contrast cobble placed in the center of the square (Figure S37). These proved to be easily identifiable in the imagery. The high-contrast cobble was surveyed as the GCP using a BadElf GNSS Surveyor. Each GCP was surveyed for 5–10 minutes. While these points could be post-processed via differential correction with contemporaneous observations from a nearby reference station, we did not have our own base station, and we have not found data sources for Namibian reference stations. We used the mean and standard deviations of the original position data as measures of the GCP position and uncertainty. Typical horizontal position uncertainties were 0.7–3.5 m, and typical vertical position uncertainties were 1.1–6.4 m (Figure S38). These uncertainties are much greater than the cm-scale uncertainty associated with identifying the surveyed location of the GCP cobble in the imagery.

After a day's missions, the collected imagery was processed in the field with Agisoft Metashape. The processing steps undertaken were image alignment and dense point cloud construction, both at low quality settings. With 800–1000 images collected daily, it was possible to perform these processing steps within an hour or two on a laptop to evaluate whether image quality and coverage was sufficient to completely reconstruct the target area via photogrammetry. In the rare cases when certain areas were not sufficiently well-imaged (typically over steep terrain), extra imagery was captured in subsequent flights.

Second pass 5 mm/px flights were planned in Agisoft Metashape using the Plan Mission tool. This tool accepts a target GSD and overlap between images, and it uses the topographic surface to optimize camera locations and orientations to achieve the desired GSD and overlap. Whereas the missions planned in UgCS always have the camera facing down (nadir imagery), the mission planning in Metashape strives to keep the topographic surface parallel to the focal plane, optimally orienting the drone camera to do so. This procedure is much more effective for resolving steep and complex surfaces that often exhibit the best geologic outcropping. This mission planning, however, required a meshed representation of the topographic surface generated by Metashape, which was constructed from the first pass of lower resolution flights. These meshes provide accurate and well-resolved elevation models that ensure that the drone does not collide with the surface when flying 20–25 m from topography.

S7. Compensation Length Scale Estimation

We utilized the 5 mm/px drone imagery to estimate the compensation length scale (CLS) of glaciomarine sedimentation and grounding line cycles in Ghaub Fm. Because bedding attitudes are approximately constant along the relatively small high-resolution panel, thicknesses between bedding contacts can be estimated from a two-dimensional image of outcrop perpendicular to bedding. In Agisoft Metashape, it is possible to project imagery onto any arbitrary plane. We projected the 5 mm/px imagery onto the plane perpendicular to bedding, yielding a mosaic of the outcrop with younging direction

being vertical. The resultant mosaic is associated with a coordinate that properly encodes distances and can be exported as a GeoTIFF.

Bedding contacts were traced onto the GeoTIFF in QGIS. We digitized any contact that was confidently traceable over at least several meters; longer contacts are better for robust evaluation of σ_{ss}. All types of contacts were traced; the compensation length scale is a measure of the stacking geometry of sediments, which is sensitive to both erosional and aggradational contacts. In total, 404 contacts were traced. Points were sampled at roughly 0.1–1 m spacing along contact traces, and 95% of traces had 8 points or more, with a median of 19 points per trace.

The stratigraphic thickness of the Ghaub Fm was decompacted using the backstrip_well function in pybacktrack (83). We assumed a thickness of 500 m for the Karibib Fm on the slope sections (41) and a thickness of 3380 m for the Mulden Gp, as estimated from seismic stratigraphy in the Owambo Basin (84). The decompaction correction accounted for lithology-specific compaction factors, and the relative abundances of lithologies by formation in the Owambo Basin are reported by ref. (84). This approach likely overestimates actual compaction of the Ghaub Fm on the slope by utilizing a Mulden Gp thickness from its thickest foredeep deposits, whereas burial of the Ghaub Fm in the foreslope zone (sensu (41)) would likely have been less. We obtain a compaction factor of 1.47 for Ghaub Fm, which we multiply with the y-coordinates of our bedding contact traces described above. Our decompacted Ghaub Fm thickness provides an upper bound on true thickness and thereby on the compensation length scale.

Thicknesses were then evaluated between overlapping portions of pairs of traced contacts. At least eight points had to overlap between traces for consideration. The distance between traces was evaluated as the vertical (i.e., decompacted up-section) distance from each point on the trace to the line of the paired trace. The quantity σ_{ss} (Equation 1, main text) was then evaluated for all distances between the pair of traces, along with the average thickness L separating the traces. The resulting σ_{ss} values for the 3442 pairs of contacts are scattered, so they were binned in logarithmic bins of L and geometrically averaged.

We then utilized PyMC 5 (85) to generate a Bayesian estimate for the transition in power law behavior. We modeled the transition D as a deterministic variable set by the intersection of two linear fits in log-log space: one to the non-compensational ($L < D$) regime, with variable slope, and one to the compensational ($L > D$) regime, with slope fixed at -1. The three stochastic variables in this model are just the intercepts of both linear fits and the slope of the non-compensational term. The likelihood term involved the geometric standard error of σ_{ss} within 19 bins of L. The posterior kernel density estimate for D is based an effective sample size of roughly 20,000 from 80,000 samples. The Bayesian model is provided in the supplied code. The posterior distribution for the non-compensational term is shown in Figure S33.

S8. Congo Paleolatitude

Ref. (86) report a subtropical paleolatitude of 33.2±3.2° for southern Congo. These data, however, fail a fold test, and we interpret this pole as a late Ediacaran overprint. The paleolatitude of the Congo from ca. 639–635 Ma therefore remains poorly constrained.

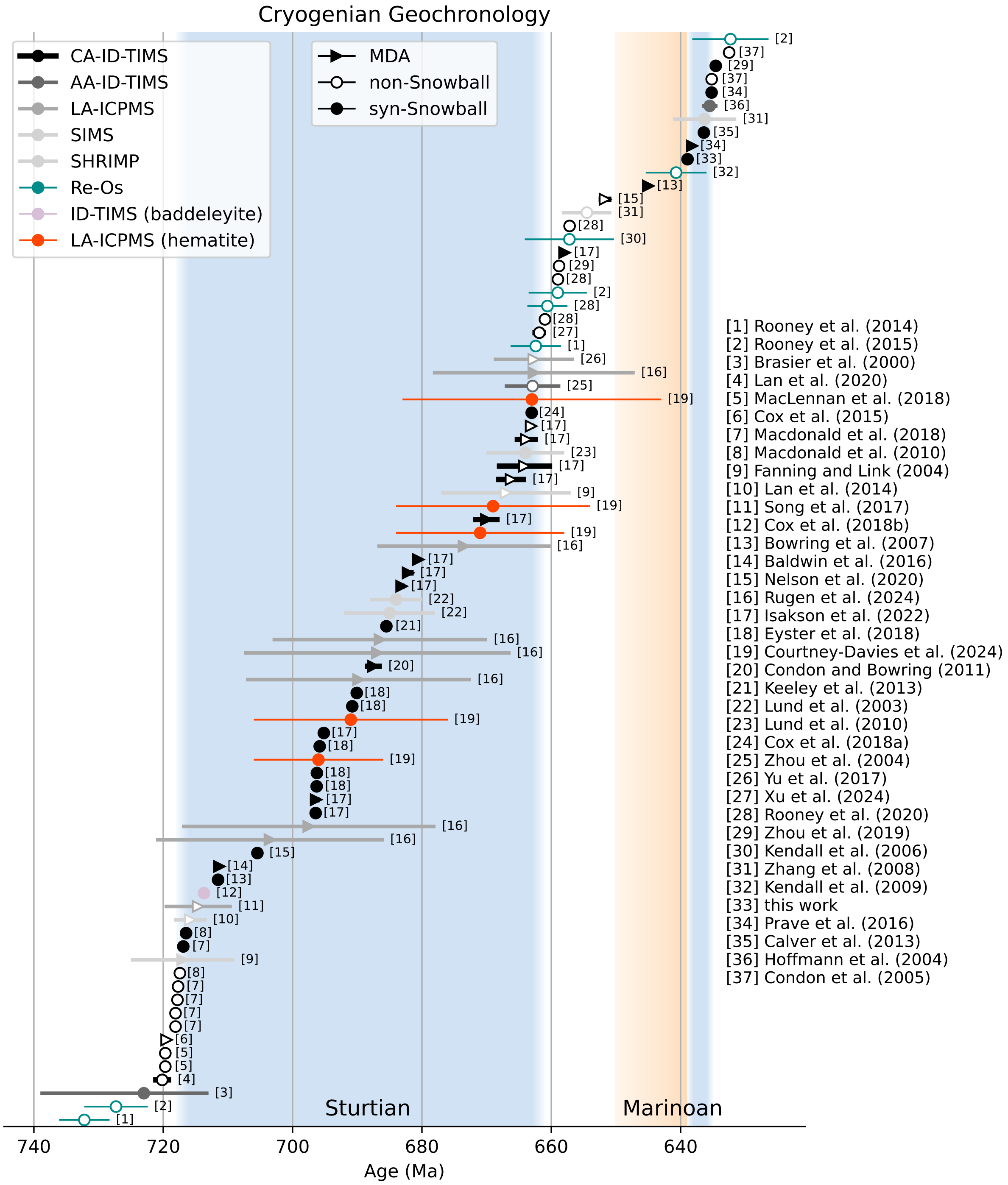

Fig. S1. Existing published radiometric geochronologic constraints for the Cryogenian. Symbols represent the analysis type (system and analytic method), age interpretation (syn-depositional or maximum age), and relation to snowball (either syn-snowball or not). With previously published constraints, approximately 10 My of uncertainty exists on the onset of the Marinoan snowball glaciation, shown as the orange interval. We interpret ca. 639 Ma Marinoan onset, constraining Marinoan duration to ca. 4 Myr, shown as the blue interval.

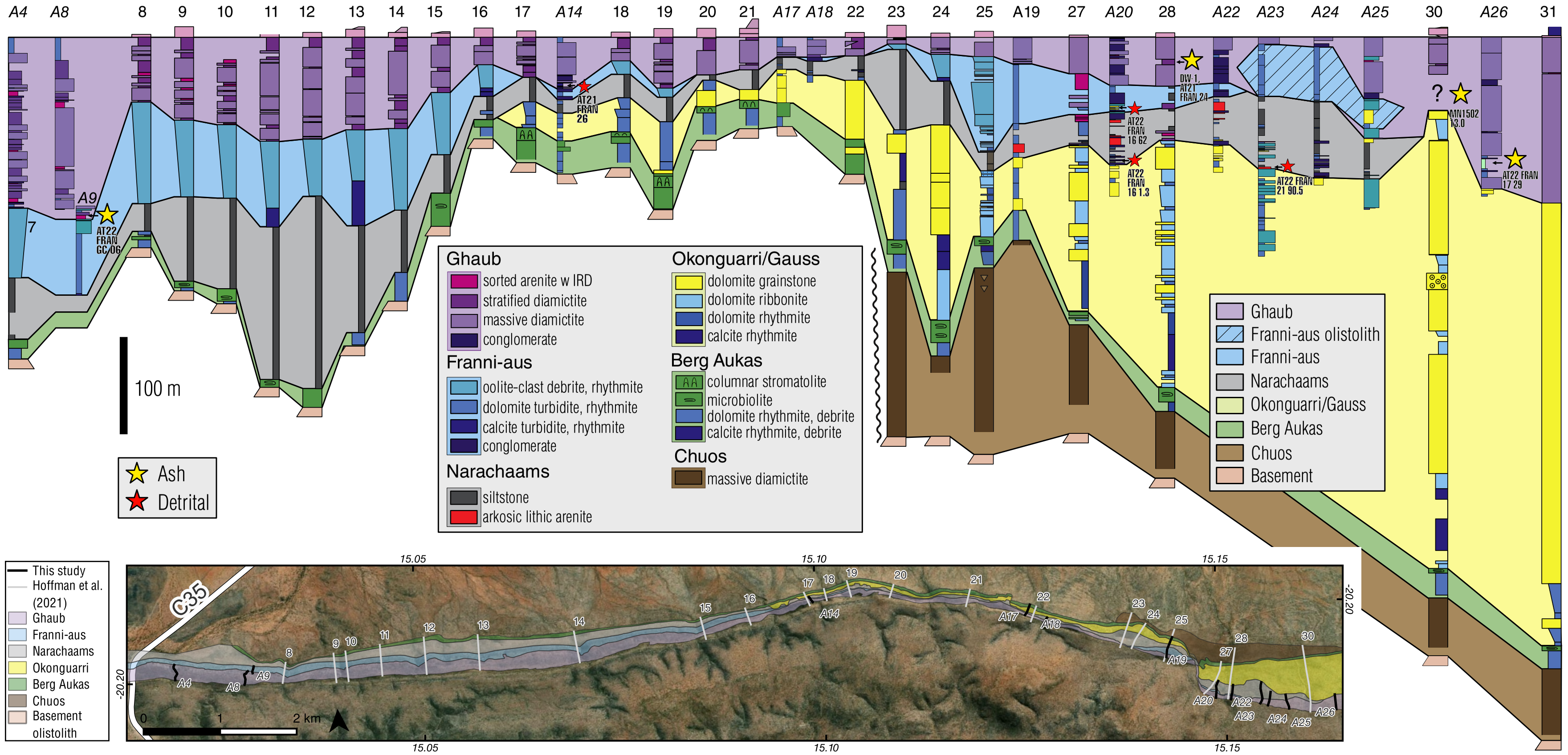

Fig. S2. Fence diagram modified from ref. (41) with sections from this study (prefixed with “A” and italicized). Datum is taken to be the base of the Keilberg Mb of Karibib Fm. The region from sections 27–30 shows stratigraphy we interpret as Narachaams Fm but ref. (41) and ref. (33) interpret as Ghaub Fm; see Figure S3.

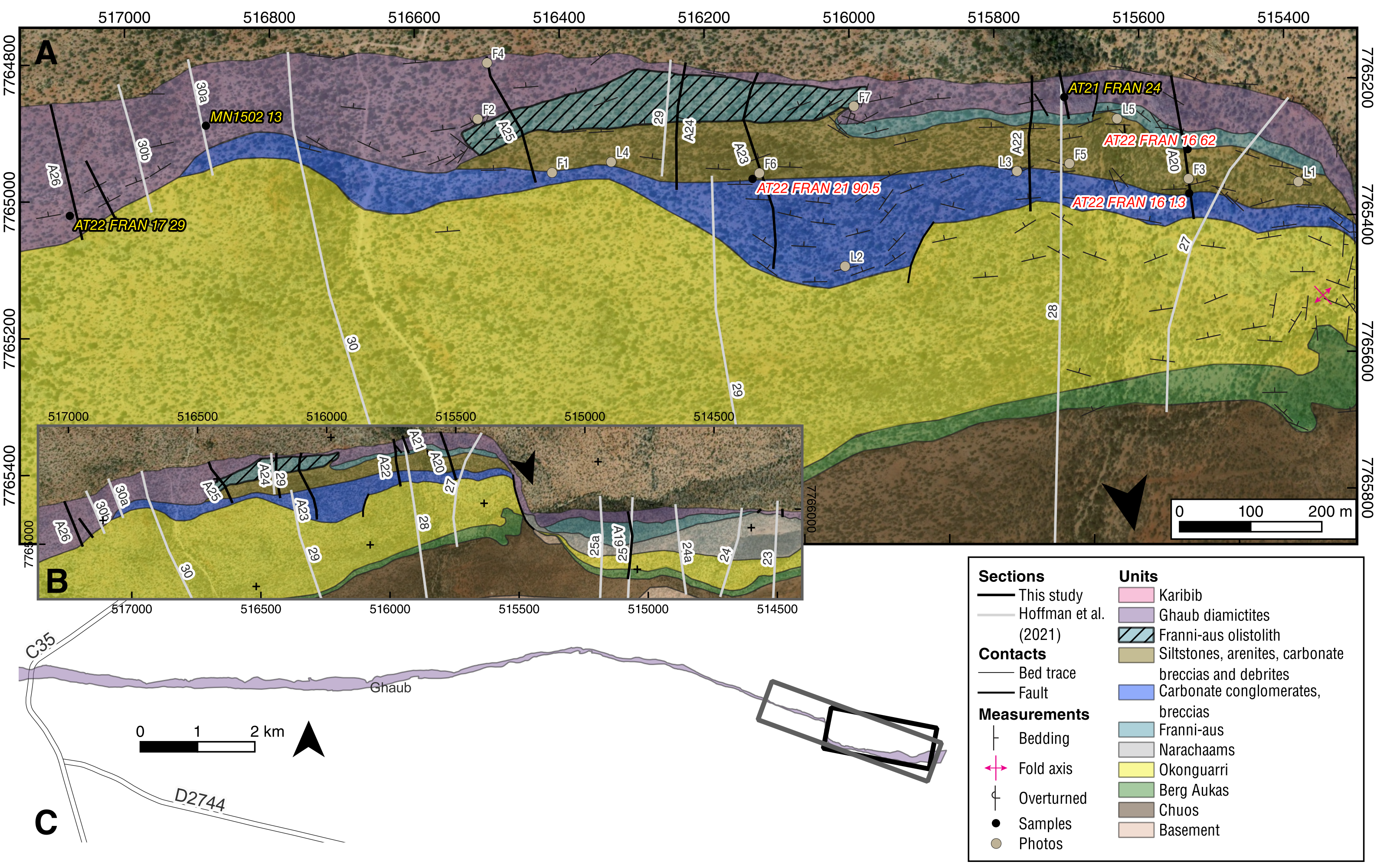

Fig. S3. Geologic map of eastern extent of eastern exposures of study area. Note that north points down for maps in A and B. **A** Locations of photos in Figures S10 & S9 are shown in as grey points. Samples with young, dominant volcanic zircon populations are shown with yellow labels. Detrital samples are shown in red. Stratigraphic section locations from this study are shown alongside those of ref. (41). The units in brown in blue between Okonguarri and Ghaub Fms reflect the ambiguous stratigraphy in this panel. A major surface exists on Okonguarri Fm, which ref. (41) attribute to erosion in a glacial channel. **B** Zoomed out view showing large structure that separates ambiguous panel in the east from panel with unambiguous units in the west. **C** Overview map showing exposure of Ghaub Fm along the study area and extents of A and B.

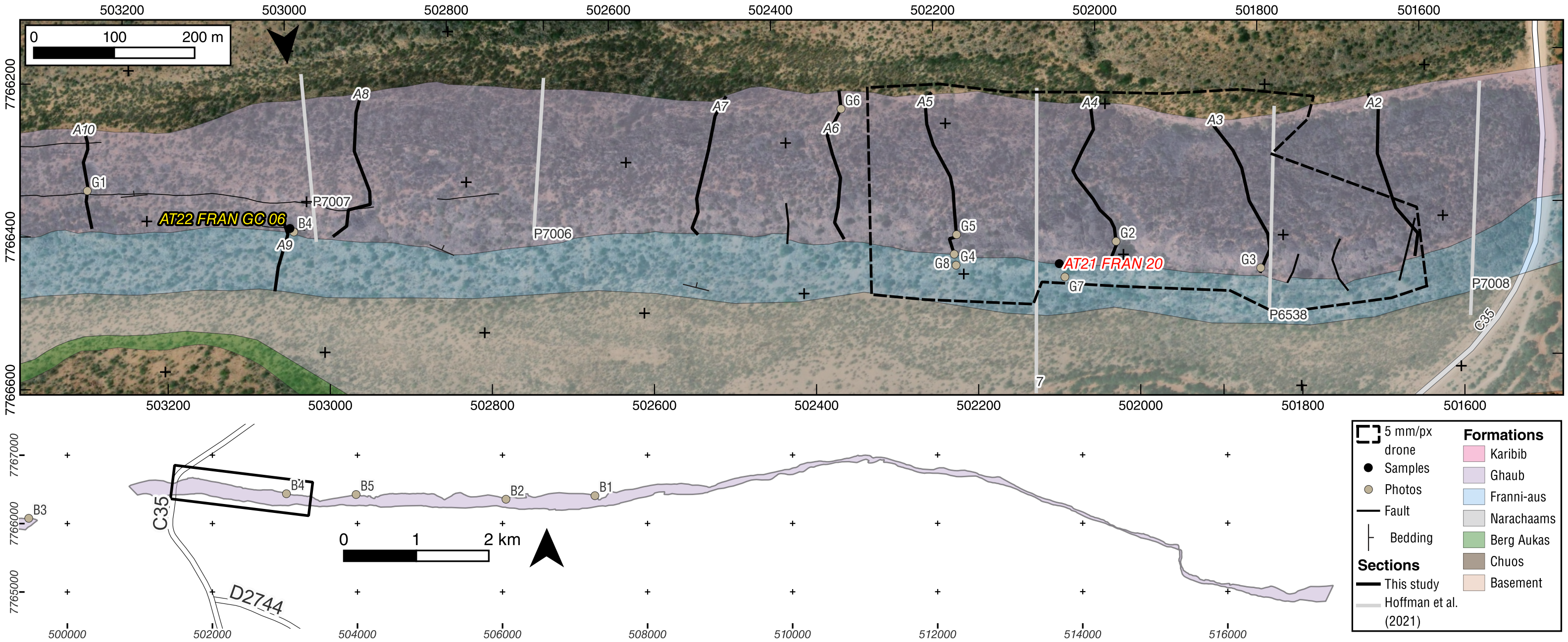

Fig. S4. Geologic map of western extent of study area near C35 road. Stratigraphic section locations from this study are shown alongside those of ref. (41). Locations of volcanic sample AT22 FRAN GC 06 and detrital sample AT21 FRAN 20 (Figure S30) are shown. Photo locations in Figures S11, S12, and S13 are shown as grey dots. The coverage area of 5 mm/px drone imagery is shown by the dashed line.

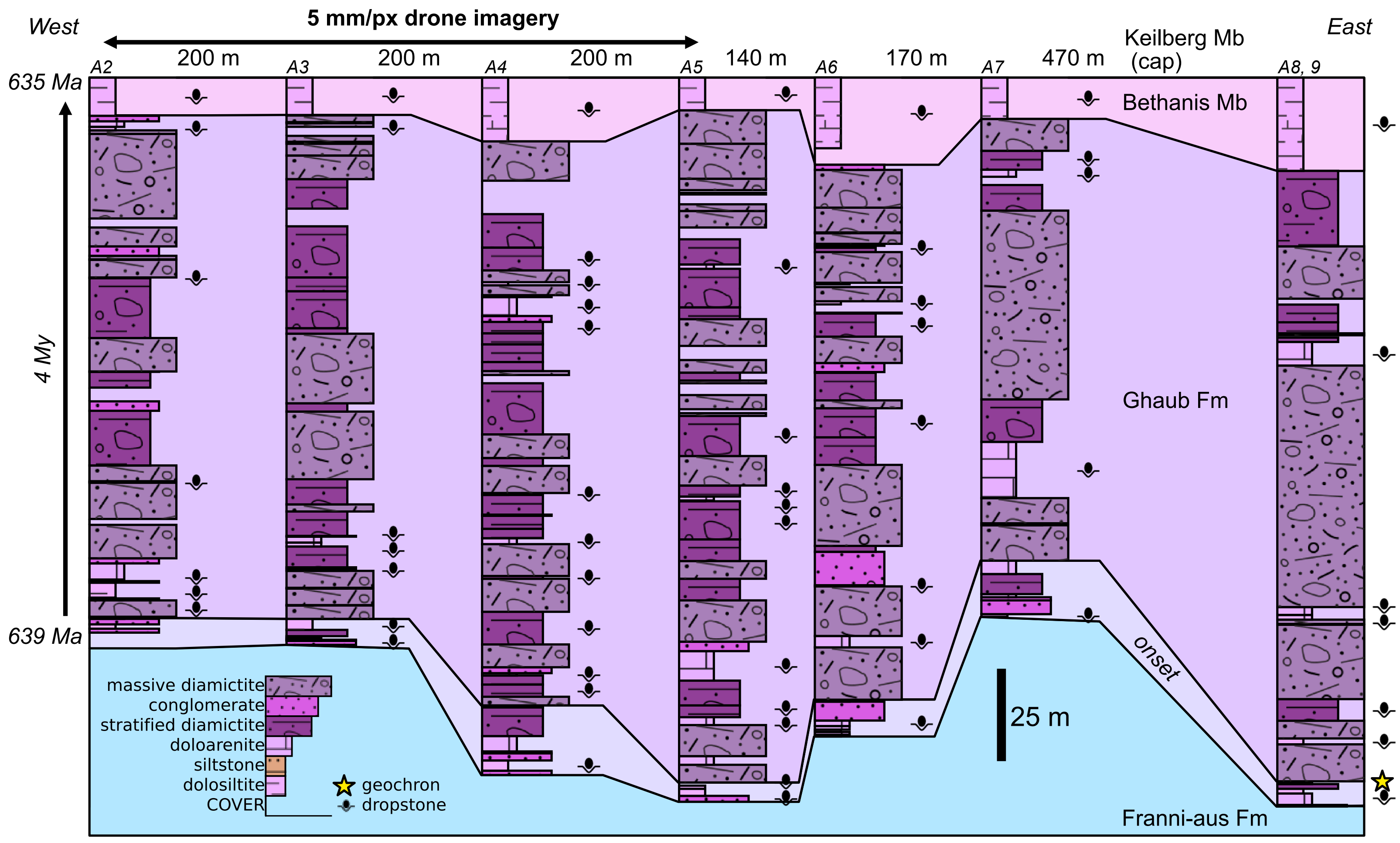

Fig. S5. Fence diagram of sections in Figure S4. Basal Ghaub Fm is always a stratified interval containing dropstones, which conformably rests on Frann-aus Fm. On the order of 10 grounding line cycles can be observed in the variation between stratified and massive diamictites, but confidently tracing these cycles across sections is not possible due to the complex stratigraphy. Datum for the fence is the base of the Keilberg Mb.

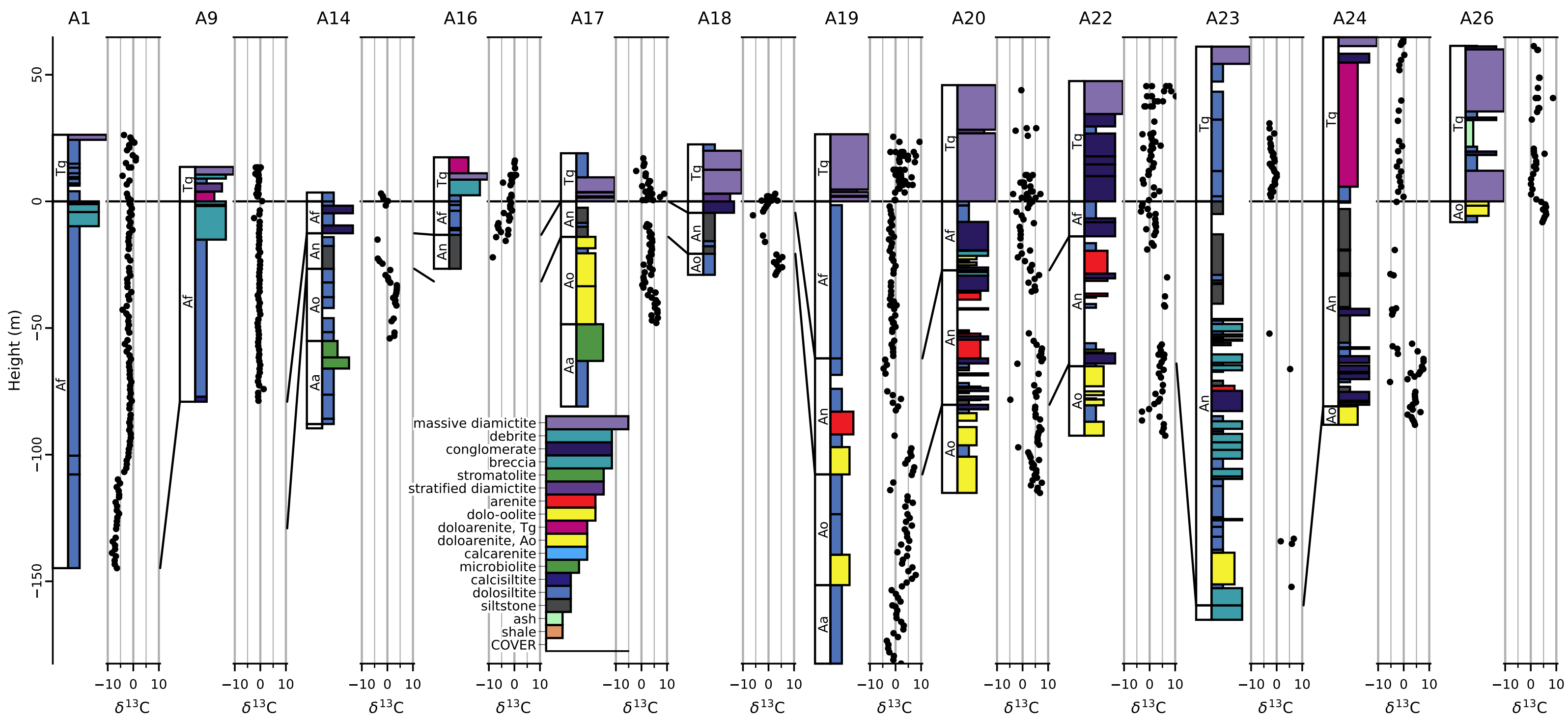

Fig. S6. Chemostratigraphy for detrital carbonate samples from 11 sections along study area. Franni-aus Fm thins to the east up the slope, and Ghaub Fm can often be identified by a stepwise increase in variability of stable isotopic composition. Tg: Ghaub Fm, An: Narachaams Fm, Ao: Okonguarri Fm, Aa: Berg Aukas Fm. Datum is basal Ghaub Fm.

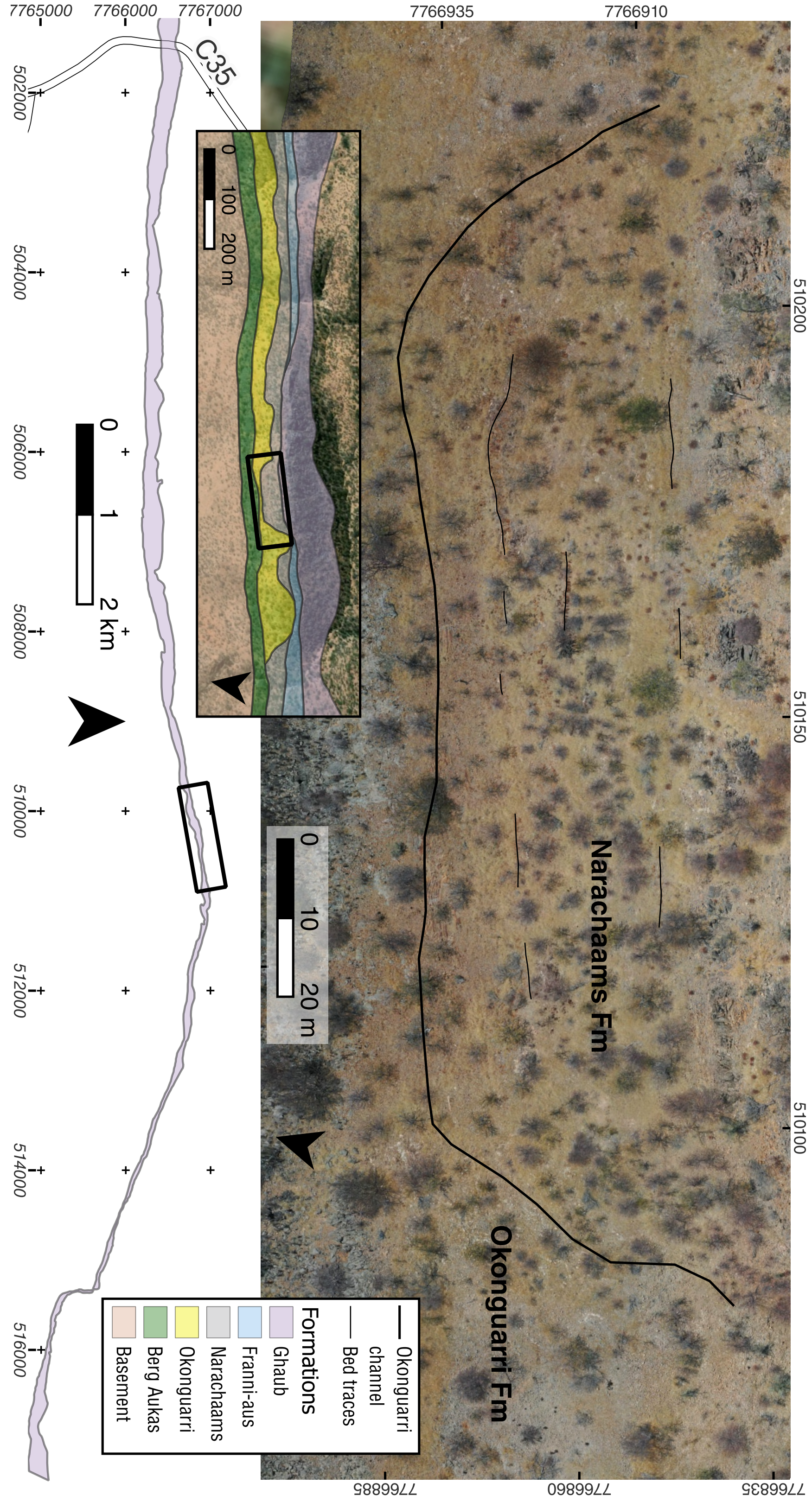

Fig. S7. Previously undescribed channel in Okonguarri Fm with approximately 30 m of relief; traces on 2 cm/px drone imagery.

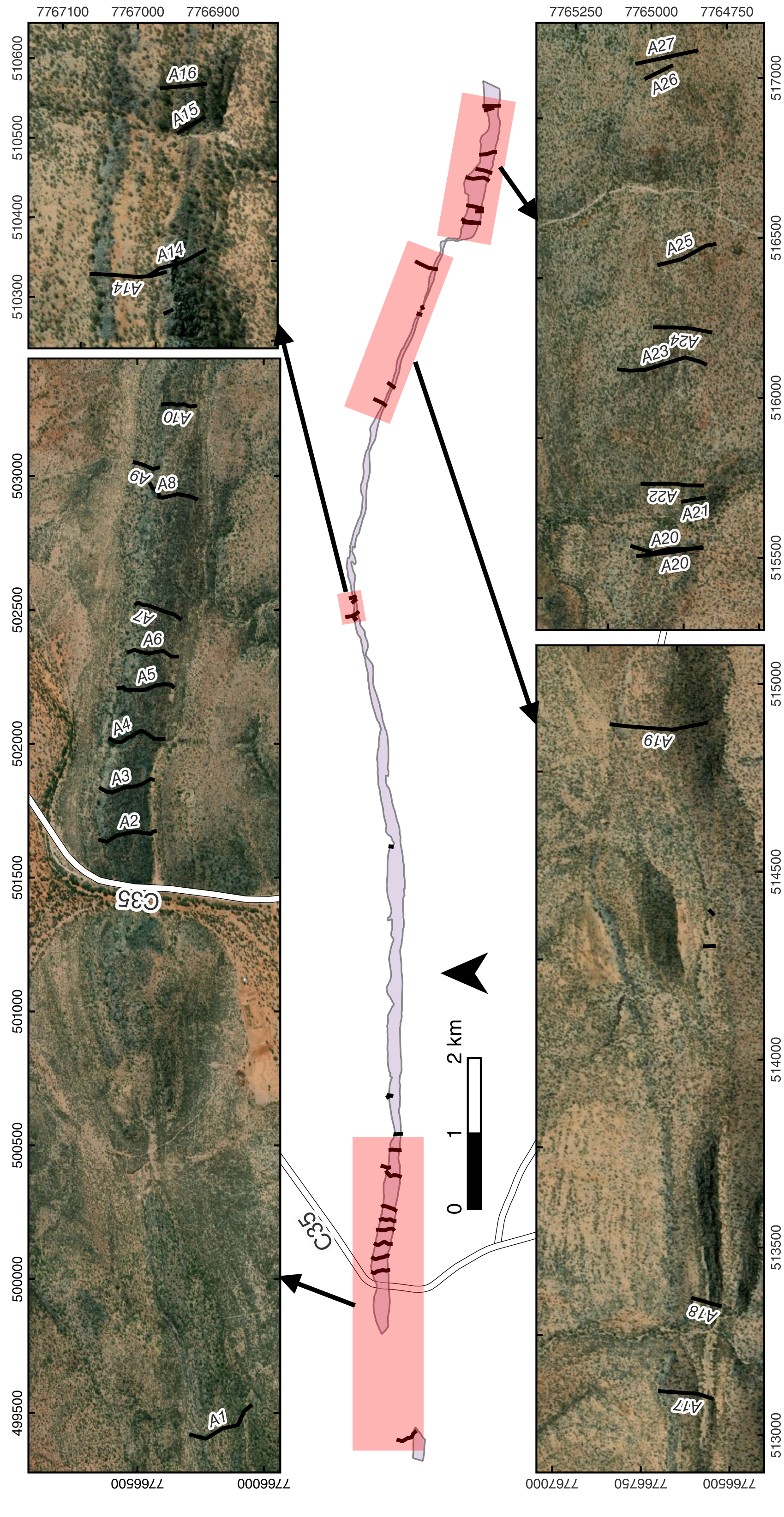

Fig. S8. Locations of stratigraphic sections from this study along Fransfontein Ridge. Purple polygon shows extent of Ghaub Fm in study area.

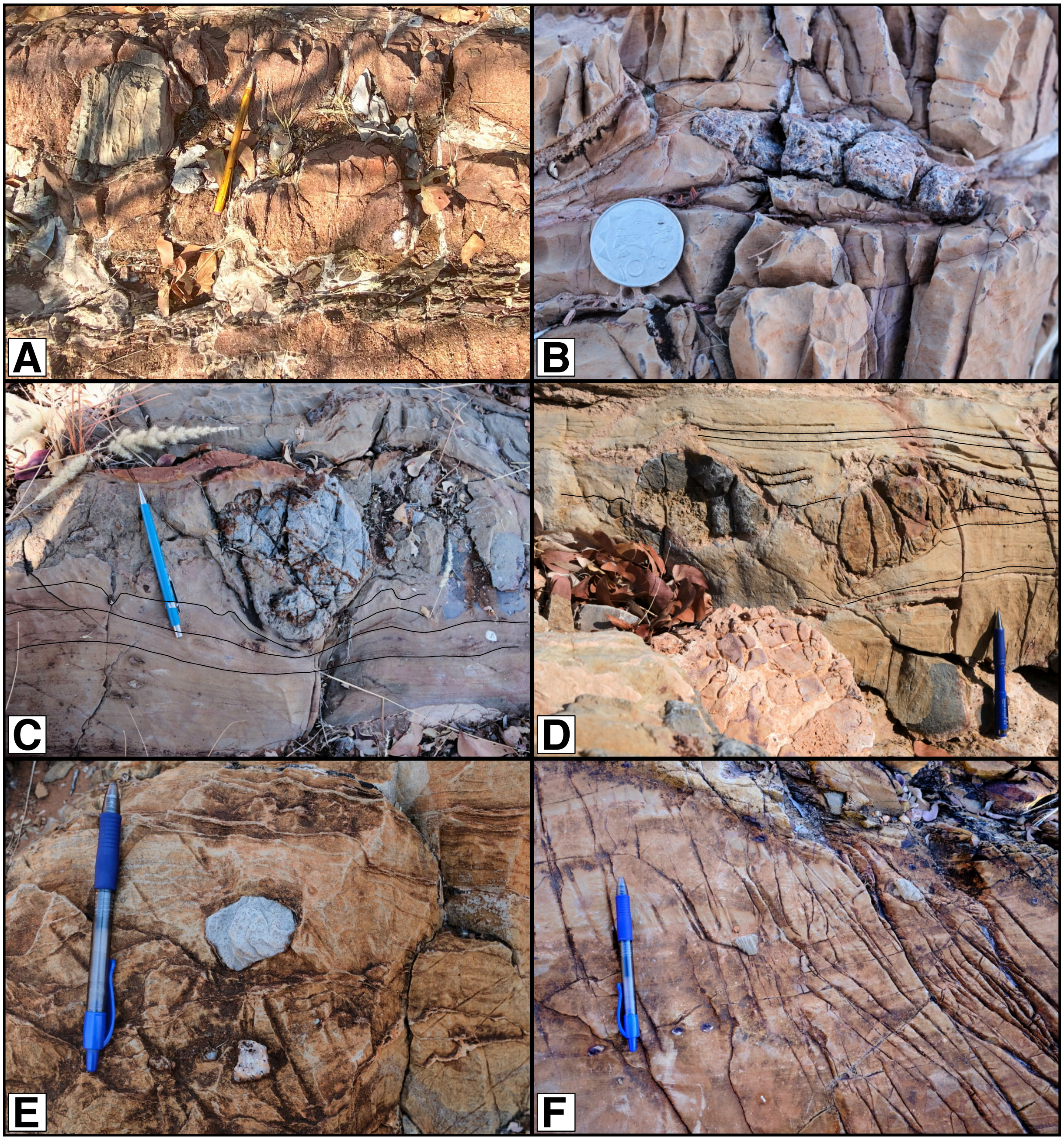

Fig. S9. Outsized clasts and lonestones observed in ambiguous interval of carbonate conglomerates, breccias, siltstones, arenites, and debrites (Figure S3). Location labels L1–L5 are shown in Figure S3. **A** L1, cobble dolostone clast within 10 cm scale graded beds of arkose with abundant dolostone lithic fragments. Pencil is 14.3 cm long. **B** L2, Lonestone of silicified oolite (?) within faintly laminated dolosiltite. Coin is 21.5 mm in diameter. **C** L2, clastic horizon with penetration of laminated dolosiltite beds below. Clasts are weakly silicified; clastic interval lacks stratification. Pencil is 14.5 cm long. **D** L3, two cobble dolostone lonestones within cm-scale bedded dolosiltite. Lonestones are clearly onlapped, including by distinct beds with outsized granule-pebble clasts. Bed penetration cannot be evaluated with the state of textural preservation. Pen is 14.3 cm long. **E** L4, pebble lonestones within mm-scale laminated dolosiltite. **F** L5, pebble lonestones within mm-scale laminated dolosiltite.

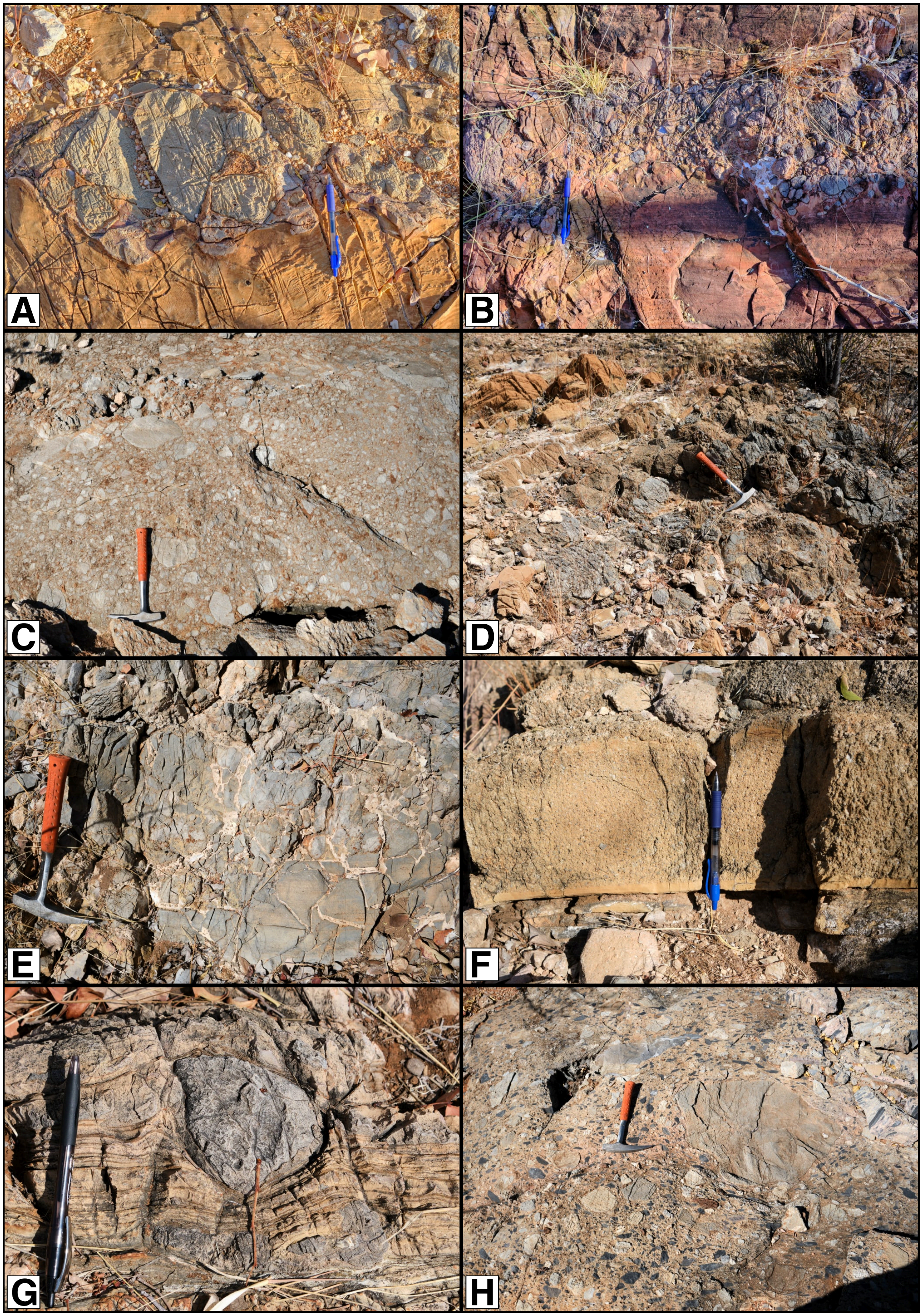

Fig. S10. Facies typical of Ghaub Fm and ambiguous underlying stratigraphy of carbonate conglomerates, breccias, siltstones, arenites, and debrites (Figure S3). Location labels L5, F1–F6 are shown in Figure S3. **A** L5, pod of subangular monomict dolostone pebbles and cobbles within laminated dolosiltite. **B** L5, monomict dolostone pebble-cobble conglomerate within laminated dolosiltite. **C** F1, monomict dolostone breccia. Hammer is 33 cm long. **D** F5, monomict dolostone pebble-boulder conglomerate overlain by laminated dolosiltite. **E** F6, intraclast breccia of parallel stratified doloarenite. **F** F3, quartz arenite with up to granule quartz and abundant dolostone lithic fragments. **G** F2, cobble dropstone exhibiting clear bed penetration. **H** F4, polymict massive diamictite with pebble-boulder clasts, high clast density.

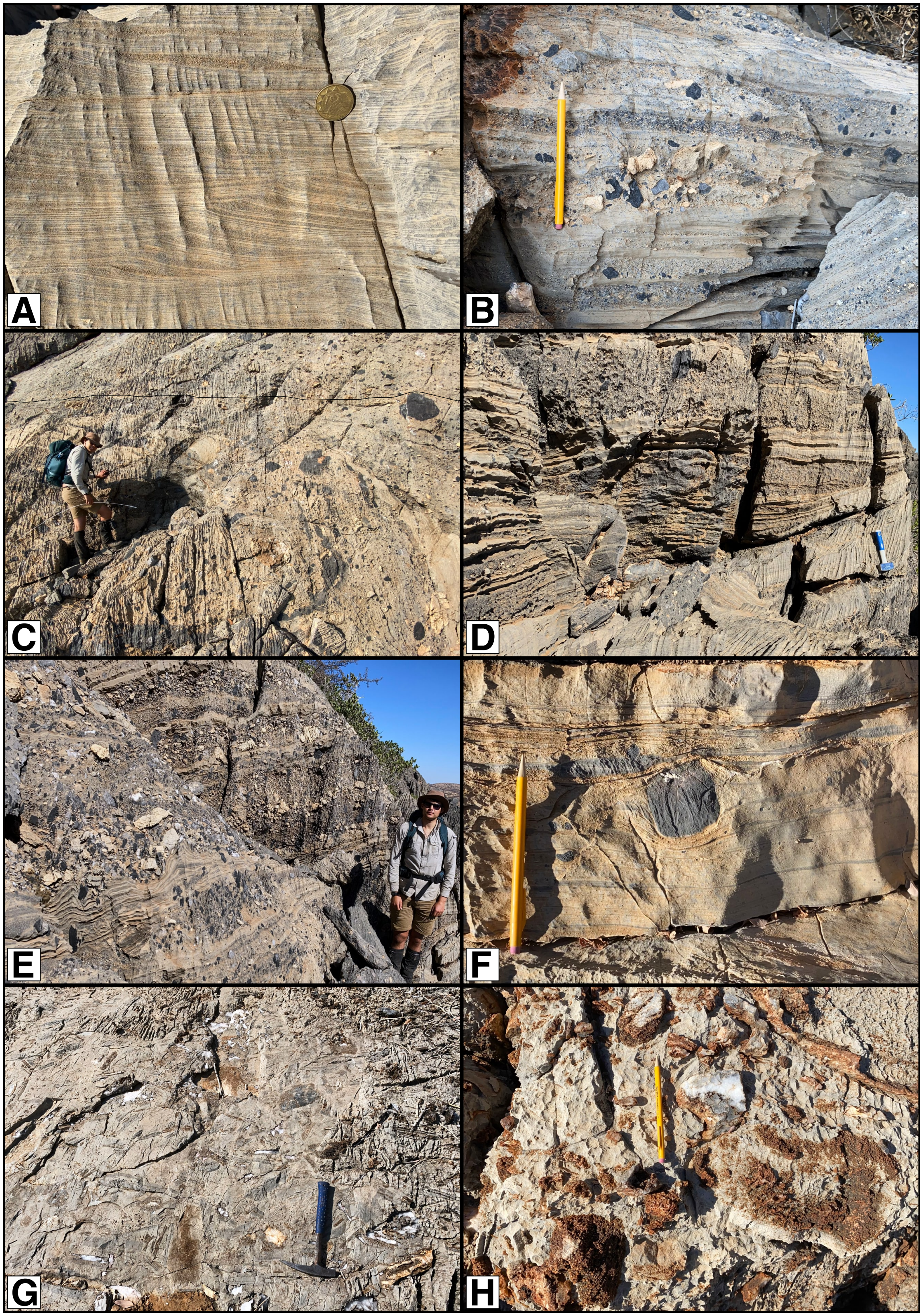

Fig. S11. Facies typical of Ghaub Fm and upper Franni-aus Fm (Figure S4). Location labels G1–G8 are shown in Figure S4. **A** G1, climbing current ripples in doloarenite. Coin is 22.6 mm in diameter. **B** G2, interbedded parallel laminated dolosiltites and doloarenites and granule-pebble polymict clastic intervals with outsized cobbles indicating a probably meltout origin. **C** G3, folded stratified diamictite including laminated dolosiltite overlain by massive diamictite; base of massive diamictite is shown by fine black line. Note folded decimeter-scale rip-up clasts of the laminated buff fine doloarenite, which folds down in the right part of the image. **D** G5, channel form cut into laminated doloarenite and filled with alternating parallel and cross-laminated doloarenites and granule beds.**E** G5, dropstone-bearing parallel and interbedded dolosiltites and doloarenites which are soft-sediment deformed by pebble-cobble conglomerates. **F** G8, dropstones in parallel laminated dolosiltite. **G** G7, upper Franni-aus Fm; tabular intraclast breccia of dolosiltite rhythmites with void-filly sparry calcite cements and weak silicification. **H** G4, upper Franni-aus Fm; conglomerate with silicified oolite clasts and tabular dolosiltite clasts.

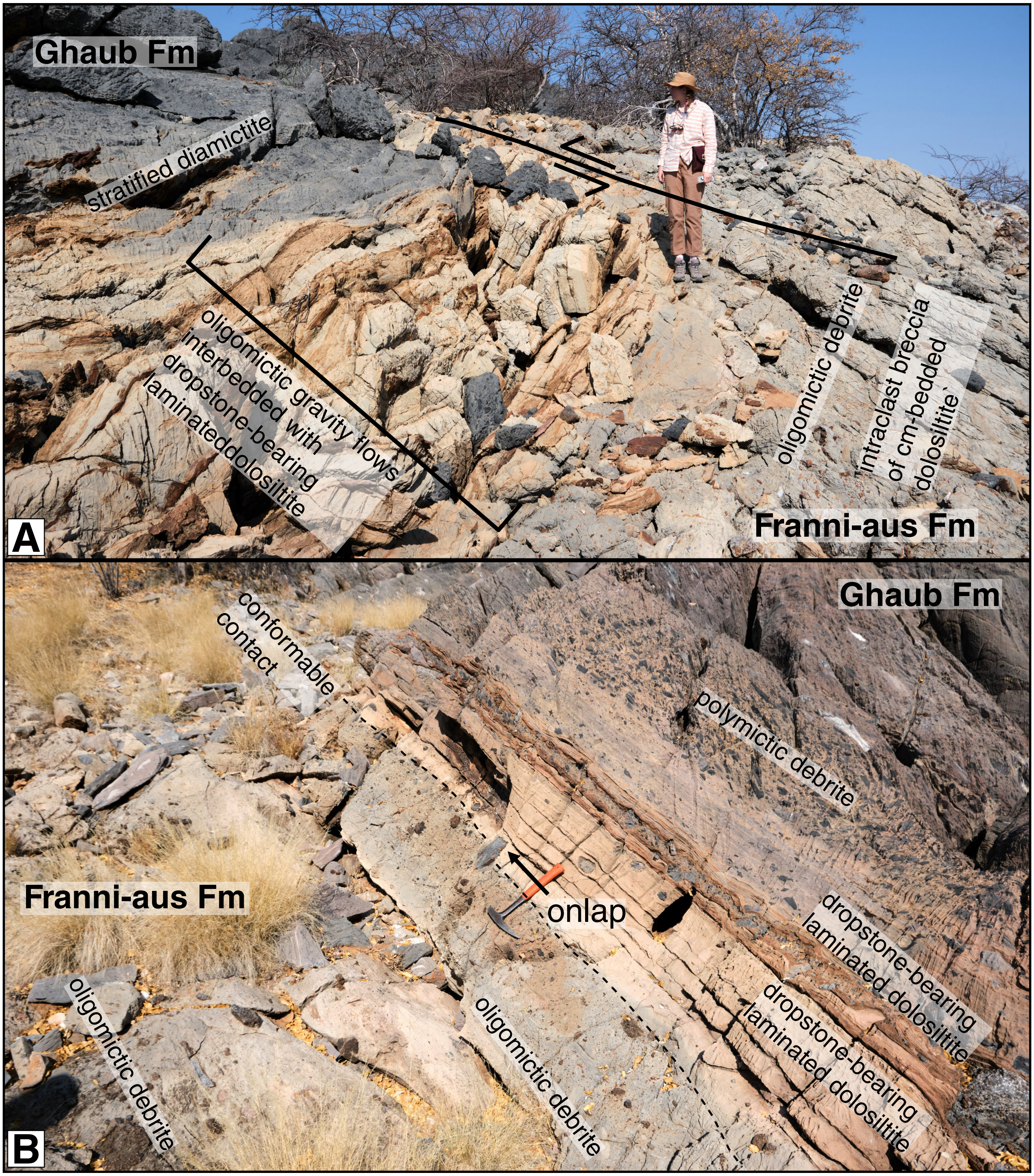

Fig. S12. A Conformable contact from Franni-aus Fm to stratified basal Ghaub Fm at B5 in Figure S4. **B** Basal Ghaub Fm at B2. Note the clast protruding from the oligomictic debrite, which is onlapped by laminated dolosiltite. At both locations, basal Ghaub Fm comprises laminated dolosiltite with dropstones and debrite, indicated a pro-grounding line location.

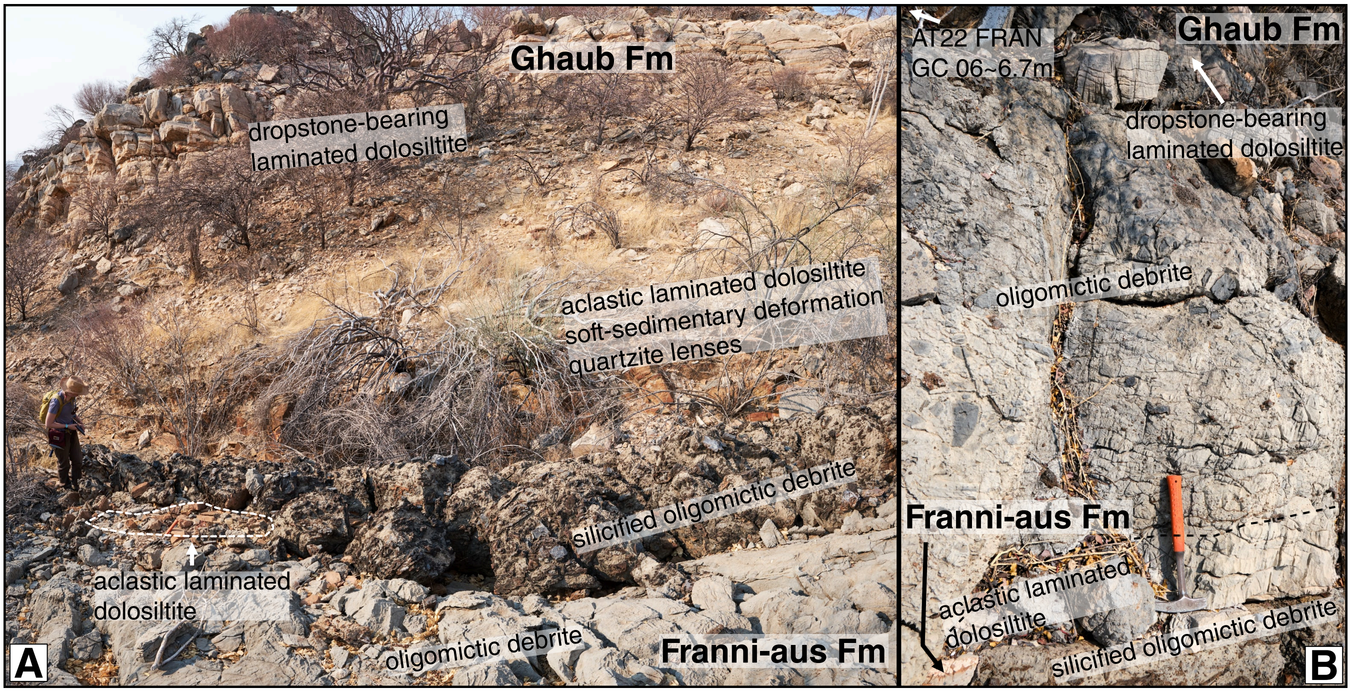

Fig. S13. **A** Basal Ghaub Fm on Franni-aus Fm at B3 in Figure S4; measured in section A1. Note a pod of laminated orange dolosiltite that is erosionally truncated on either side by the silicified clast breccia, denoted by the black dashed line. The first dropstones are observed on the cliff-forming outcrop towards the top of the image. The subcropping intervening stratigraphy is laminated dolosiltites with occasional $<$20 cm coarse arkoses. **B** Reverse-graded debrite between dolosiltite horizons at conformable contact between Franni-aus Fm and Ghaub Fm at B4, immediately beneath sampling location for AT22 FRAN GC 06. Silicified clast debrite can be discerned at the bottom of the image. The first dropstones are observed in the upper dolosiltite.

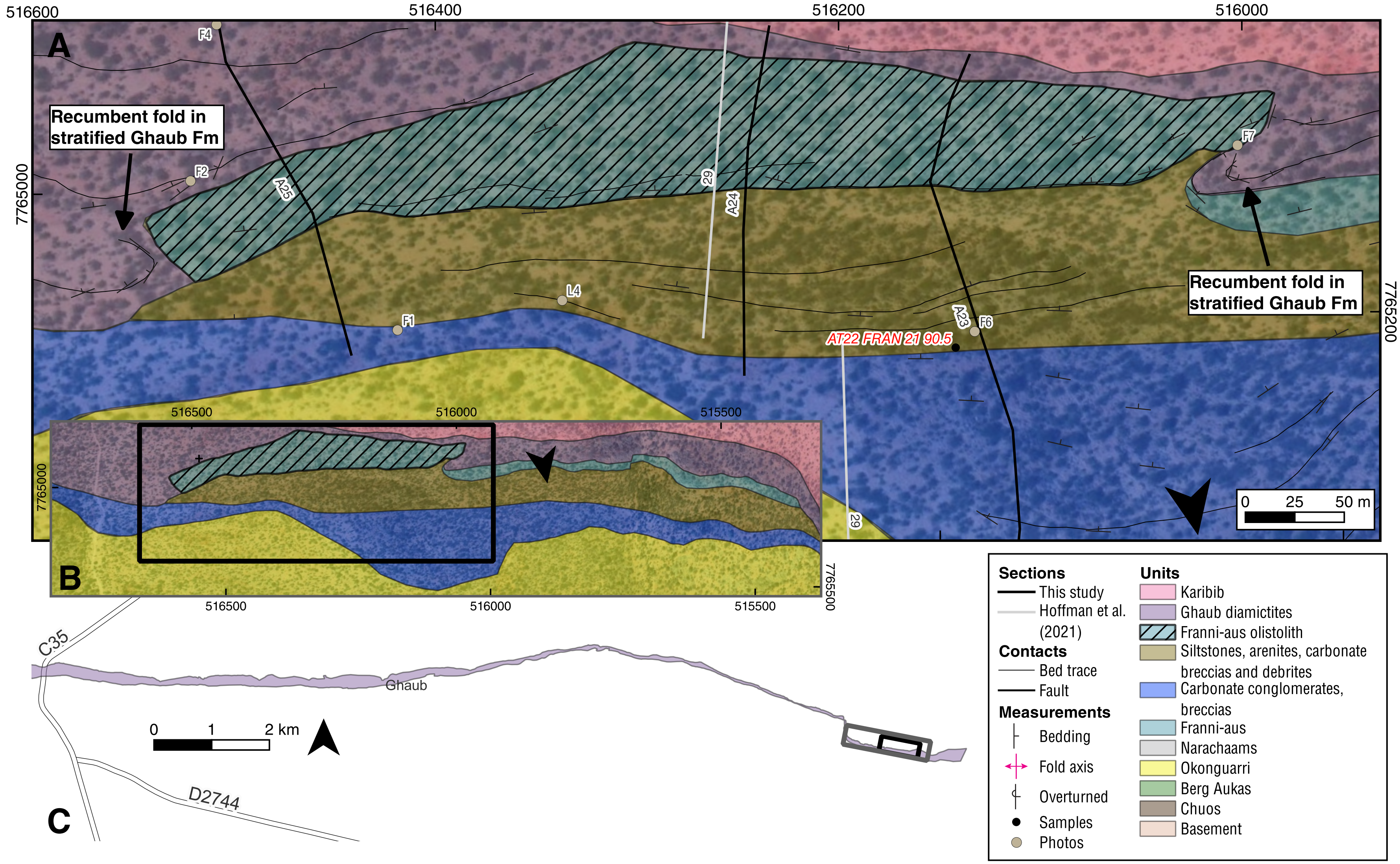

Fig. S14. **A** Detailed mapping of olistolith of Franni-aus Fm within Ghaub Fm. Stratified Ghaub Fm, including the horizon containing AT21 FRAN 24 on the western margin, folds over abutting the olistolith on both sides. The margins of the olistolith are intensely soft sediment folded (Figure S15). **B** Eastern exposure of study area shown in Figure S3A; extent of panel A is shown. **C** Overview map showing exposure of Ghaub Fm along the study area and extents of A and B.

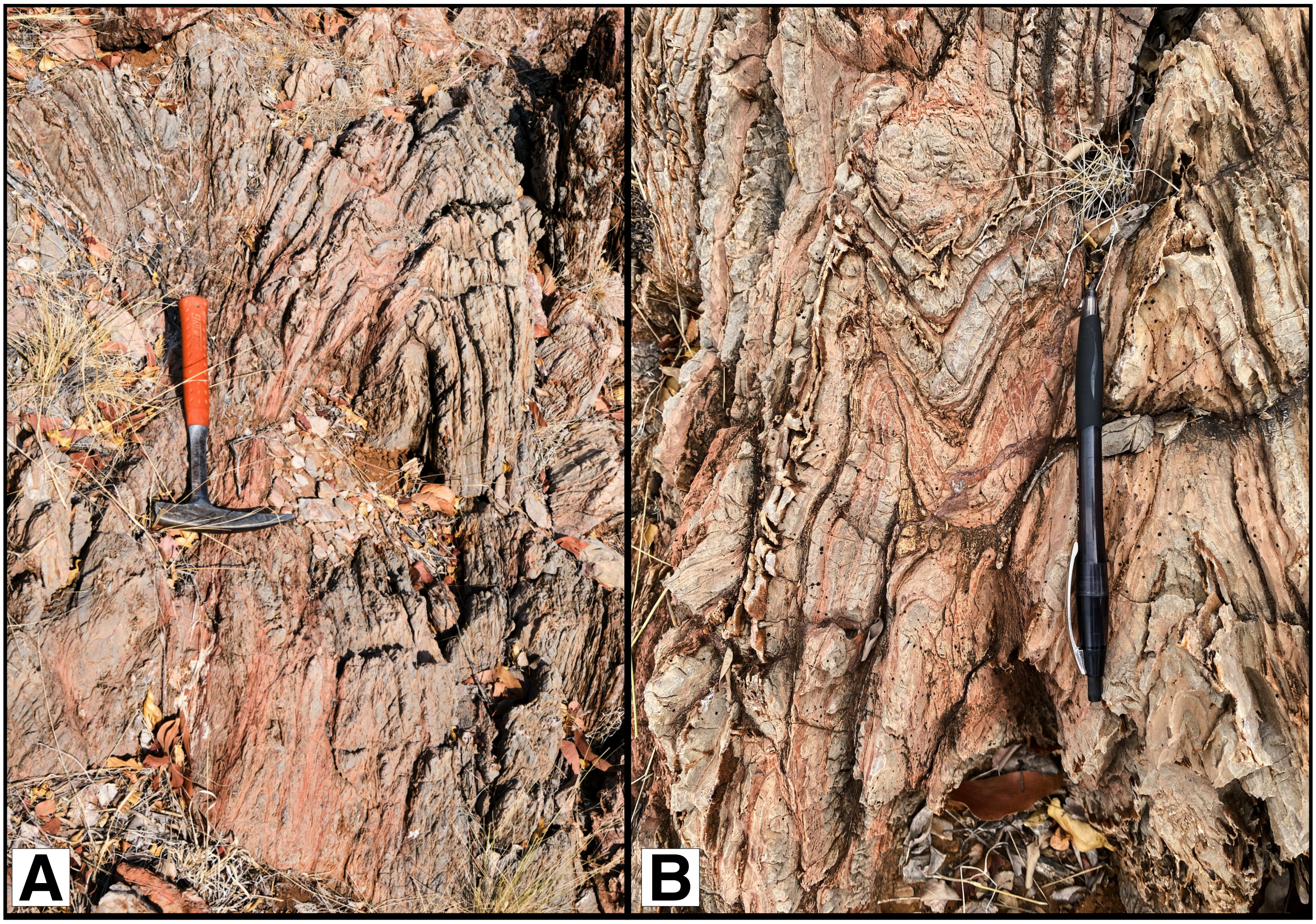

Fig. S15. A Soft-sedimentary deformation of Franni-aus formation at western boundary of the olistolith (Figure S3, S14, F7); hammer points up section. **B** Close-up of panel A showing some brittle behavior of silicified horizons, consistent with early silicification of Franni-aus Fm.

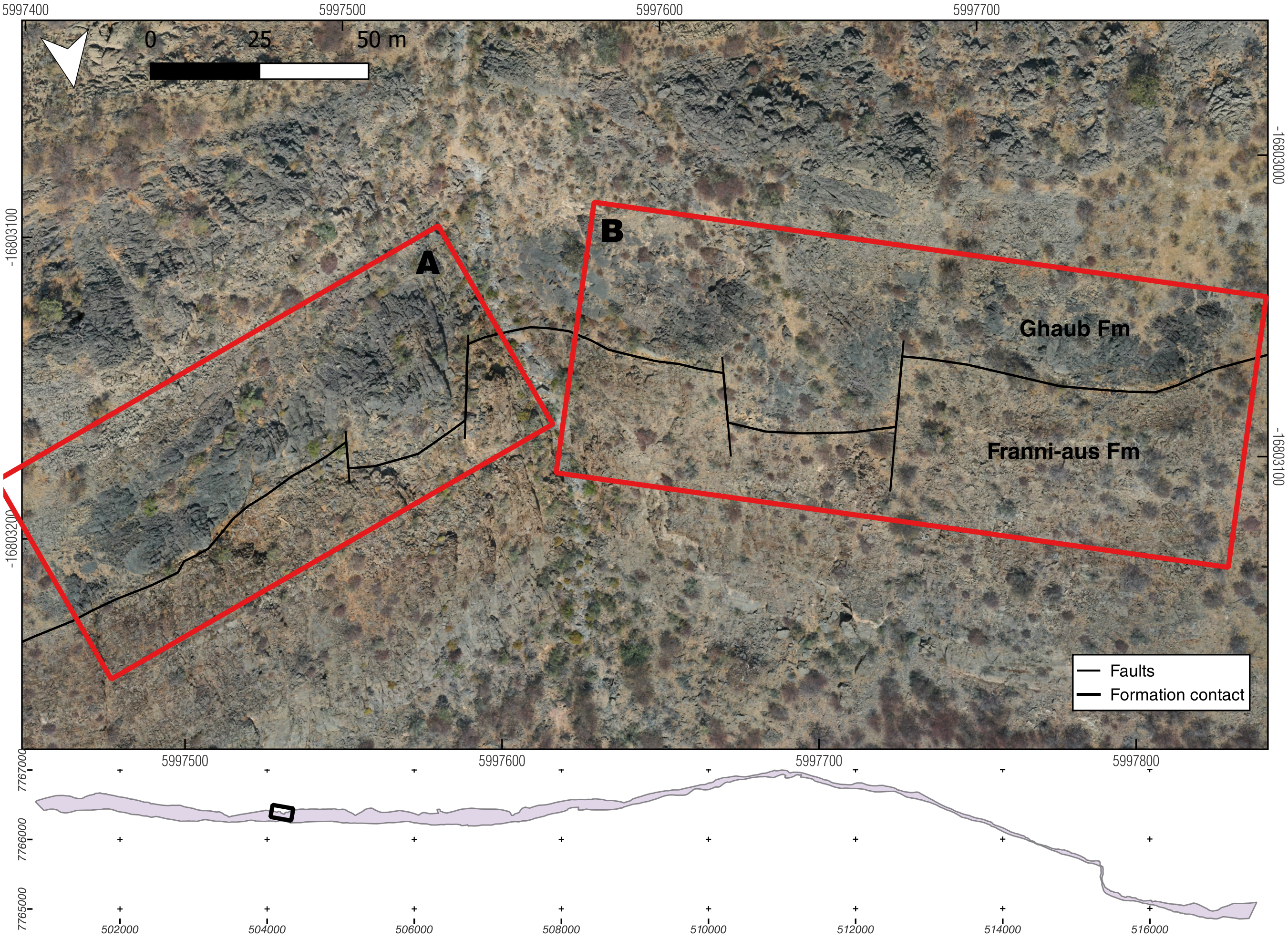

Fig. S16. Panel where ref. (42) report a grooved surface on Franni-aus Fm. Where they interpret an erosional base of Ghaub Fm, we instead map early faults that offset the base of Ghaub Fm, whose basal sediments are always stratified with dropstones. Location within study area show on overview map below. **A** shows approximate extent of Figure S17A, and **B** shows approximate extent of Figure S17B.

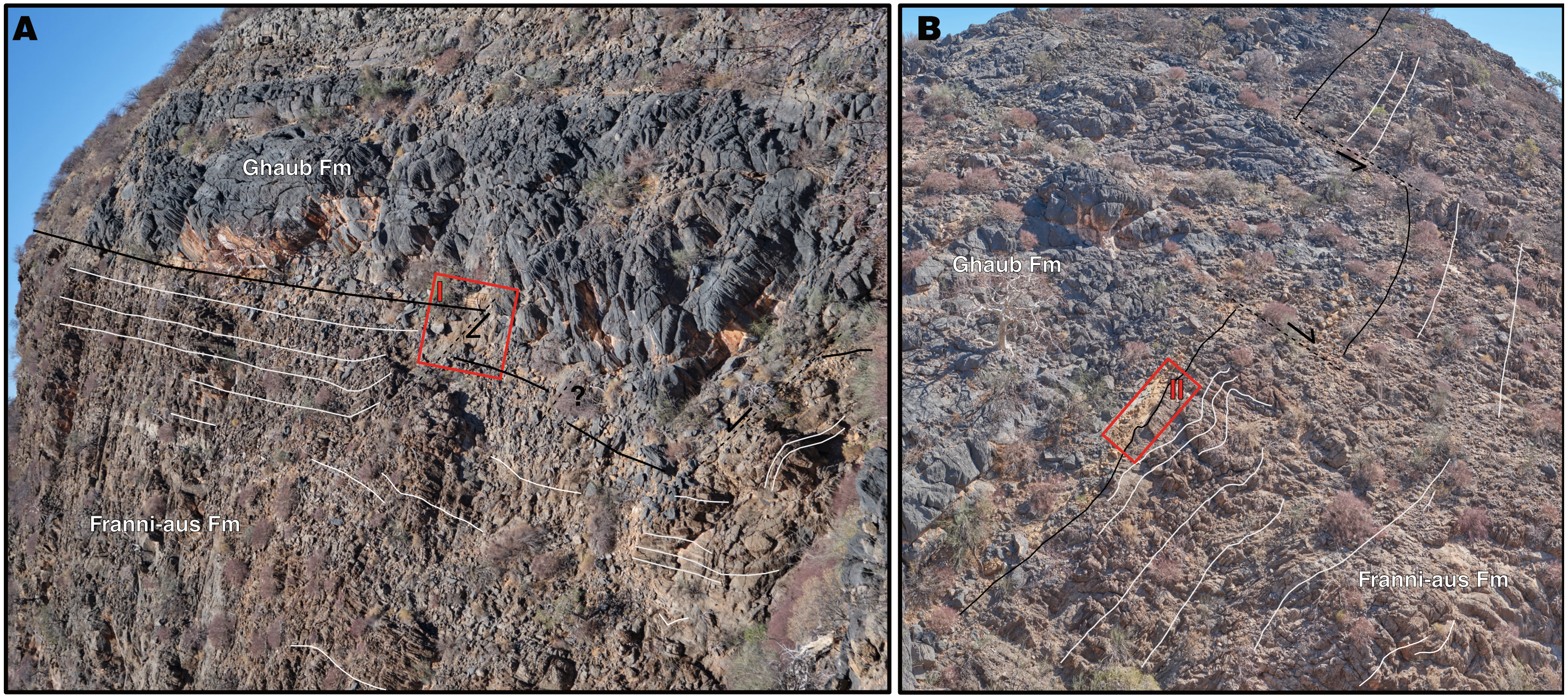

Fig. S17. Contact between formations is traced in black. Faults shown with dashed lines. Traces of beds within Franni-aus Fm are shown in white. **A** Photo looking east towards exposure of Ghaub and Franni-aus Fms. Approximate extent of Figure S18 shown in red (I). **B** Photo looking west. Approximate extent of Figure S19B shown in red (II).

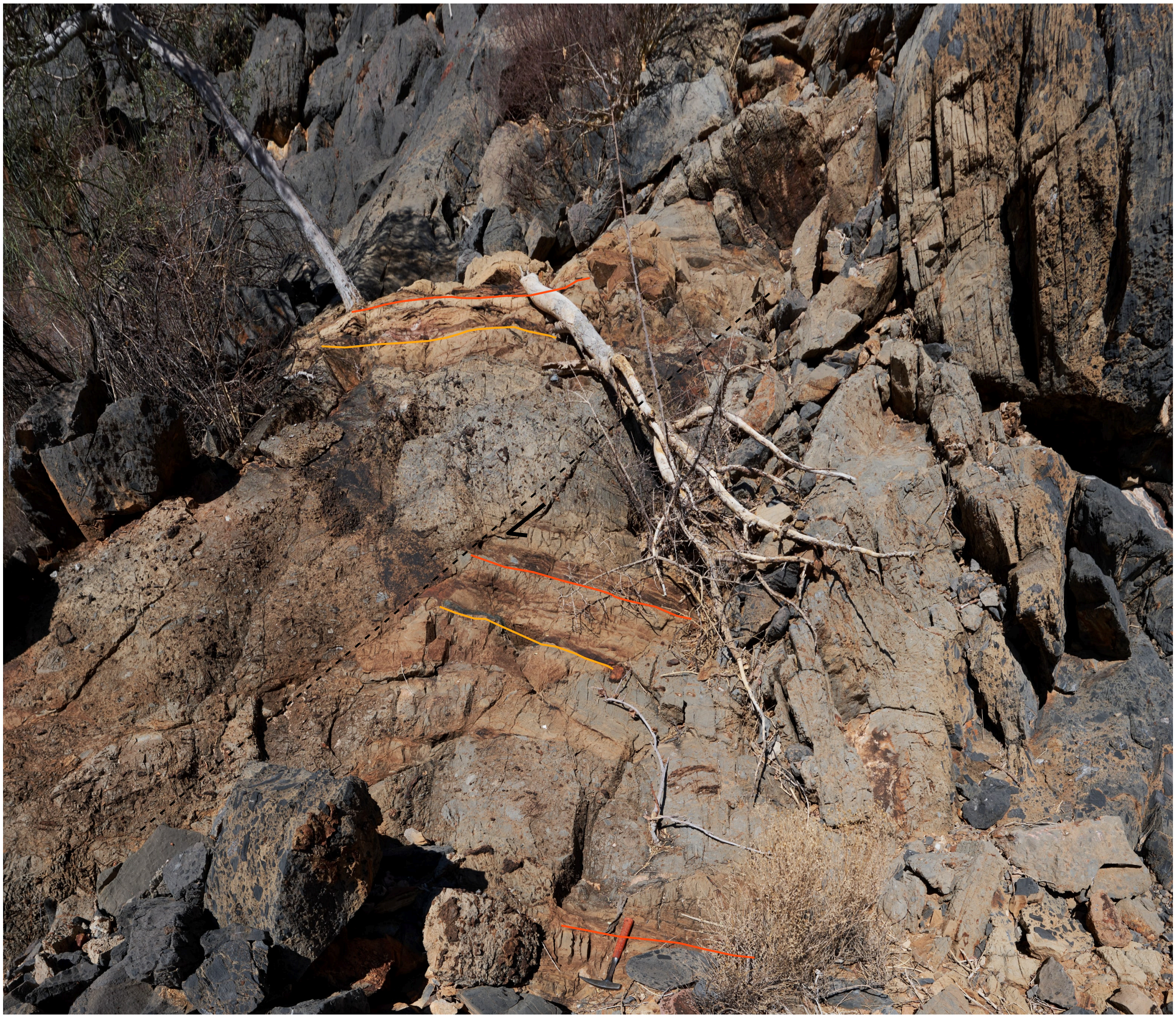

Fig. S18. Hammer handle points up section. High angle offsets of basal dropstone-bearing stratified Ghaub Fm, where two marker beds are traced in different shades of orange. Blocks of Franni-aus Fm are incorporated into a breccia associated with the fault.

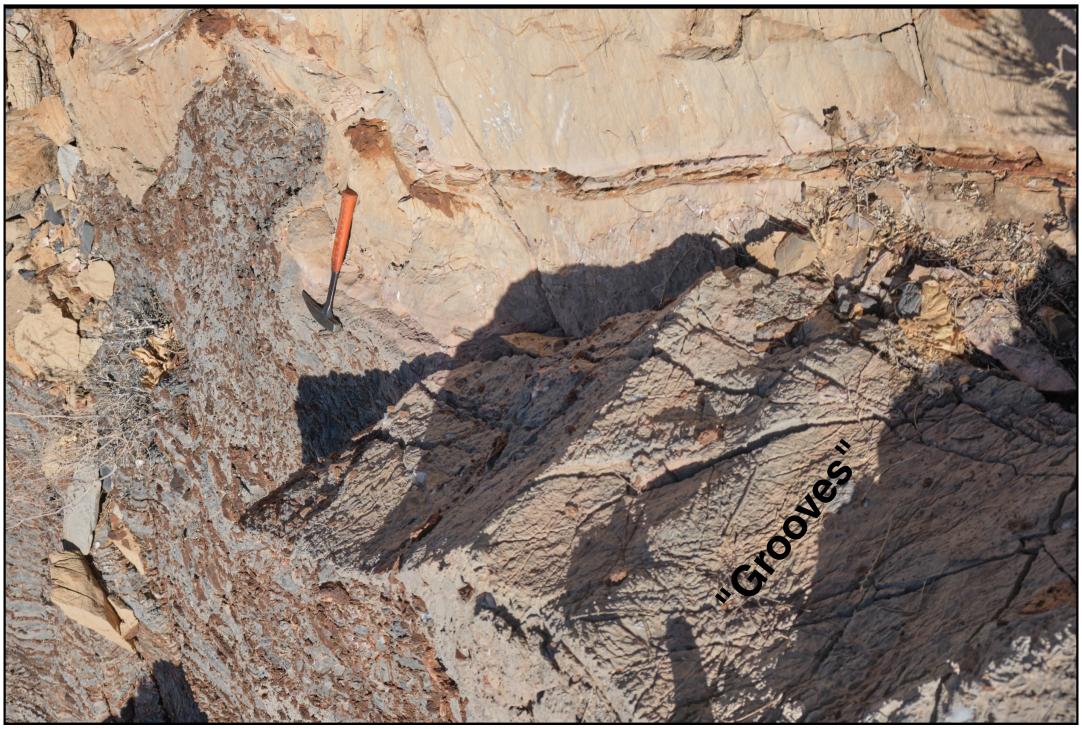

Fig. S19. Small soft-sedimentary folds on uppermost Franni-aus Fm interpreted by ref. (42) as glacial grooves.

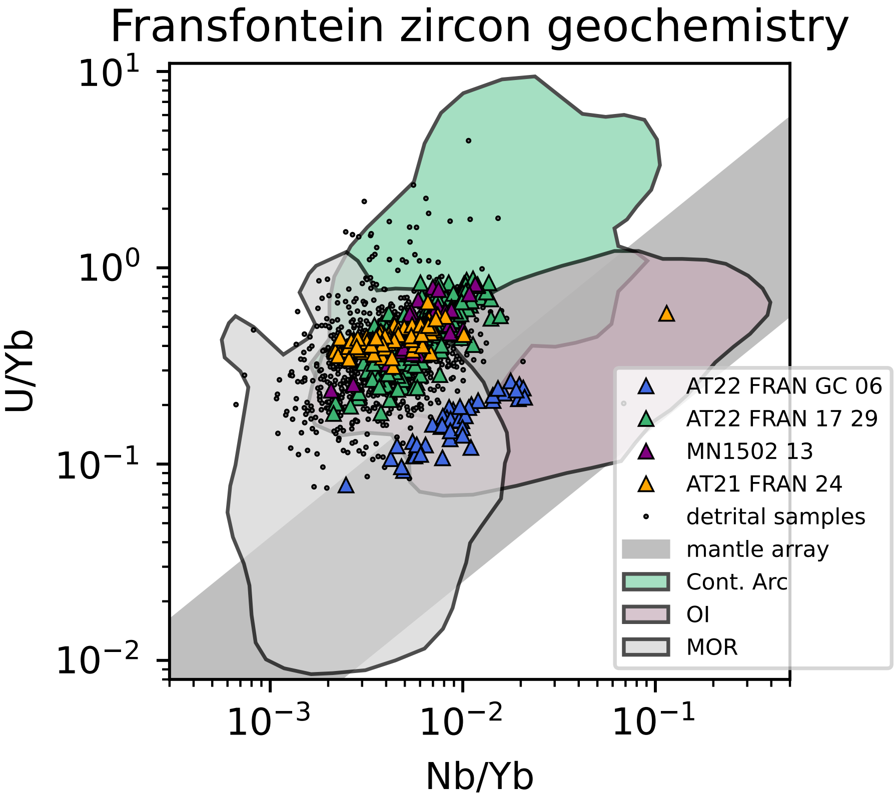

Fig. S20. Fransfontein ash zircon geochemistry with 95% confidence contours for magmatic environment from ref. (87). Each sample forms a coherent unimodal geochemical population, and AT22 FRAN GC 06 can be distinguished from other samples by uniquely occupying the mantle array. Small grey dots show the distribution of detrital zircon geochemistries (Section S3.C).

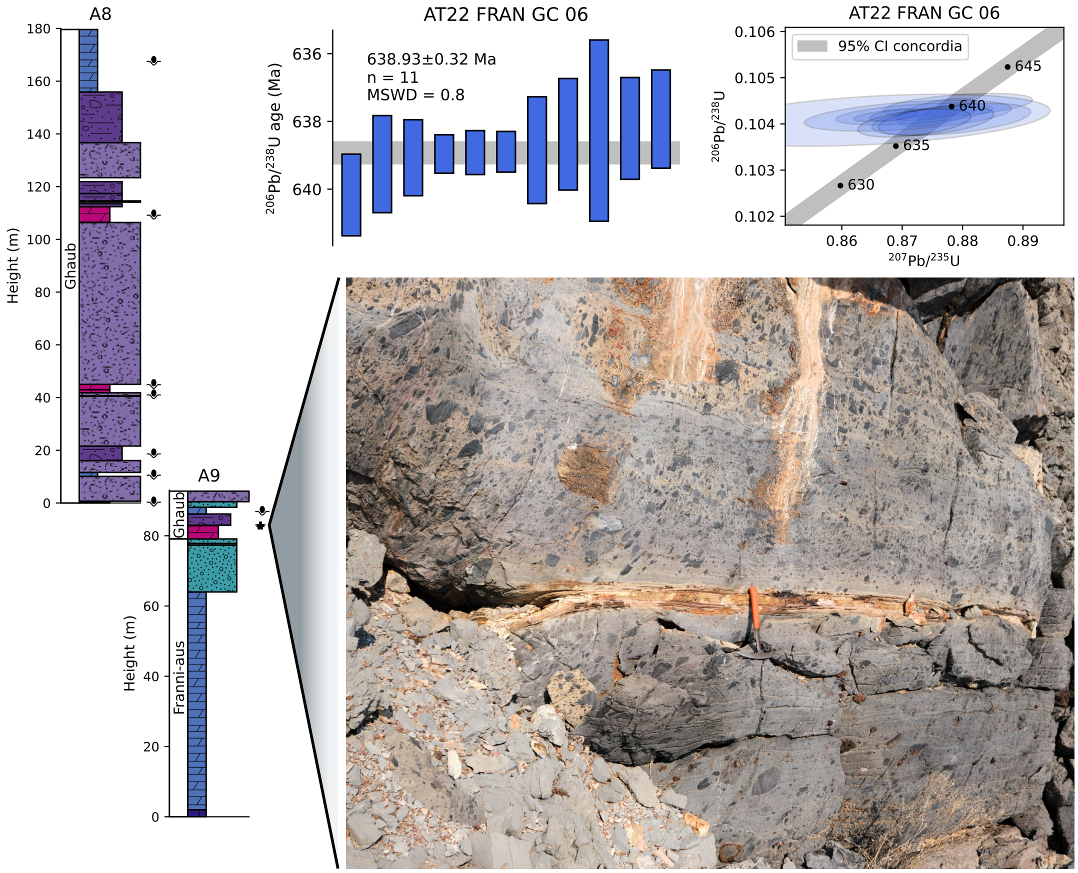

Fig. S21. CA-ID-TIMS analyses for AT22 FRAN GC 06, shown alongside sampling location and stratigraphic column.

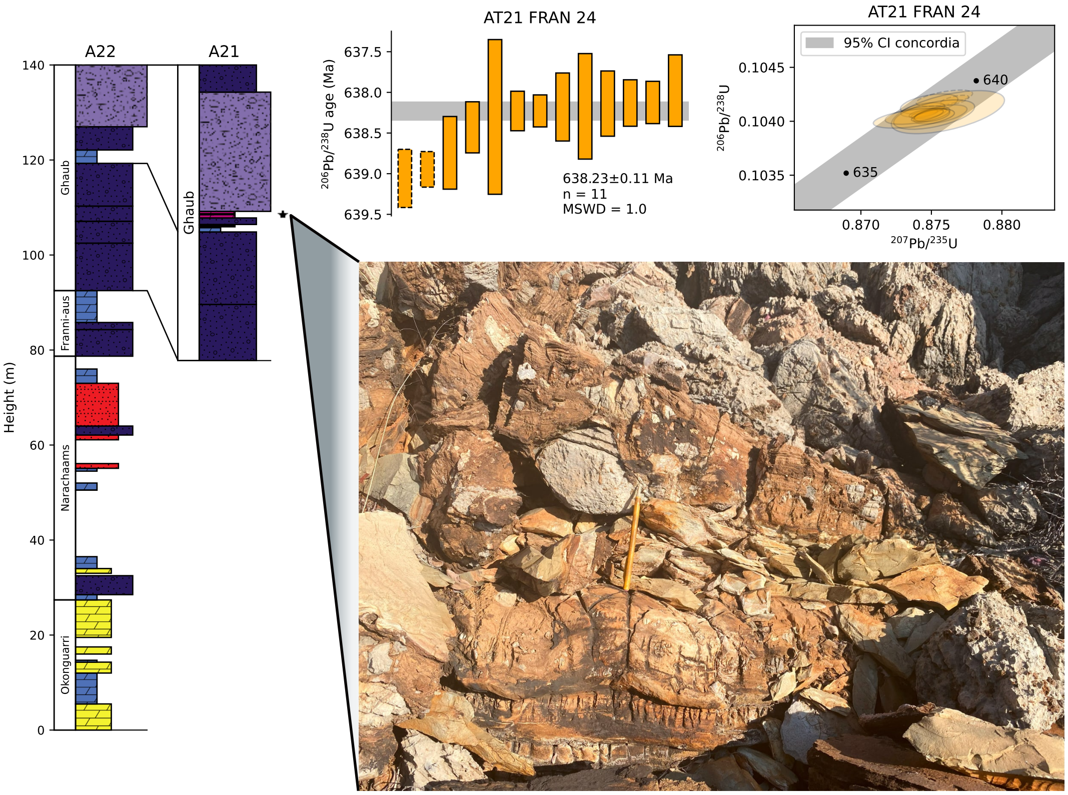

Fig. S22. CA-ID-TIMS analyses for AT21 FRAN 24, shown alongside sampling location and stratigraphic column A21. Dashed lines indicate analyses not included in the weighted mean. Section A22 is shown for further context illustrating the ambiguous underlying stratigraphy interpreted by others as Ghaub Fm.

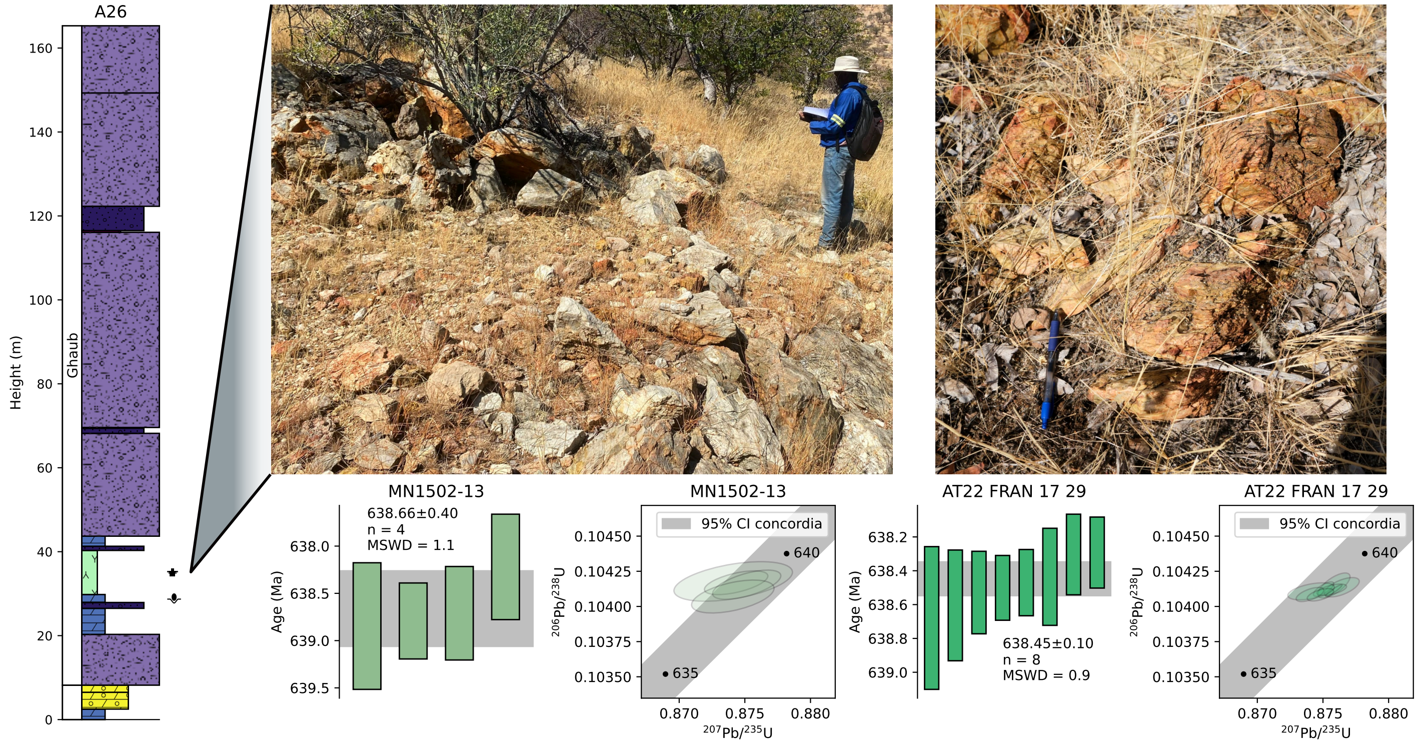

Fig. S23. CA-ID-TIMS analyses for AT22 FRAN 17 29 and MN1502-13. Also shown are photos of sample locations for AT22 FRAN 17 29 and MN1502-13, interpreted to be the same 10 m horizon of epidotized tuffaceous siltstone.

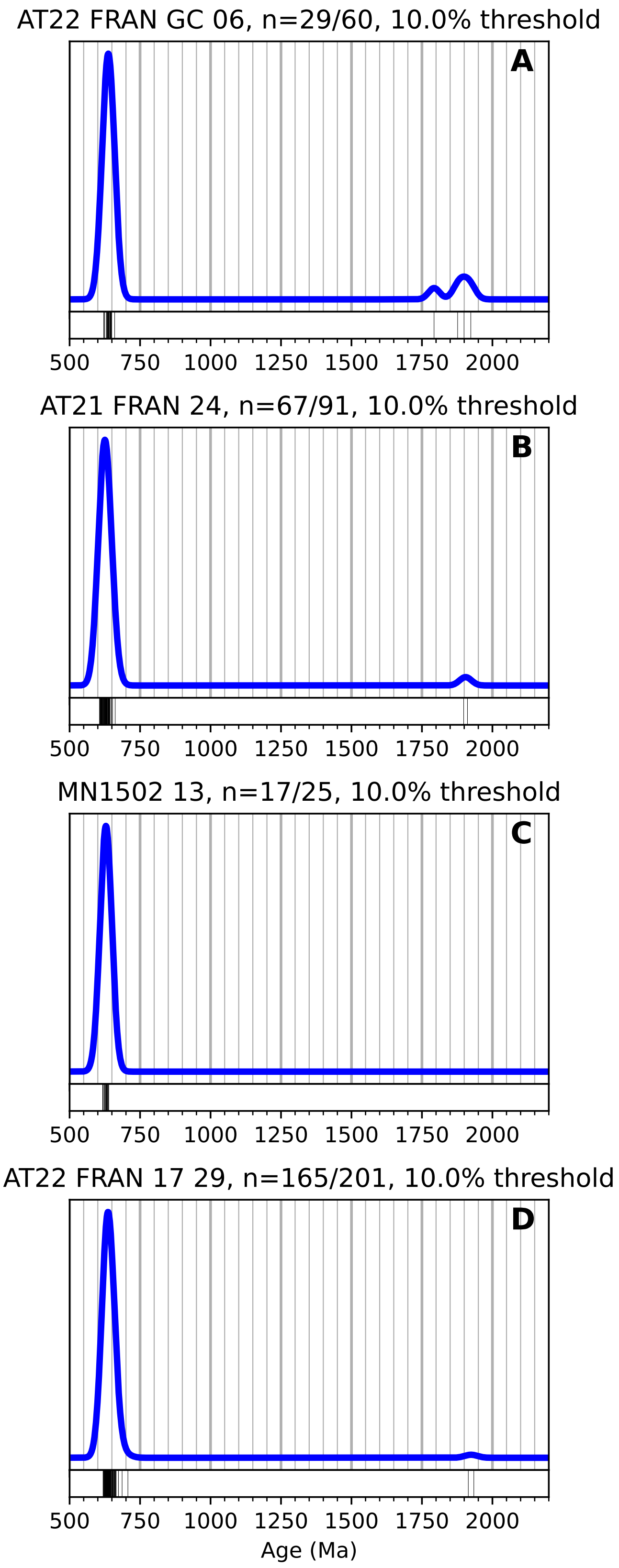

Fig. S24. Kernel density estimates for the four tuffaceous siltstone samples presented in the main manuscript, in which each sample exhibits a dominant young zircon age population. Rare Paleoproterozoic detrital grains are consistent in age with local basement sources.

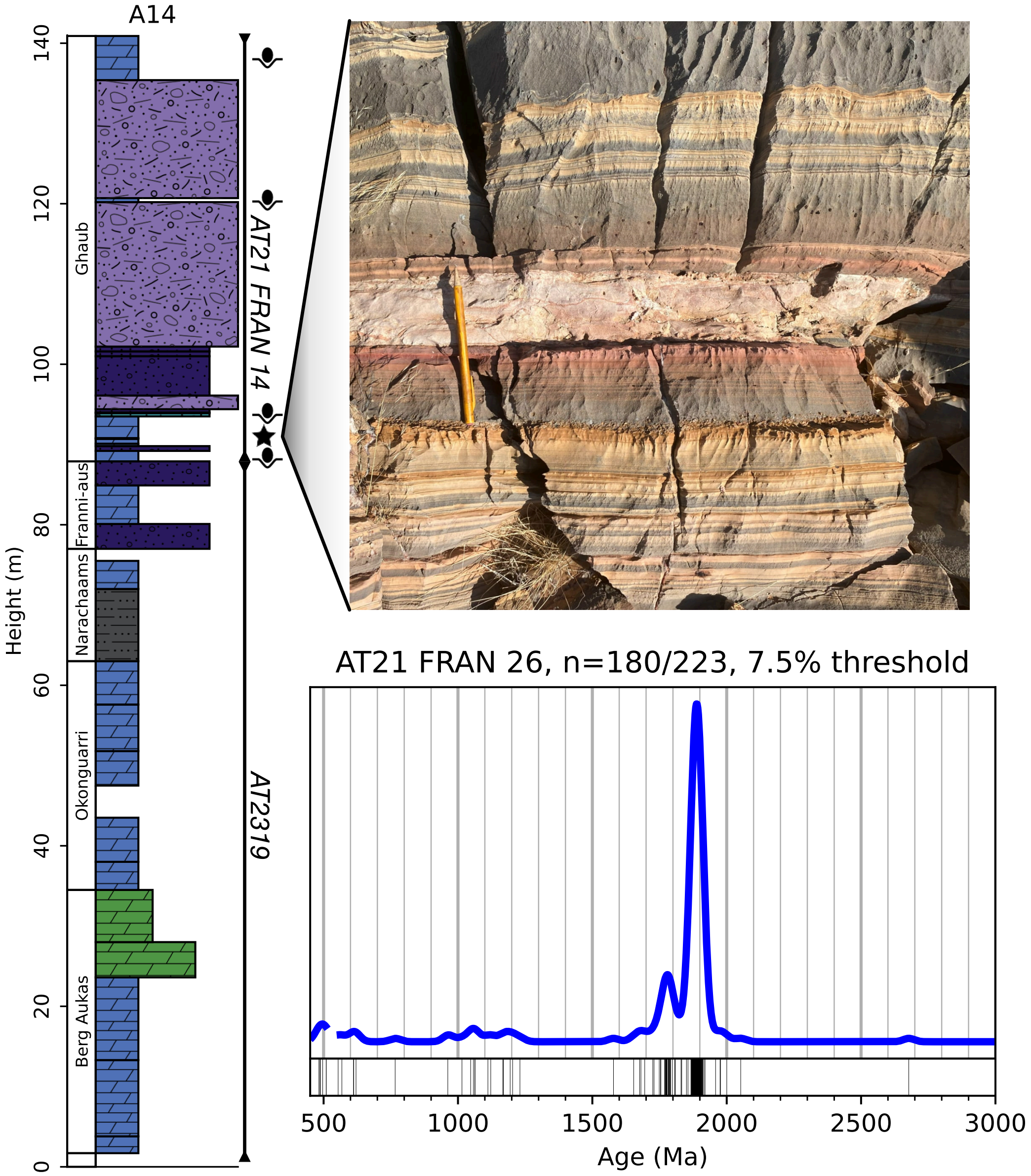

Fig. S25. Photo of sampled horizon and KDE for condordant analyses (7.5% relative age filter) for AT21 FRAN 26, shown in composite section A14.

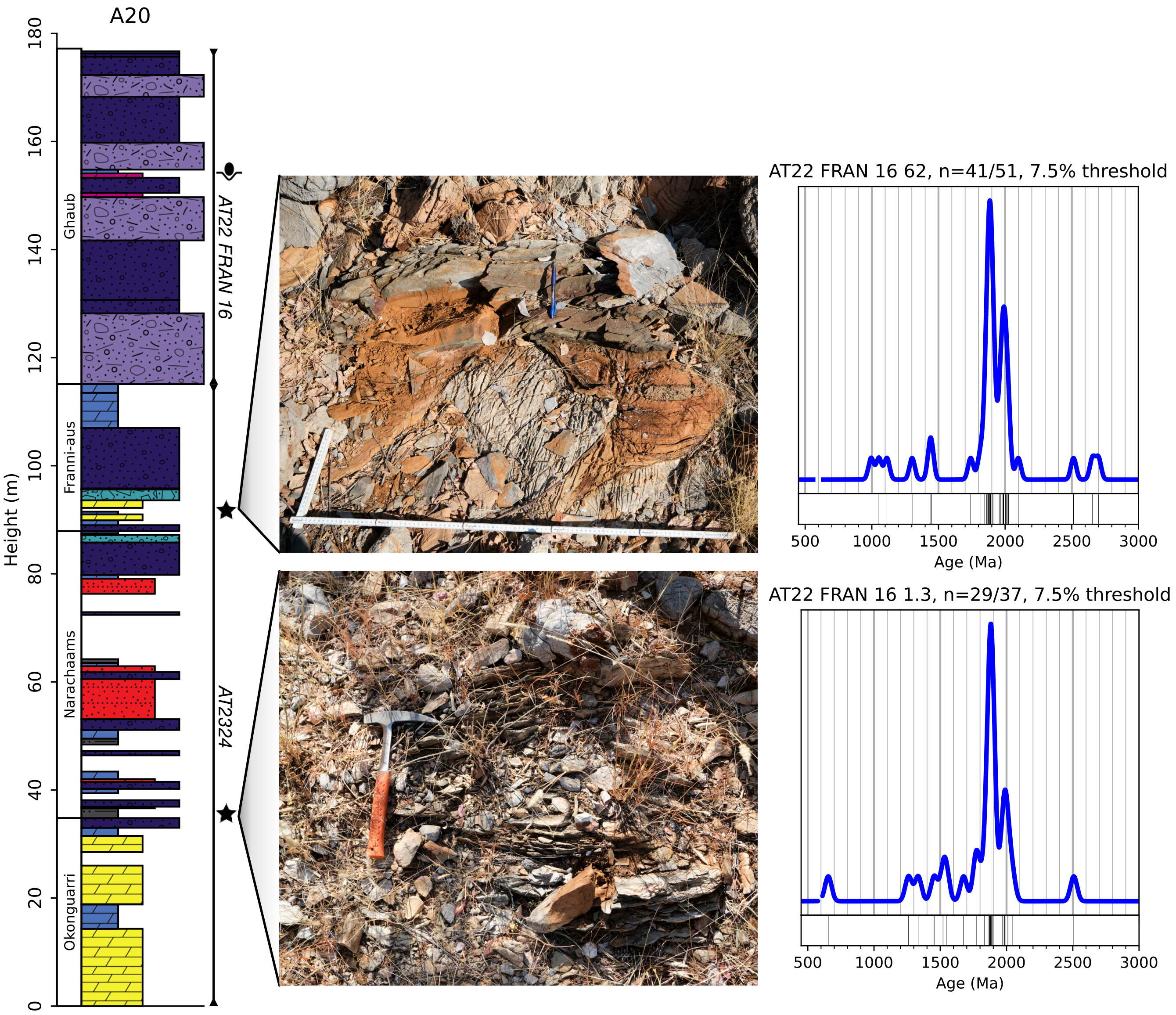

Fig. S26. Composite section AT20 with detrital samples AT22 FRAN 16 1.3 and AT22 FRAN 16 62. Tip of pen points up section, handle of hammer points up section. The measuring stick in the upper photo is set to a length of 1 m.

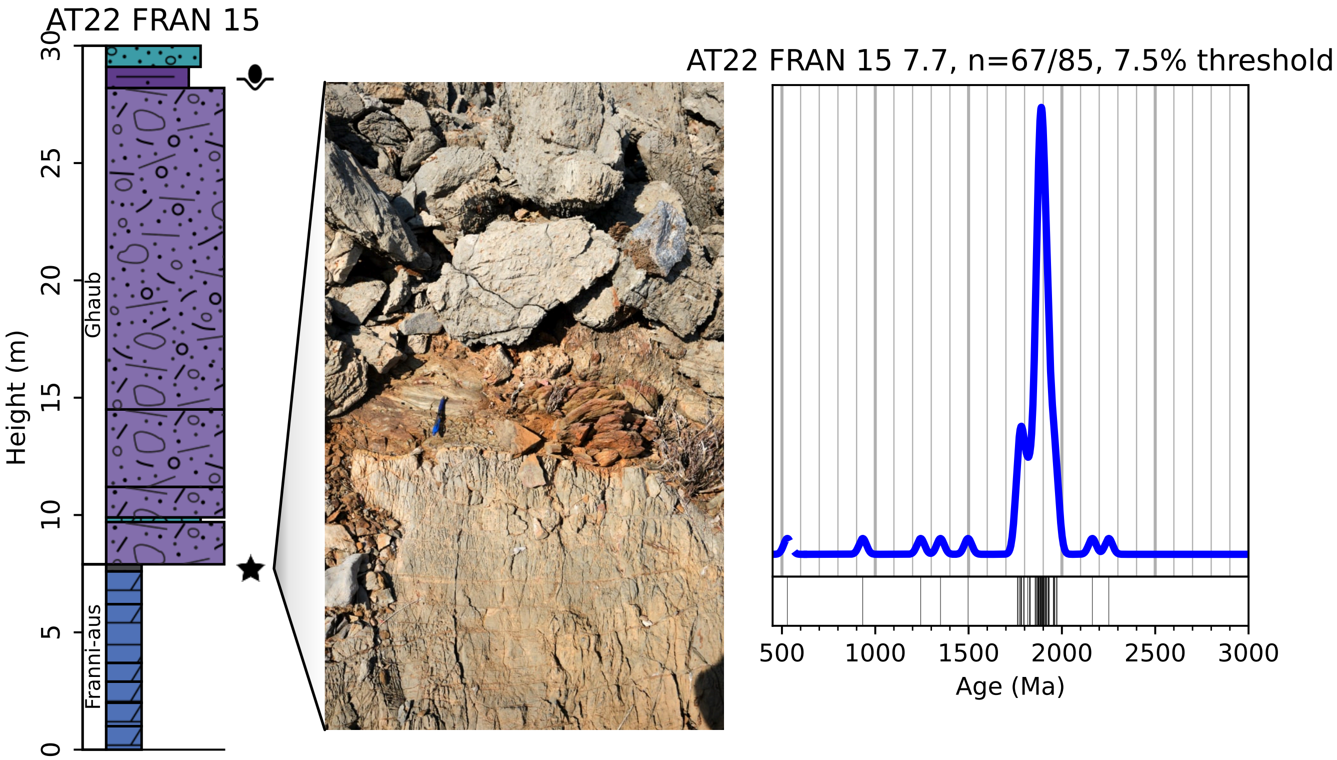

Fig. S27. Detrital sample AT22 FRAN 15 7.7, a green siltstone interpreted to be in Franni-aus Fm eroded by overlying massive diamictite of Ghaub Fm.

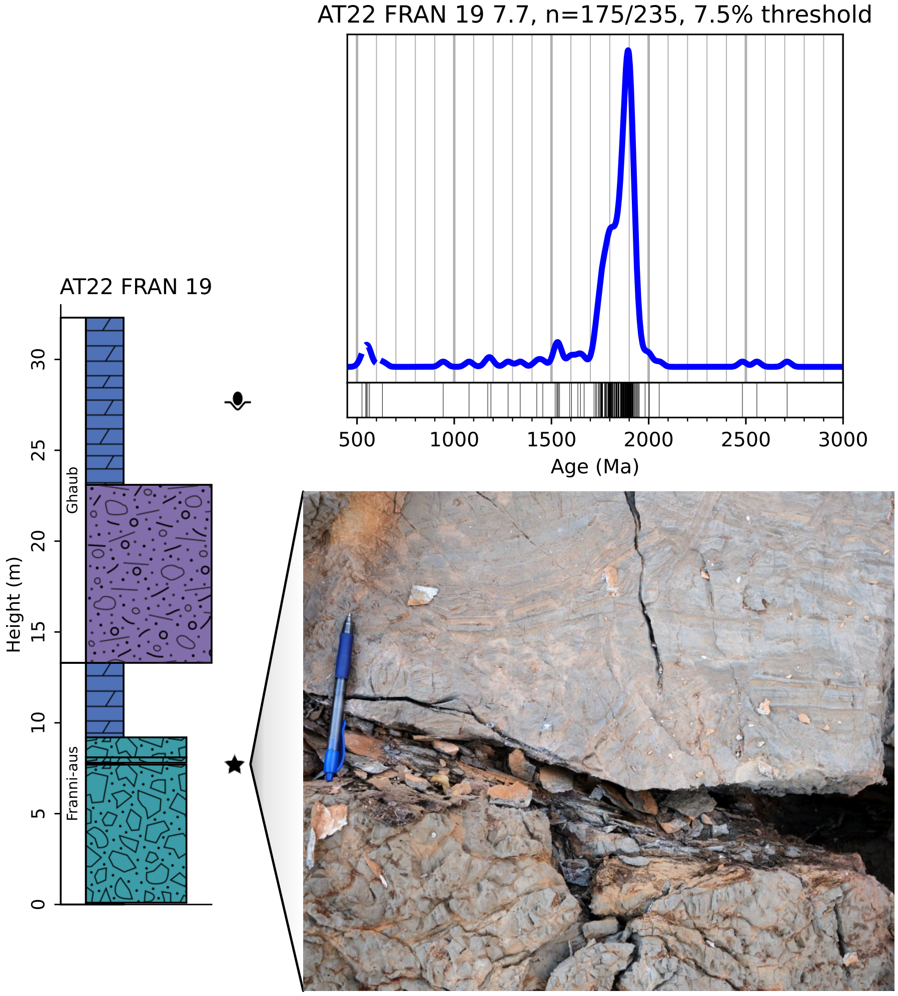

Fig. S28. Detrital sample AT22 FRAN 19 7.7, a green siltstone interpreted to be in Franni-aus Fm eroded by overlying massive diamictite of Ghaub Fm (similar stratigraphic context to AT22 FRAN 15 7.7).

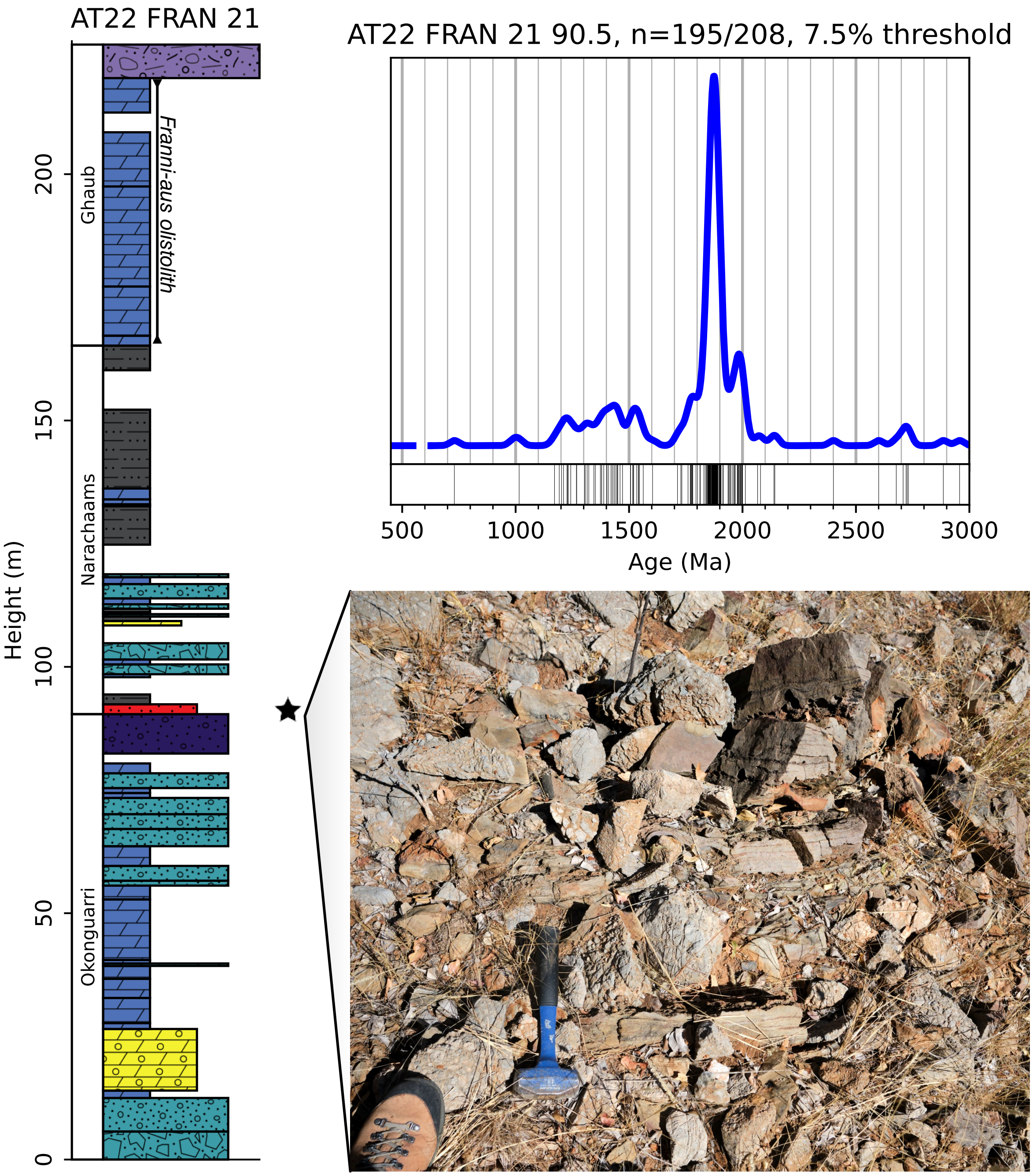

Fig. S29. Coarse green arkose interbedded with pebble–cobble dolomite grainstone sedimentary breccias, sampled as AT22 FRAN 21 90.5 at the base of Narachaams Fm (alternatively, Ghaub Fm).

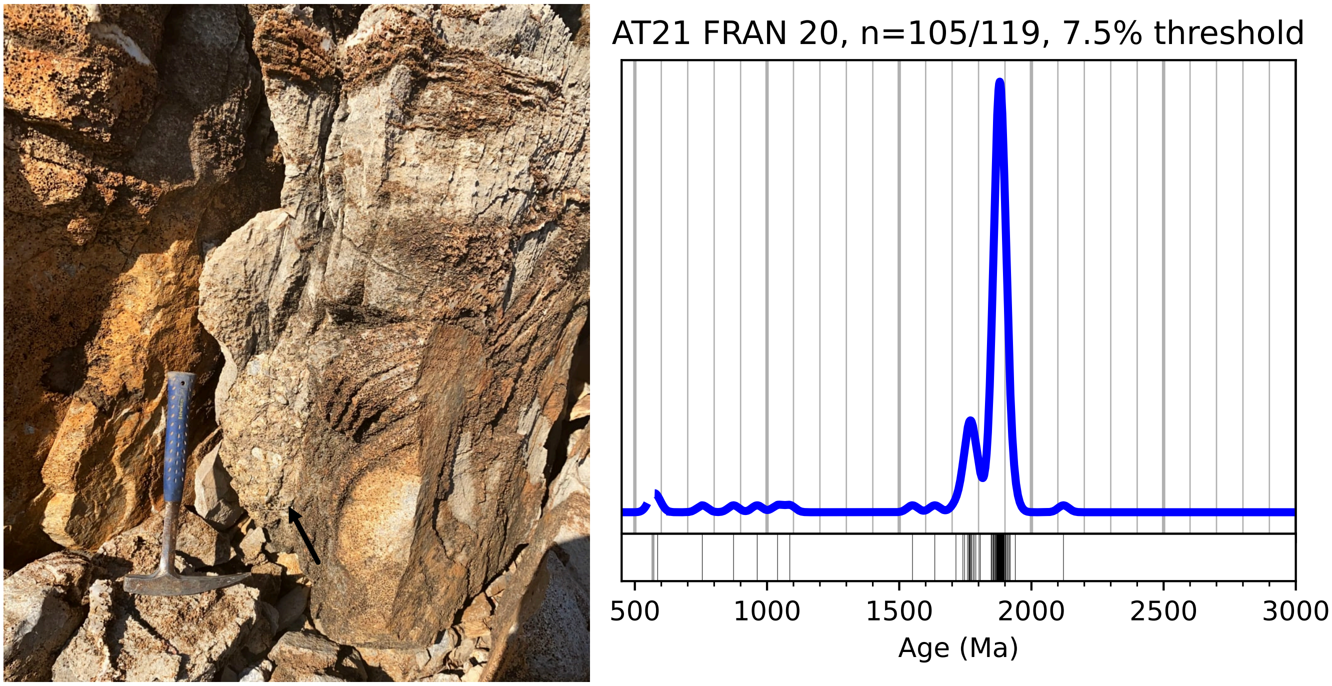

Fig. S30. Granule–cobble conglomerate of silicified oolite clasts and unsilicified dolosiltites and doloarenites within heavily silicified upper Franni-aus Fm within 10 m of basal Ghaub Fm. Sampled material is immediately left of the clast of silicified oolite with spheroidal texture.

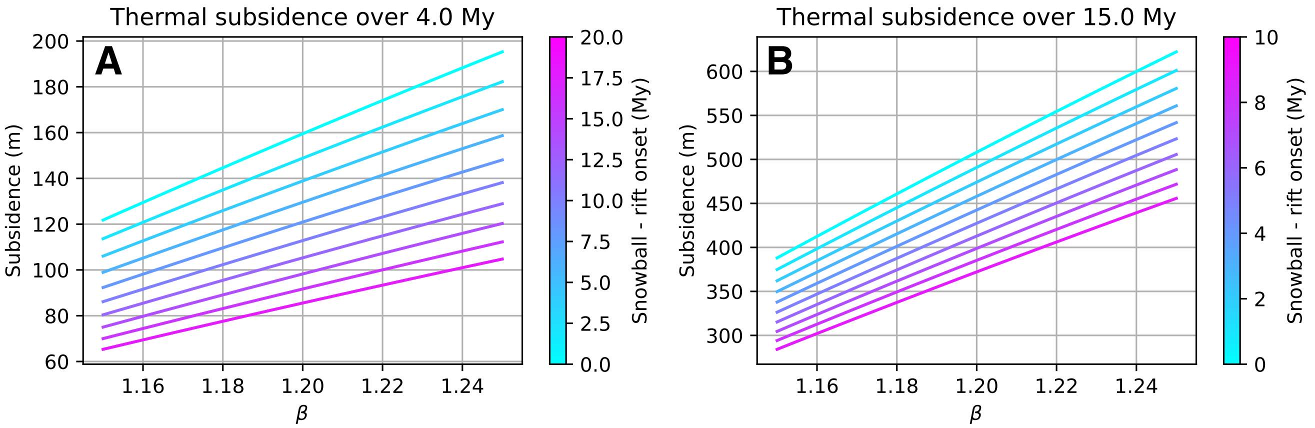

Fig. S31. Thermal subsidence models as per ref. (62) for southern Congo paleomargin during the Marinoan Snowball for 4 Myr (ca. 639 Ma onset **A**) and 15 Myr (ca. 650 Ma onset **B**) durations. Crustal thinning (β) factors vary from 1.15–1.25, as per ref. (39). Each line reflects a different time lag between end of rifting and onset of the Marinoan Snowball, with the range spanning the Middle Cryogenian right up to snowball onset. Models assume no sediment fill.

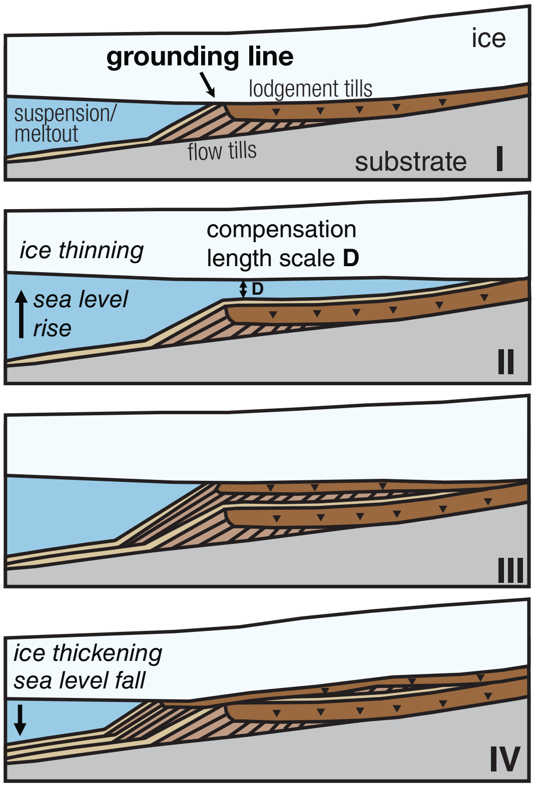

Fig. S32. Schematic of glaciomarine sedimentation over a retreat-advance cycle of the grounding line. Modified from ref. (88). **I** Beneath grounded ice, lodgement tills deposit as detritus melts out from the base of grounded, flowing ice. At the grounding line, these tills are reworked and redeposit as gravity flows that form a prograding wedge. Beyond the grounding line, sediments deposit via settling from suspension and melting out of the floating ice above. **II** The grounding line is sensitive to both ice thickness and local relative sea level. Ice thinning and/or local sea level rise causes lateral retreat of the grounding line, which also generates vertical accommodation. **III** This accommodation can be filled by the prograding grounding zone wedge. **IV** If ice thickens and/or local relative sea level falls, the grounding line will advance laterally. This advance increases the potential for glacial erosion in the section. Thus, the maximum topographic relief (D, panel II) associated with the process of sedimentation in this system is set by the maximum vertical amplitude of grounding line motion.

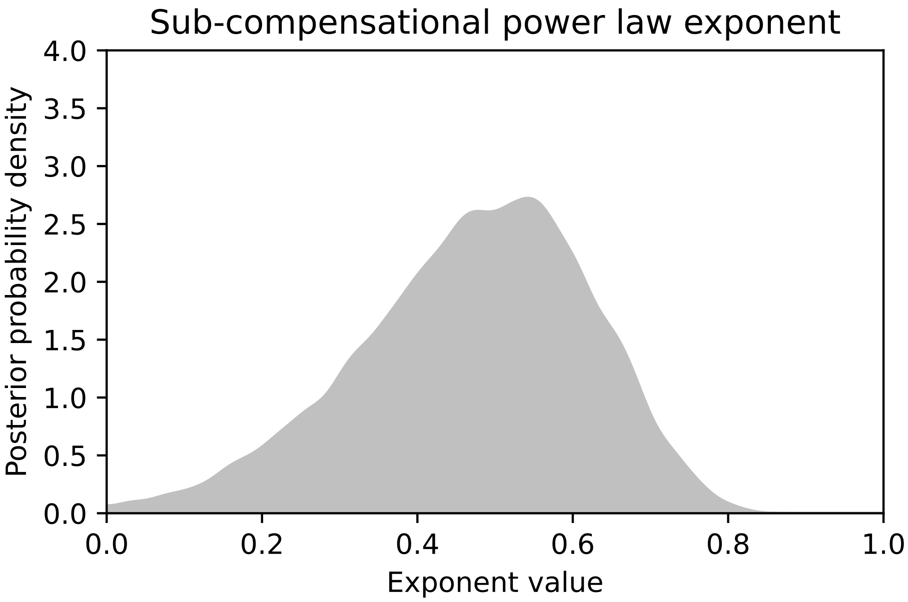

Fig. S33. Posterior distribution power law exponent κ of the variability in sedimentation σ_{ss} at length scales below the compensation length scale (CLS), i.e., the non-compensational regime. The modal value around 0.5 implies that glaciomarine sedimentation is largely independent from existing topography at sub-CLS length scales (89).

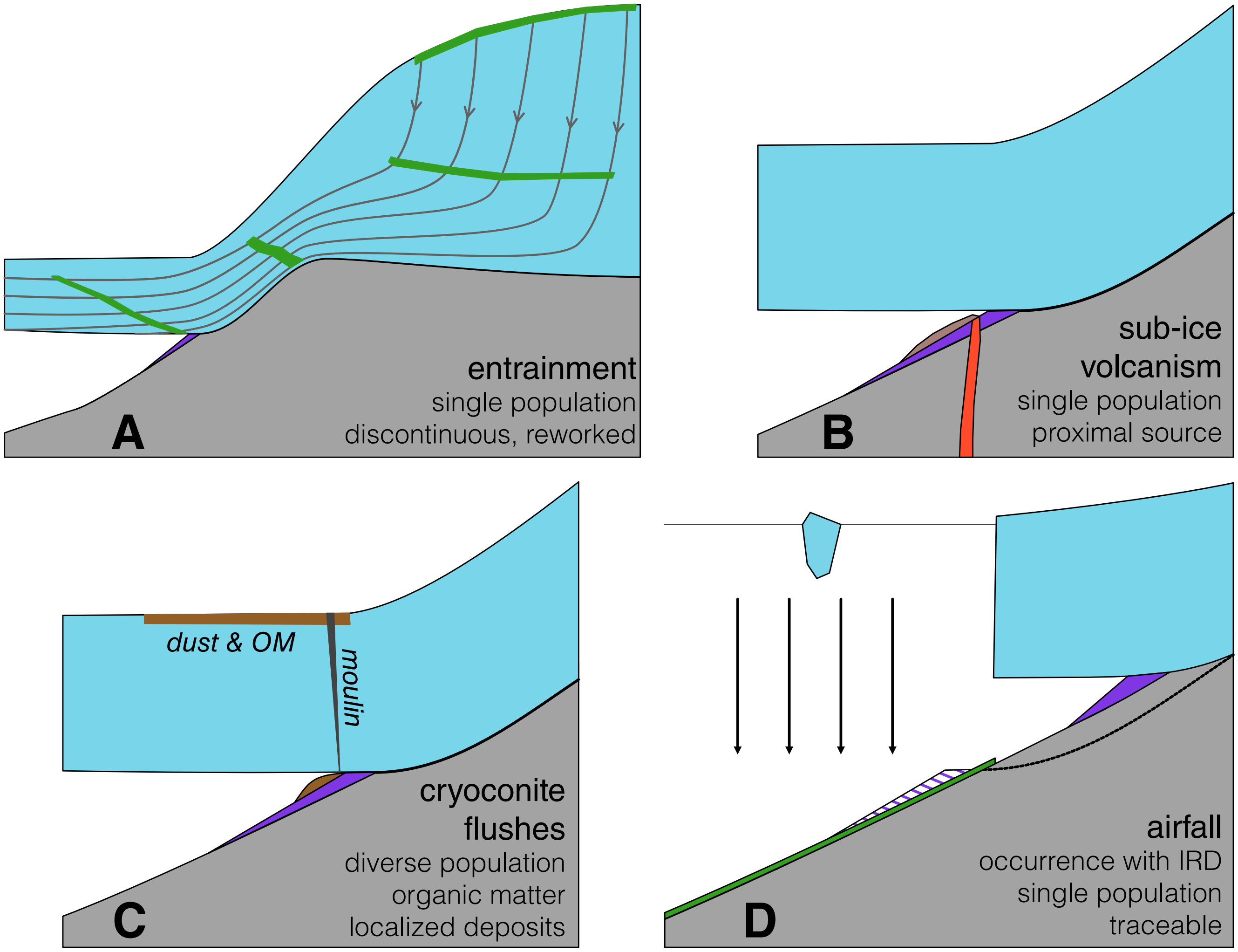

Fig. S34. Different models for delivery of volcanic material to the glaciomarine setting. **A** Entrainment of ash in the accumulation zone of the ice sheet. Ash transits the ice sheet as a coherent band that eventually intersects the base of the sea glacier, where it may melt out. **B** Proximal sub-marine volcanism. **C** Flushing through moulins of cryoconite that accumulates on the flat expanses of the sea glacier could contribute young volcanic material to the glaciomarine depositional setting (73). **D** If a low-latitude calving terrestrial ice front developed before sea glacier growth, dropstones could coexist with airfall volcanic ash in a glaciomarine setting. Dashed black line shows future extent of erosion; purple lines show future grounding zone wedge that records furthest snowball ice advance.

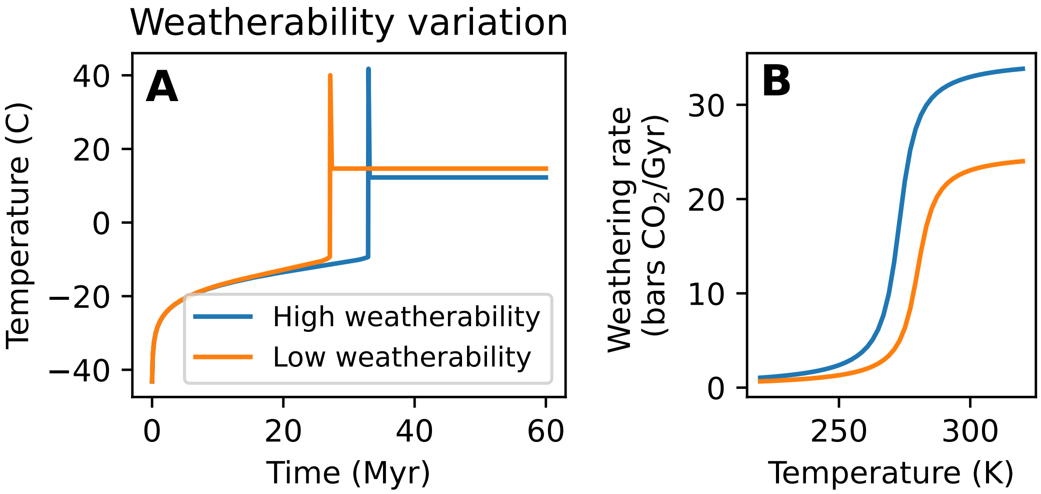

Fig. S35. **A** Minor change in Snowball duration for large changes in planetary weatherability (B). **B** High (blue) and low (orange) weatherability models.

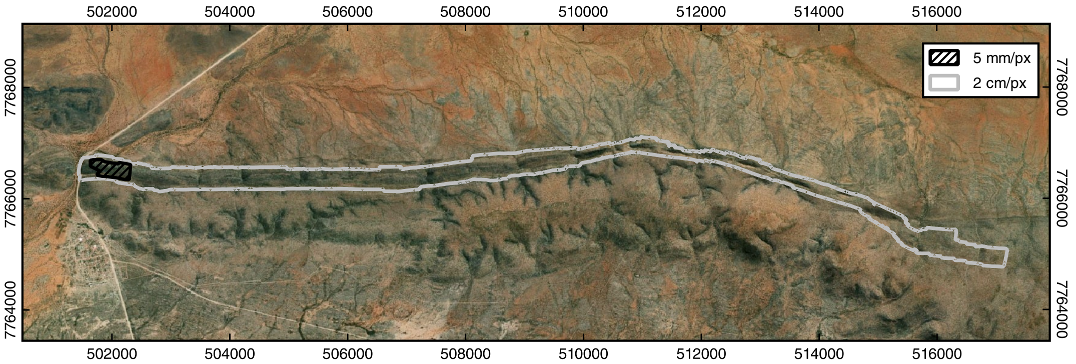

Fig. S36. Droned area footprints at Fransfontein Ridge.

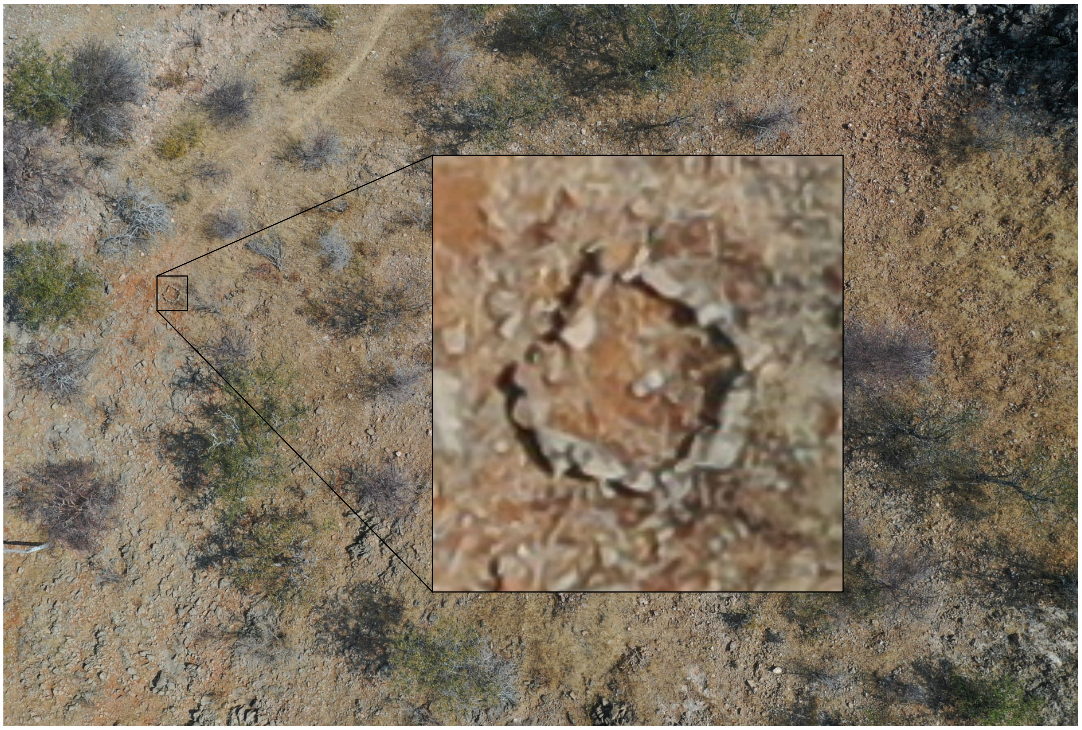

Fig. S37. Example of a ground control point (GCP) at Fransfontein, where the central rock is the surveyed point used as the GCP in Agisoft Metashape.

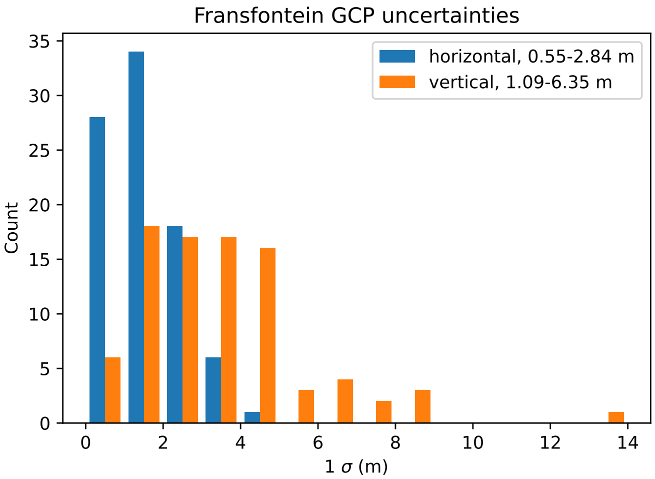

Fig. S38. Horizontal and vertical ground control point (GCP) uncertainties for 74 GCPs. The ranges are the 0.1 to 0.9 quantiles.

Table S1. Dictionary matching sections as referred to in the manuscript with section names as measured in the field. Manuscript labels increase as sections move from west to east.

Section Name	Manuscript Label	stratigraphy?
AT2315	A1	Y
AT21 FRAN 08	A2	
AT21 FRAN 07	A3	
AT21 FRAN 06	A4	
AT21 FRAN 09	A5	
AT21 FRAN 10	A6	
AT21 FRAN 11	A7	
AT21 FRAN 05	A8	
AT2313	A9	Y
AT21 FRAN 03	A10	
AT2316	A11	Y
AT2317	A12	
AT2318	A13	
AT2319	A14	Y
AT21 FRAN 14		
AT22 FRAN 19	A15	
AT2320	A16	Y
AT2321	A17	Y
AT2322	A18	Y
AT2323	A19	Y
AT2324	A20	Y
AT22 FRAN 16	A20	
AT21 FRAN 12	A21	
AT2325	A22	Y
AT22 FRAN 21	A23	Y
AT2326	A24	Y
AT22 FRAN 20	A25	Y
AT2327	A26	Y
AT22 FRAN 17		

Table S2. Sample coordinates and stratigraphic locations.

	Sample	Latitude	Longitude	Formation
Tuffaceous	AT22 FRAN GC 06	-20.199584	15.028950	basal Ghaub
	AT22 FRAN 17 29	-20.212136	15.163943	Ghaub
	MN1502 13	-20.212951	15.161858	Ghaub
	AT21 FRAN 24	-20.211147	15.150498	Ghaub, resampling of DW-1
Detrital	AT21 FRAN 20	-20.198003	15.019914	Franni-aus
	AT22 FRAN 15 7.7	-20.203496	15.137454	Franni-aus
	AT22 FRAN 16 1.3	-20.209565	15.149088	Narachaams or Ghaub
	AT22 FRAN 16 62	-20.210171	15.149022	Narachaams or Ghaub
	AT22 FRAN 19 7.7	-20.195205	15.100937	Franni-aus
	AT22 FRAN 21 90.5	-20.210877	15.154823	Narachaams or Ghaub
	AT21 FRAN 26	-20.195244	15.099158	Basal Ghaub

SI Dataset S1 (Dataset-S1_CA-ID-TIMS.xlsx)
U-Pb isotopic data for CA-ID-TIMS analyses of zircon.

SI Dataset S2 (Dataset-S2_LASS-ICPMS.xlsx)
U-Pb isotopic and trace element composition for LASS-ICPMS analyses of zircon.

SI Dataset S3 (Dataset-S3_Cryogenian-geochronology.xlsx)
Compilation of Cryogenian geochronology presented in Figure S1.

SI Dataset S4 (Dataset-S4_d13C.xlsx)
δ^{13}C and δ^{18}O measurements of carbonates presented in Figure S6.

References

1. AD Rooney, et al., Re-Os geochronology and coupled Os-Sr isotope constraints on the Sturtian snowball Earth. *Proc. Natl. Acad. Sci.* **111**, 51–56 (2014).
2. AD Rooney, JV Strauss, AD Brandon, FA Macdonald, A Cryogenian chronology: Two long-lasting synchronous Neoproterozoic glaciations. *Geology* **43**, 459–462 (2015).
3. M Brasier, et al., New U-Pb zircon dates for the Neoproterozoic Ghubrah glaciation and for the top of the Huqf Supergroup, Oman. *Geology* **28**, 4 (2000).
4. Z Lan, et al., Toward refining the onset age of Sturtian glaciation in South China. *Precambrian Res.* **338**, 105555 (2020).
5. S MacLennan, et al., The arc of the Snowball: U-Pb dates constrain the Islay anomaly and the initiation of the Sturtian glaciation. *Geology* **46**, 539–542 (2018).
6. GM Cox, et al., Kikiktat volcanics of Arctic Alaska—Melting of harzburgitic mantle associated with the Franklin large igneous province. *Lithosphere* **7**, 275–295 (2015).
7. FA Macdonald, et al., Cryogenian of Yukon. *Precambrian Res.* **319**, 114–143 (2018).
8. FA Macdonald, et al., Calibrating the Cryogenian. *Science* **327**, 1241–1243 (2010).
9. CM Fanning, PK Link, U-Pb SHRIMP ages of Neoproterozoic (Sturtian) glaciogenic Pocatello Formation, southeastern Idaho. *Geology* **32**, 881 (2004).
10. Z Lan, et al., A rapid and synchronous initiation of the wide spread Cryogenian glaciations. *Precambrian Res.* **255**, 401–411 (2014).
11. G Song, X Wang, X Shi, G Jiang, New U-Pb age constraints on the upper Banxi Group and synchrony of the Sturtian glaciation in South China. *Geosci. Front.* **8**, 1161–1173 (2017).
12. GM Cox, GP Halverson, S Denyszyn, J Foden, FA Macdonald, Cryogenian magmatism along the north-western margin of Laurentia: Plume or rift? *Precambrian Res.* **319**, 144–157 (2018).
13. SA Bowring, et al., Geochronologic constraints on the chronostratigraphic framework of the Neoproterozoic Huqf Supergroup, Sultanate of Oman. *Am. J. Sci.* **307**, 1097–1145 (2007).
14. GJ Baldwin, EC Turner, BS Kamber, Tectonic controls on distribution and stratigraphy of the Cryogenian Rapitan iron formation, northwestern Canada. *Precambrian Res.* **278**, 303–322 (2016).
15. LL Nelson, et al., Geochronological constraints on Neoproterozoic rifting and onset of the Marinoan glaciation from the Kingston Peak Formation in Death Valley, California (USA). *Geology* **48**, 1083–1087 (2020).
16. EJ Rugen, et al., Glacially influenced provenance and Sturtian affinity revealed by detrital zircon U–Pb ages from sandstones in the Port Askaig Formation, Dalradian Supergroup. *J. Geol. Soc.* **181**, jgs2024–029 (2024).
17. VH Isakson, MD Schmitz, CM Dehler, FA Macdonald, W Adolph Yonkee, A robust age model for the Cryogenian Pocatello Formation of southeastern Idaho (northwestern USA) from tandem in situ and isotope dilution U-Pb dating of volcanic tuffs and epiclastic detrital zircons. *Geosphere* **18**, 825–849 (2022).
18. A Eyster, F Ferri, MD Schmitz, FA Macdonald, One diamictite and two rifts: Stratigraphy and geochronology of the Gataga Mountain of northern British Columbia. *Am. J. Sci.* **318**, 167–207 (2018).
19. L Courtney-Davies, RM Flowers, CS Siddoway, A Tasistro-Hart, FA Macdonald, Hematite U-Pb dating of Snowball Earth meltwater events. *Proc. Natl. Acad. Sci.* **121**, e2410759121 (2024).
20. DJ Condon, SA Bowring, A user's guide to Neoproterozoic geochronology in *The Geological Record of Neoproterozoic Glaciations*, eds. E Arnaud, GP Halverson, G Shields-Zhou. (Geological Society of London) Vol. 36, p. 0 (2011).
21. JA Keeley, PK Link, CM Fanning, MD Schmitz, Pre- to synglacial rift-related volcanism in the Neoproterozoic (Cryogenian) Pocatello Formation, SE Idaho: New SHRIMP and CA-ID-TIMS constraints. *Lithosphere* **5**, 128–150 (2013).
22. K Lund, JN Aleinikoff, KV Evans, CM Fanning, SHRIMP U-Pb geochronology of Neoproterozoic Windermere Supergroup, central Idaho: Implications for rifting of western Laurentia and synchroneity of Sturtian glacial deposits. *Geol. Soc. Am. Bull.* **115**, 349–372 (2003).
23. K Lund, et al., SHRIMP U-Pb dating of recurrent Cryogenian and Late Cambrian-Early Ordovician alkalic magmatism in central Idaho: Implications for Rodinian rift tectonics. *Geol. Soc. Am. Bull.* **122**, 430–453 (2010).
24. GM Cox, et al., South Australian U-Pb zircon (CA-ID-TIMS) age supports globally synchronous Sturtian deglaciation. *Precambrian Res.* **315**, 257–263 (2018).

25. C Zhou, et al., New constraints on the ages of Neoproterozoic glaciations in south China. *Geology* **32**, 437 (2004).
26. W Yu, et al., Newly discovered Sturtian cap carbonate in the Nanhua Basin, South China. *Precambrian Res.* **293**, 112–130 (2017).
27. L Xu, et al., Termination of Sturtian glaciation with protracted, multiple volcanic eruptions. *Earth-Science Rev.* **255**, 104826 (2024).
28. AD Rooney, C Yang, DJ Condon, M Zhu, FA Macdonald, U-Pb and Re-Os geochronology tracks stratigraphic condensation in the Sturtian snowball Earth aftermath. *Geology* **48**, 625–629 (2020).
29. C Zhou, MH Huyskens, X Lang, S Xiao, QZ Yin, Calibrating the terminations of Cryogenian global glaciations. *Geology* **47**, 251–254 (2019).
30. B Kendall, RA Creaser, D Selby, Re-Os geochronology of postglacial black shales in Australia: Constraints on the timing of "Sturtian" glaciation. *Geology* **34**, 729 (2006).
31. S Zhang, G Jiang, Y Han, The age of the Nantuo Formation and Nantuo glaciation in South China. *Terra Nova* **20**, 289–294 (2008).
32. B Kendall, RA Creaser, CR Calver, TD Raub, DA Evans, Correlation of Sturtian diamictite successions in southern Australia and northwestern Tasmania by Re–Os black shale geochronology and the ambiguity of "Sturtian"-type diamictite–cap carbonate pairs as chronostratigraphic marker horizons. *Precambrian Res.* **172**, 301–310 (2009).
33. AR Prave, DJ Condon, KH Hoffmann, S Tapster, AE Fallick, Duration and nature of the end-Cryogenian (Marinoan) glaciation. *Geology* **44**, 631–634 (2016).
34. C Calver, et al., Globally synchronous Marinoan deglaciation indicated by U-Pb geochronology of the Cottons Breccia, Tasmania, Australia. *Geology* **41**, 1127–1130 (2013).
35. KH Hoffmann, D Condon, S Bowring, J Crowley, U-Pb zircon date from the Neoproterozoic Ghaub Formation, Namibia: Constraints on Marinoan glaciation. *Geology* **32**, 817 (2004).
36. D Condon, et al., U-Pb Ages from the Neoproterozoic Doushantuo Formation, China. *Science* **308**, 95–98 (2005).
37. GP Halverson, BP Wade, MT Hurtgen, KM Barovich, Neoproterozoic chemostratigraphy. *Precambrian Res.* **182**, 337–350 (2010).
38. CV Rose, et al., Constraints on the origin and relative timing of the Trezona δ13C anomaly below the end-Cryogenian glaciation. *Earth Planet. Sci. Lett.* **319–320**, 241–250 (2012).
39. GP Halverson, PF Hoffman, DP Schrag, AJ Kaufman, A major perturbation of the carbon cycle before the Ghaub glaciation (Neoproterozoic) in Namibia: Prelude to snowball Earth? *Geochem. Geophys. Geosystems* **3**, 1–24 (2002).
40. X Bao, et al., Cyclostratigraphic constraints on the duration of the Datangpo Formation and the onset age of the Nantuo (Marinoan) glaciation in South China. *Earth Planet. Sci. Lett.* **483**, 52–63 (2018).
41. PF Hoffman, et al., Snowballs in Africa: Sectioning a long-lived Neoproterozoic carbonate platform and its bathyal foreslope (NW Namibia). *Earth-Science Rev.* **219**, 103616 (2021).
42. EW Domack, PF Hoffman, An ice grounding-line wedge from the Ghaub glaciation (635 Ma) on the distal foreslope of the Otavi carbonate platform, Namibia, and its bearing on the snowball Earth hypothesis. *Geol. Soc. Am. Bull.* **123**, 1448–1477 (2011).
43. PF Hoffman, 28th DeBeers Alex. Du Toit Memorial Lecture, 2004. On Cryogenian (Neoproterozoic) ice-sheet dynamics and the limitations of the glacial sedimentary record. *South Afr. J. Geol.* **108**, 557–577 (2005).
44. PF Hoffman, Strange bedfellows: Glacial diamictite and cap carbonate from the Marinoan (635 Ma) glaciation in Namibia: Strange bedfellows. *Sedimentology* **58**, 57–119 (2011).
45. AR Kylander-Clark, BR Hacker, JM Cottle, Laser-ablation split-stream ICP petrochronology. *Chem. Geol.* **345**, 99–112 (2013).
46. T Krogh, A low-contamination method for hydrothermal decomposition of zircon and extraction of U and Pb for isotopic age determinations. *Geochimica et Cosmochimica Acta* **37**, 485–494 (1973).
47. H Gerstenberger, G Haase, A highly effective emitter substance for mass spectrometric Pb isotope ratio determinations. *Chem. Geol.* **136**, 309–312 (1997).
48. MD Schmitz, B Schoene, Derivation of isotope ratios, errors, and error correlations for U-Pb geochronology using ^{205}Pb-^{235}U-(^{233}U)-spiked isotope dilution thermal ionization mass spectrometric data: U-PB ISOTOPE RATIO DERIVATION. *Geochem. Geophys. Geosystems* **8**, n/a–n/a (2007).
49. D Condon, B Schoene, N McLean, S Bowring, R Parrish, Metrology and traceability of U–Pb isotope dilution geochronology (EARTHTIME Tracer Calibration Part I). *Geochimica et Cosmochimica Acta* **164**, 464–480 (2015).
50. AH Jaffey, KF Flynn, LE Glendenin, WC Bentley, AM Essling, Precision Measurement of Half-Lives and Specific Activities of U 235 and U 238. *Phys. Rev. C* **4**, 1889–1906 (1971).
51. J Hiess, DJ Condon, N McLean, SR Noble, ^{238} U/ ^{235} U Systematics in Terrestrial Uranium-Bearing Minerals. *Science* **335**, 1610–1614 (2012).
52. J Crowley, B Schoene, S Bowring, U-Pb dating of zircon in the Bishop Tuff at the millennial scale. *Geology* **35**, 1123 (2007).
53. KR Ludwig, User's manual for IsoPlot 3.0, Technical report (2003).
54. I Wendt, C Carl, The statistical distribution of the mean squared weighted deviation. *Chem. Geol. Isot. Geosci. section* **86**, 275–285 (1991).
55. SD Johnson, M Poujol, AF Kisters, Constraining the timing and migration of collisional tectonics in the Damara Belt,

Namibia: U-Pb zircon ages for the syntectonic Salem-type Stinkbank granite. *South Afr. J. Geol.* **109**, 611–624 (2006).
56. IC Kleinhanns, et al., U–Pb zircon ages and (isotope) geochemical signatures of the Kamanjab Inlier (NW Namibia): Constraints on Palaeoproterozoic crustal evolution along the southern Congo craton. *Geol. Soc. London, Special Publ.* **389**, 165–195 (2015).
57. A Kröner, Y Rojas-Agramonte, Mesoproterozoic (Grenville-age) granitoids and supracrustal rocks in Kaokoland, northwestern Namibia. *Precambrian Res.* **298**, 572–592 (2017).
58. J Lehmann, GM Bybee, B Hayes, TM Owen-Smith, G Belyanin, Emplacement of the giant Kunene AMCG complex into a contractional ductile shear zone and implications for the Mesoproterozoic tectonic evolution of SW Angola. *Int. J. Earth Sci.* **109**, 1463–1485 (2020).
59. MJ De Wit, B Linol, Precambrian Basement of the Congo Basin and Its Flanking Terrains in *Geology and Resource Potential of the Congo Basin*, eds. MJ De Wit, F Guillocheau, MCJ De Wit. (Springer Berlin Heidelberg, Berlin, Heidelberg), pp. 19–37 (2015).
60. PF Hoffman, AJ Kaufman, GP Halverson, Comings and Goings of Global Glaciations on a Neoproterozoic Tropical Platform in Namibia. *GSA TODAY* **8**, 1–9 (1998).
61. PF Hoffman, Glacial erosion on a snowball Earth: Testing for bias in flux balance, geographic setting, and tectonic regime. *Can. J. Earth Sci.* **60**, 765–777 (2023).
62. D McKenzie, Some remarks on the development of sedimentary basins. *Earth Planet. Sci. Lett.* **40**, 25–32 (1978).
63. F Nishio, T Katsushima, H Ohmae, Volcanic Ash Layers in Bare Ice Areas near the Yamato Mountains, Dronning Maud Land and the Allan Hills, Victoria Land, Antarctica. *Annals Glaciol.* **7**, 34–41 (1985).
64. P Curzio, L Folco, M Ada Laurenzi, M Mellini, A Zeoli, A tephra chronostratigraphic framework for the Frontier Mountain blue-ice field (northern Victoria Land, Antarctica). *Quat. Sci. Rev.* **27**, 602–620 (2008).
65. EL Newland, N Mingotti, AW Woods, Dynamics of deep-submarine volcanic eruptions. *Sci. Reports* **12**, 3276 (2022).
66. SL Walker, et al., Eruption-fed particle plumes and volcaniclastic deposits at a submarine volcano: NW Rota-1, Mariana Arc. *J. Geophys. Res. Solid Earth* **113**, 2007JB005441 (2008).
67. A Verolino, JDL White, M Brenna, Eruption dynamics at Pahvant Butte volcano, Utah, western USA: Insights from ash-sheet dispersal, grain size, and geochemical data. *Bull. Volcanol.* **80**, 81 (2018).
68. T Huang, A volcanic sedimentation model: Implications of processes and responses of deep-sea ashes. *Mar. Geol.* **38**, 103–122 (1980).
69. JD White, Subaqueous eruption-fed density currents and their deposits. *Precambrian Res.* **101**, 87–109 (2000).
70. B Kneller, MM Nasr-Azadani, S Radhakrishnan, E Meiburg, Long-range sediment transport in the world's oceans by stably stratified turbidity currents: LONG-RANGE TRANSPORT IN THE OCEAN. *J. Geophys. Res. Ocean.* **121**, 8608–8620 (2016).
71. AF Richards, Transpacific distribution of floating pumice from Isla San Benedicto, Mexico. *Deep. Sea Res. (1953)* **5**, 29–35 (1958).
72. M Jutzeler, et al., On the fate of pumice rafts formed during the 2012 Havre submarine eruption. *Nat. Commun.* **5**, 3660 (2014).
73. PF Hoffman, et al., Snowball Earth climate dynamics and Cryogenian geology-geobiology. *Sci. Adv.* **3**, e1600983 (2017).
74. DS Abbot, et al., Robust elements of Snowball Earth atmospheric circulation and oases for life. *J. Geophys. Res. Atmospheres* **118**, 6017–6027 (2013).
75. D Li, RT Pierrehumbert, Sea glacier flow and dust transport on Snowball Earth. *Geophys. Res. Lett.* **38**, n/a–n/a (2011).
76. D Pollard, JF Kasting, Climate-Ice Sheet Simulations of Neoproterozoic Glaciation Before and After Collapse to Snowball Earth in *Geophysical Monograph Series*, eds. GS Jenkins, MA McMenamin, CP McKay, L Sohl. (American Geophysical Union, Washington, D. C.), pp. 91–105 (2004).
77. Y Donnadieu, F Fluteau, G Ramstein, C Ritz, J Besse, Is there a conflict between the Neoproterozoic glacial deposits and the snowball Earth interpretation: An improved understanding with numerical modeling. *Earth Planet. Sci. Lett.* **208**, 101–112 (2003).
78. AJ Campbell, ED Waddington, SG Warren, Refugium for surface life on Snowball Earth in a nearly-enclosed sea? A first simple model for sea-glacier invasion. *Geophys. Res. Lett.* **38**, n/a–n/a (2011).
79. E Tziperman, et al., Continental constriction and oceanic ice-cover thickness in a Snowball-Earth scenario. *J. Geophys. Res. Ocean.* **117**, n/a–n/a (2012).
80. P Hoffman, On the kinematics and timing of Rodinia breakup: A possible rift–transform junction of Cryogenian age at the southwest cape of Congo Craton (northwest Namibia). *South Afr. J. Geol.* **124**, 401–420 (2021).
81. J Konopásek, KH Hoffmann, J Sláma, J Košler, The onset of flysch sedimentation in the Kaoko Belt (NW Namibia) – Implications for the pre-collisional evolution of the Kaoko–Dom Feliciano–Gariep orogen. *Precambrian Res.* **298**, 220–234 (2017).
82. NM Nieminski, M Grove, DR Lowe, Provenance of the Neoproterozoic deep-water Zerrissene Group of the Damara Orogen, Namibia, and paleogeographic implications for the closing of the Adamastor Ocean and assembly of the Gondwana supercontinent. *GSA Bull.* **131**, 355–371 (2019).
83. RD Müller, J Cannon, S Williams, A Dutkiewicz, PyBacktrack 1.0: A Tool for Reconstructing Paleobathymetry on Oceanic and Continental Crust. *Geochem. Geophys. Geosystems* **19**, 1898–1909 (2018).
84. RM Miller, Neoproterozoic and early Palaeozoic rocks of the Damara Orogen. *The geology Namib.* **2**, 13–1 (2008).

85. O Abril-Pla, et al., PyMC: A modern, and comprehensive probabilistic programming framework in Python. *PeerJ Comput. Sci.* **9**, e1516 (2023).
86. T Pescarini, RI Trindade, PF Hoffman, LG Sant'Anna, Paleomagnetic investigation of the basal Maieberg Formation (Namibia) cap carbonate sequence (635 Ma): Implications for Snowball Earth postglacial dynamics. *Geol. Soc. Am. Bull.* **136**, 4775–4797 (2024).
87. CB Grimes, JL Wooden, MJ Cheadle, BE John, "Fingerprinting" tectono-magmatic provenance using trace elements in igneous zircon. *Contributions to Mineral. Petrol.* **170**, 46 (2015).
88. GS Boulton, Sedimentary and sea level changes during glacial cycles and their control on glacimarine facies architecture. *Geol. Soc. London, Special Publ.* **53**, 15–52 (1990).
89. KM Straub, C Paola, D Mohrig, MA Wolinsky, T George, Compensational Stacking of Channelized Sedimentary Deposits. *J. Sedimentary Res.* **79**, 673–688 (2009).
